# Substrate Epoxidation Catalyzed by the Nonheme Iron Dioxygenase Dapdiamide Biosynthesis Enzyme C. Why Is the Substrate Tethered?

**DOI:** 10.1002/chem.202501610

**Published:** 2025-08-21

**Authors:** Jingyu Cao, Sam P. de Visser

**Affiliations:** ^1^ Manchester Institute of Biotechnology The University of Manchester 131 Princess Street Manchester M1 7DN UK; ^2^ Department of Chemical Engineering The University of Manchester Oxford Road Manchester M13 9PL UK

**Keywords:** computational chemistry, density functional theory, enzyme catalysis, epoxidation, inorganic reaction mechanisms

## Abstract

Enzymes usually react with a free substrate, although some examples have appeared in the literature of enzymes that utilize a substrate tethered to a protein carrier. However, it is not clear what advantage the tethering has, and therefore a computational study was performed. In particular, we report here the first computational study on the nonheme iron dioxygenase involved in the epoxidation reaction during the dapdiamide biosynthesis (DdaC) and investigate tethered and nontethered substrates. Molecular dynamics (MD) simulations show that the protein carrier applies pressure onto the surface of the protein and influences the fold and active site description and leads to differences in substrate‐oxidant interactions. Quantum chemical calculations give much lower epoxidation barriers for the activation of the tethered substrate by 7 kcal mol^−1^ over the nontethered substrate and highlight the advantage of a tethered substrate in catalysis.

## Graphical abstract

In this work a computational study shows the differences in binding and catalysis of a nonheme iron oxygenase with tethered versus nontethered substrate. The tethered substrate is positioned in an ideal orientation near the active site, while the nontethered substrate shows more dynamics and is not positioned well for double bond epoxidation.

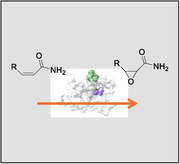

## 1 Introduction

Nonheme iron oxygenases are common enzymes in nature involved in the biosynthesis of natural products. Natural products are essential biomolecules for the organism and help with its functioning and its defense.^[^ ^1^ ^]^ Many nonheme iron oxygenases catalyze a regio‐ or even stereoselective reaction mechanism that creates a unique natural product with high yield.^[^ ^2^ ^]^ Understanding the mechanisms of these natural product synthesis reactions is, therefore, important, as it may assist in the design of biotechnological approaches for the synthesis of valuable materials, for instance, for pharmaceutical applications. Within the class of nonheme iron dioxygenases are the α‐ketoglutarate (αKG)‐dependent dioxygenases, which have application in natural product synthesis. They use O_2_ and αKG on an iron(II) center to create a high‐valent iron(IV)‐oxo intermediate in their catalytic cycle as an oxidant for an oxygen atom transfer reaction to substrate.^[^ ^3^ ^]^ Although, generally, the αKG‐dependent nonheme iron dioxygenases react through aliphatic hydroxylation of their substrate, evidence has emerged of a broader set of reactions that includes, for instance, the desaturation of the substrate through ring closure or double bond formation.^[^ ^4^ ^]^ In addition, some αKG‐dependent nonheme iron dioxygenases have been shown to react with C═C double bonds to form an epoxide product.^[^ ^5^ ^]^ Thus, the fungal dioxygenase AsqJ installs an epoxide ring into benzo[1,4]diazepine‐2,5‐dione as part of the antibiotic biosynthesis of quinolone.^[^ ^6^ ^]^

**Scheme 1:**
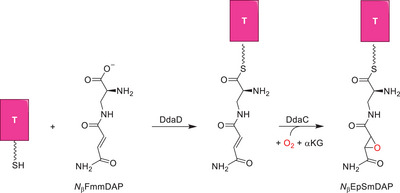
Epoxide ring formation catalyzed by the nonheme iron dioxygenase DdaC on the substrate *N*_β_FmmDAP tethered to the carrier protein T as part of the dapdiamide biosynthesis. The carrier protein T is highlighted with the pink box.

During the antibiotics biosynthesis chain of reactions to form dapdiamide, there is one specific step catalyzed by the enzyme DdaC, where a double bond epoxidation reaction is performed by an αKG‐dependent nonheme iron dioxygenase, see Scheme 1.^[^ ^7^, ^8^ ^]^ Interestingly, the DdaC precursor enzyme, namely DdaD, links the substrate *N*_β_‐fumaramoyl‐nonproteinogenic amino acid 2,3‐diaminopropionate (*N*_β_FmmDAP) to a carrier protein through a thioester linkage. The use of protein carriers that latch onto the surface of the protein and penetrate its terminus into the protein where it is activated has been observed for a number of enzymes. For instance, the αKG‐dependent nonheme iron halogenases often utilize a protein carrier for regio‐ and stereoselective aliphatic halogenation reactions.^[^ ^9^ ^]^ The tethered substrate generated from DdaD, thereafter, is activated by the DdaC enzyme using dioxygen and αKG on an iron(II) center to give the epoxide product *N*_β_EpSmDAP (Scheme 1). The experimental studies failed to characterize the stereochemistry of the product, and in addition, it is not clear why the substrate is tethered to a carrier protein and whether free substrate would react to form the same products. Therefore, to understand the effect of the use of a tethered substrate on double bond epoxidation reactions, we decided to perform a computational study on the mechanism of DdaC using two models, one with a tethered substrate and one with a nontethered substrate. The work presented here shows that the tethered substrate is better positioned in the active site, binds tighter, and reacts to give a regio‐ and stereoselective reaction mechanism to form the *trans*‐epoxide. By contrast, a nontethered substrate lacks hydrogen bonding interactions with the protein in the active site and hence is not positioned well for optimal catalysis.

## 2 Results and Discussion

### 2.1 Enzyme Structure Setup and MD Simulations

**Figure 1:**
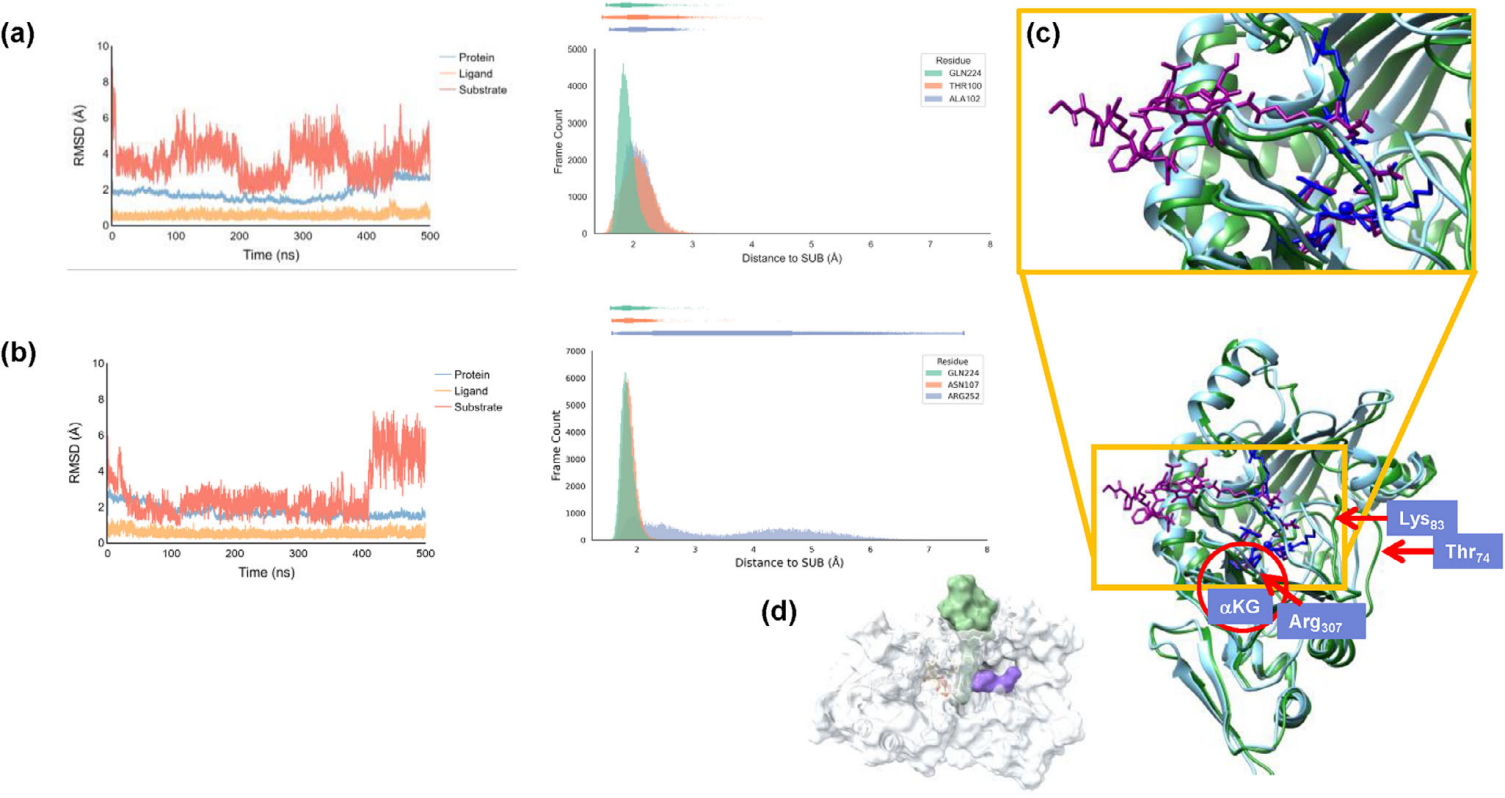
Results of the 500 ns MD simulations performed on the DdaC models with tethered and nontethered substrate. a) RMSD and distance plots for the MD simulation for the system with tethered substrate. b) RMSD and distance plots for the MD simulation for the system with a nontethered substrate. c) Overlay of the average structures obtained from the MD simulations on DdaC for the model with tethered substrate (atoms in dark purple and chains in dark green color) versus the model with nontethered substrate (atoms in dark blue and chains in light blue color). d) Surface drawing highlighting the substrate binding position in the average structure from the MD simulations with tethered (in green) and nontethered (in purple) substrate.

Using Alphafold2, we created a 3D structure of the DdaC protein and inserted iron(II) and αKG based on a structural comparison with the 1GQW protein databank (pdb) file; see Methods for details.^[^ ^10^, ^11^ ^]^ The iron(II)/αKG center was manually converted into an iron(III)‐superoxo/αKG active site by binding dioxygen to iron at a distance of 1.86 Å trans to His_298_. Thereafter, the HDock software package was used to dock tethered and nontethered substrate into the protein structure, and the most stable binding conformation was selected.^[^ ^12^ ^]^ After the addition of hydrogen atoms and solvent (water), a heating and equilibration protocol was performed for the enzyme models with tethered and nontethered substrate (see Methods). Thereafter, a 500 ns molecular dynamics (MD) simulation was run for both models, and the key results are shown in Figure 1, while further analysis of the data is given in the Supporting Information. Both MD simulations equilibrate rapidly (within a couple of ns), as seen from the root‐mean‐square‐deviation (RMSD) plots for the two enzyme structures (Figure 1a, b), whereby the RMSD is recorded for the substrate, the metal and its first coordination sphere, and the protein. In particular, the RMSD profile for the protein chain (in blue) and the metal with its first‐coordination sphere (labeled as ligand in Figure 1a, 1b) give very low RMSD values throughout the complete MD simulations, and hence the protein and metal binding site in both enzyme models are highly rigid and show little movement and dynamics during the MD simulations. The substrate shows slightly larger RMSD values, particularly for the tethered substrate, probably because the tethered substrate contains more atoms than the nontethered substrate. Note that the substrate RMSD profiles and values only compare the substrate atoms with each other and do not include the substrate‐protein interactions. Nevertheless, both substrate RMSD plots show equilibration of the structures happening with low overall RMSD values below 6 Å. Furthermore, a plot of the root‐mean‐square fluctuation (Supporting Information Figure S7) for both MD runs shows similar fluctuation patterns for the two systems hence, the overall structure of the two proteins does not seem to have been affected dramatically by the tether or its absence.

To gain insight into the substrate‐protein interactions and the substrate‐positioning and dynamics during the two MD simulations, overlays of a range of snapshots were created from structures taken after each 50 ns step. These overlays highlight little movements in the protein chain and cofactor binding site during each of the MD simulations (Supporting Information Figures S3 and S4). This is further confirmed by analyzing the dynamics B‐factors (Supporting Information Figures S5 and S6) that show areas of little degree of dynamics around the substrate and cofactor binding sites, whereas only significant movement is seen in areas far away from the metal center. By contrast, the substrate positioning in the active site changes dramatically for the nontethered substrate, whereas it is more constrained in the system with the tethered substrate (Supporting Information Figures S3 and S4).

Based on the average structures from the MD simulations with tethered and nontethered substrate, we created an overlay of the two enzyme structures as shown in Figure 1c. In general, the protein tertiary structure is well aligned between the two structures, particularly the areas representing the αKG binding pocket in the active site. The red circle in Figure 1c highlights the αKG group and its direct environment, which shows matching chains and protein residues for the two enzyme models. This is not surprising, as many nonheme iron and αKG‐dependent dioxygenases bind iron in a facial orientation linked to two histidine side chains and the carboxylate group of a Glu or Asp residue.^[^ ^11b^ ^]^ In addition, these enzymes have a conserved αKG‐binding pocket and a local environment that holds αKG in the equatorial plane (orthogonal to the oxo and His_298_ groups) and bridged between the iron(II) and an Arg side chain of the αKG‐binding loop.^[^ ^13^ ^]^ In DdaC the αKG forms a salt bridge with the side chain of Arg_307_ as highlighted in Figure 1c. The overlay between the average structure of the two MD runs shows that there are some obvious differences in substrate binding orientation for the tethered and nontethered structures. In particular, the protein carrier of the tethered substrate is latched onto the substrate entrance channel cavity on the surface and interacts with amino acid residues on the surface. These interactions affect the helices that surround the substrate binding pocket, and as a result, the loop inside the substrate binding pocket from Thr_74_ to Lys_83_ (highlighted with red arrows in Figure 1c) has moved inwards in the protein structure with the nontethered substrate, and the Ser_79_ residue now forms a direct hydrogen bond with the amide terminus of the substrate. Therefore, the protein carrier changes the fold of the protein slightly and affects the size and shape of the substrate binding pocket in such a way that the substrate is oriented closer to the metal cofactor. By contrast, the nontethered substrate has a different binding position as compared to the structure with the tethered substrate, and the double bond is at a larger distance from the iron center, which may affect catalysis.

To gain further insight into substrate binding and positioning and its interactions with the protein environment, we analyzed the snapshots from the MD simulations in further detail. In particular, the interactions of the substrate with the protein were measured through distance distributions between the substrate and hydrogen bonding donor and acceptor groups from the protein, and we show these as distance plots in Figure 1a, b. The tethered substrate binds tightly and forms strong hydrogen bonding interactions with several protein residues. Thus, the amide group of the Gln_224_ residue forms a hydrogen bonding interaction with the amide group of the tethered substrate in the majority of the snapshots, and the distance distribution shows this group to be within 2.5 Å in more than 90% of all snapshots with a peak maximum at a distance of 1.8 Å. The terminal amide group of the substrate also interacts with the amide side chain of the Asn_107_ residue as well as the Arg_82_ side chain that bridges the two carboxylate groups of succinate/αKG and Glu_106_. Finally, the Thr_93_ alcohol group accepts a hydrogen bond from the NH_3_^+^ group of the tethered substrate. The Thr_100_ side chain points to the outside of the substrate binding pocket and forms hydrogen bonding interactions with surface‐bound groups of the substrate, including an amide group of an Asn residue and an imidazole group of a His residue at the tethered tail. These interactions are strong and stay in place during most snapshots of the MD simulation, as seen from the distance plot (Figure 1 and Supporting Information Figure S16).

The nontethered substrate also interacts with the Gln_224_ side chain of the protein in most snapshots with a maximum in the distance distribution just under 2 Å. In addition, the nontethered substrate interacts with the Ser_79_ side chain in most of the snapshots, which is part of the inward‐bound loop from Thr_74_ to Lys_83_ that has shifted as compared to the system with the tethered substrate. This residue pulls the substrate away from the iron center and triggers differences in substrate binding position for the tethered versus nontethered model systems. Obviously, an interaction of the amide terminus of the substrate with Ser_79_ will put the substrate too far into the substrate binding pocket and may make substrate epoxidation challenging in favor of an alternative catalytic reaction. The same is true for the Asn_232_ residue that gives interaction patterns with the nontethered substrate even though it is located at distances far from the iron center. By contrast, the Asn_232_ residue does not strongly interact with the tethered substrate. Interestingly, the nontethered substrate does not interact strongly with the Thr_93_ alcohol side chain, and few snapshots show hydrogen bonding interactions. Therefore, the tethered substrate is latched in a different orientation over the nontethered substrate, which may affect its interaction with the metal center and its reactivity pattern.

**Figure 2:**
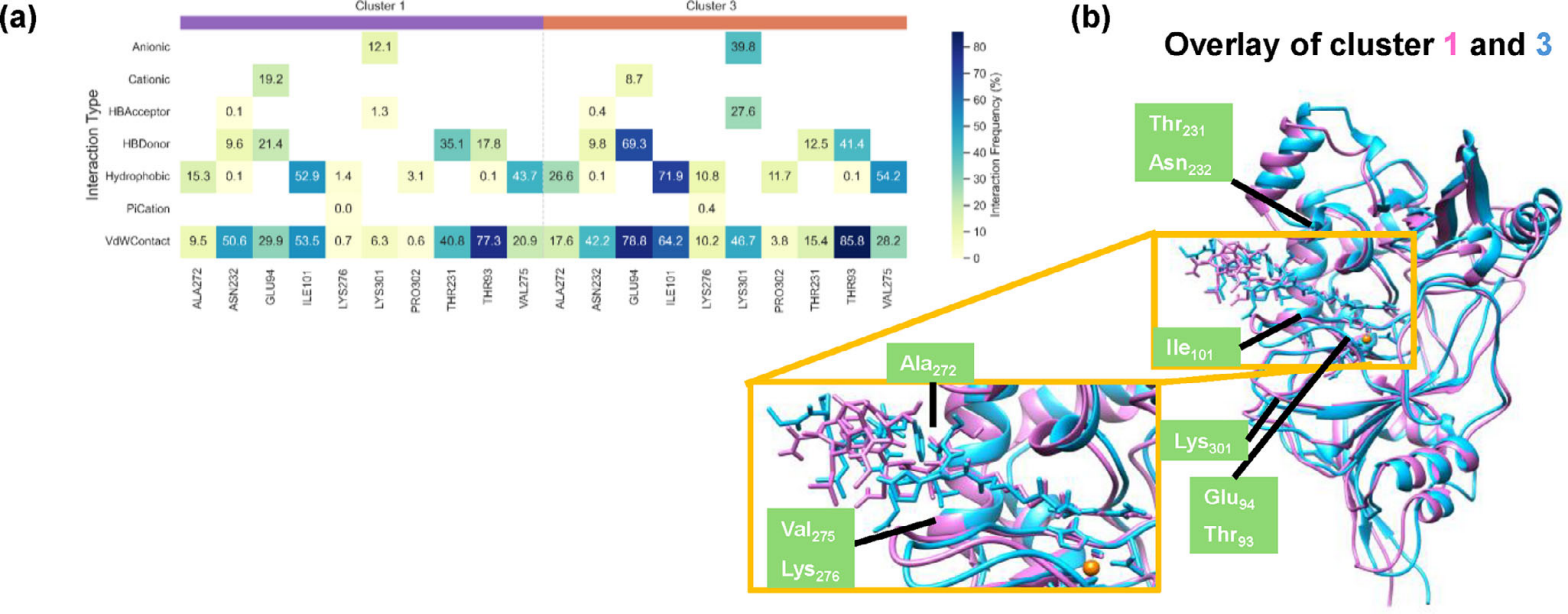
Analysis of the 500 ns MD simulation for DdaC with bound tethered substrate based on clusters 1 and 3 obtained with the agglomerative‐clustering technique. a) Interaction details between substrate and protein residues and the classification of the interaction type. b) Overlay of the cluster 1 and cluster 3 optimal structures from agglomerative‐clustering analysis of the MD simulation structures.

We then used the agglomerative‐clustering technique and analyzed the structures obtained from the 500 ns MD simulation with the tethered substrate and divided those into four cluster types, designated as cluster 0, 1, 2, and 3.^[^ ^14^ ^]^ Cluster 0 from agglomerative clustering is dominant in the first 100 ns of the MD simulation, whereas in the next 100 ns cluster 2 is dominant; see Supporting Information Figure S20. In the final 300 ns of the MD simulation, the structures flip between the agglomerative cluster models 1 and 3 and hence these will be the important equilibrated structures that will determine catalysis. In Figure 2 we show the analysis of the cluster 1 and cluster 3 structures obtained with the agglomerative‐clustering technique on the MD simulation on the tethered substrate. The two clusters have similar overall fold but bind the tethered substrate in a slightly different orientation and particularly have the part of the substrate that is latched on the protein surface flipped. As the substrate tail enters the protein under the same angle, its terminus with the C═C double bond is positioned in the same place in the active site that is near the iron(II) center. In both structures there are hydrogen bonding interactions of the terminal amide group with the side chain of Gln_224_. To gain insight into the interactions between the substrate and protein, we analyzed the substrate‐residue interactions and characterized these as hydrophobic, hydrogen bonding (acceptor or donor), van der Waals, charged or π‐stacking interactions and show these interactions for clusters 1 and 3 in Figure 2a, while the main interactions for tethered and nontethered substrate structures are shown in Figures S18 and S23 (Supporting Information). Residues located on the surface, for example, Ala_272_, Val_275_, and Lys_276_ interact, in a similar way with substrate in clusters 1 and 3 and mostly through hydrophobic and van de Waals contacts. By contrast, the residues Ile_101_, Thr_231_, and Asn_232_ are on the edge of the substrate entrance channel and are strongly affected by the orientation of the substrate onto the protein surface. Indeed, in cluster model 1, the Ile_101_ group has considerably fewer hydrophobic and van der Waals contacts than in model 3, whereas the opposite is seen for Thr_231_, which acts as a hydrogen bond donor and van der Waals contact in model 1, while its contribution is negligible in model 3. Finally, the Glu_94_ residue acts as a hydrogen bond donor and van der Waals contact in cluster 3, while these contributions are significantly reduced in cluster 1, where instead it interacts with a cationic group (NH_3_^+^ group of the substrate).

In agreement with the distance plot for the tethered substrate, we find van der Waals contacts and hydrogen bonding donation to the substrate from the Gln_224_ residue to the substrate throughout the MD simulation, while the Asn_107_ residue provides van der Waals contacts throughout the MD simulation and additional hydrogen bonding interactions with the substrate, although these count for only 60% of the snapshots (Figure S18, Supporting Information). The Thr_100_ side chain provides hydrogen bonding interactions to the substrate in a majority of the cases, while the interactions from Thr_93_ are mostly of the van der Waals type and those from Tyr_95_ are mainly hydrophobic. The substrate‐residue interactions obtained for the MD simulation of the nontethered substrate show dramatic differences from the one with the tethered substrate (Supporting Information Figure S23). Thus, the van der Waals and hydrogen bonding donation interactions of Gln_224_ are still observed for the nontethered substrate. However, interactions of the substrate with Asn_107_ are not seen anymore and instead strong van der Waals and hydrogen bonding interactions with the Ser_79_ side chain are obtained. This matches the overlay of the two average structures shown in Figure 1c, where the chain that includes the Ser_79_ residue was seen to have moved inwards with the nontethered substrate. In addition, the interactions of Thr_93_, Tyr_95_, and Thr_100_ with the nontethered substrate are virtually missing. Consequently, the nontethered substrate binds in a different position and orientation as compared to the tethered substrate, which may affect the enzyme catalysis reaction.

Finally, the binding free energy of the tethered substrate in cluster 1 and cluster 3 of the MD simulation was calculated. Not surprisingly, the strongest contribution to the binding free energy comes from electrostatic interactions of the substrate with the side chains of Glu_94_ and Lys_301_, and to a lesser extent with, Lys_276_ (Supporting Information Tables S5 and S6). These three residues are charged and located on the surface of the protein and will interact with the protein carrier that is latched on the surface, although no direct hydrogen bonding interactions are seen in any of the snapshots. Furthermore, the Glu_94_, Lys_276_, and Lys_301_ residues also show a large contribution to the polar solvation of the protein. The overall binding energy of the tethered substrate in cluster 3 is larger than that in cluster 1 (Supporting Information Table S4) and follows the trend of the whole protein the best. As such, the MD cluster 3 structure was used to create a quantum chemical model for calculations on the overall reaction mechanism.

### 2.2 QM Cluster Set‐up and Substrate Activation Mechanism

**Figure 3:**
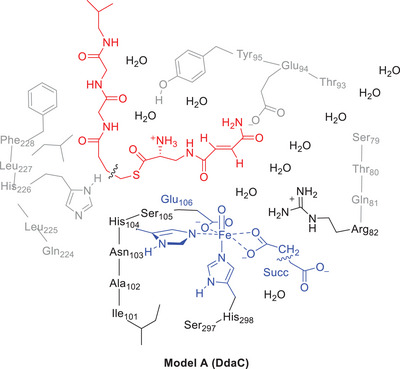
Cluster model **A** of tethered substrate‐bound DdaC as investigated in this work. Model **B** has the substrate truncated at the wiggly line and the Glu_94_, His_226_, and Ser_297_ residues truncated to Gly.

Based on the agglomerative‐clustering approach of the two structures obtained from the MD simulations with tethered and nontethered substrate, we created quantum chemical cluster models **A** and **B**, respectively, and studied the substrate epoxidation reaction. These cluster models include the metal and substrate with their first‐ and second‐coordination spheres and were designed using previously proposed methods.^[^ ^15^ ^]^ As nonheme iron dioxygenases use iron(II), O_2_ and αKG to form an iron(IV)‐oxo active species for catalysis, both models were given a central iron(IV)‐oxo species with bound succinate.^[^ ^1^, ^2^, ^3^ ^]^ The mechanism for the conversion of iron(III)‐superoxo to iron(IV)‐oxo was studied previously for various nonheme iron dioxygenases, and in general it is highly exothermic with a small barrier for dioxygen attack on the α‐keto‐position of αKG.^[^ ^16^ ^]^ Model **A** is a large model with a tethered substrate as shown in Figure 3 and contains the iron(IV)‐oxo species bound to the His_104_, Asp_106_, and His_298_ side chains in a facial orientation and with succinate abbreviated as acetate in the equatorial plane. On the axial site the dimer Ser_297_‐His_298_ was included. Part of the protein environment was selected in the model, namely the chains Ser_79_‐Thr_80_‐Gln_81_‐Arg_82_, Thr_93_‐Glu_94_‐Tyr_95_, Ile_101_‐Ala_102_‐Asn_103_‐His_104_‐Ser_105_‐Glu_106_, and Gln_224_‐Leu_225_‐His_226_‐Leu_227_‐Phe_228_ with the residues Ser_105_ and Leu_225_ truncated to a Gly residue. In addition, the model contained the first 64 atoms of the substrate, including three peptide bonds beyond the thioester group. Ten water molecules from the MD snapshot were included to obtain a model with tethered substrate (Model **A**) of 403 atoms and charge *Q* = +1. The system was calculated in the lowest singlet, triplet, and quintet spin states. We also calculated a smaller model **B** that represents the DdaC model with the nontethered substrate, whereby the substrate was truncated after the thioester bond and, in addition, the Glu_94_, His_226_, and Ser_297_ residues were replaced by Gly. Model **B** had 342 atoms and an overall charge + 2. No constraints were used in either model **A** or **B**.

**Figure 4:**
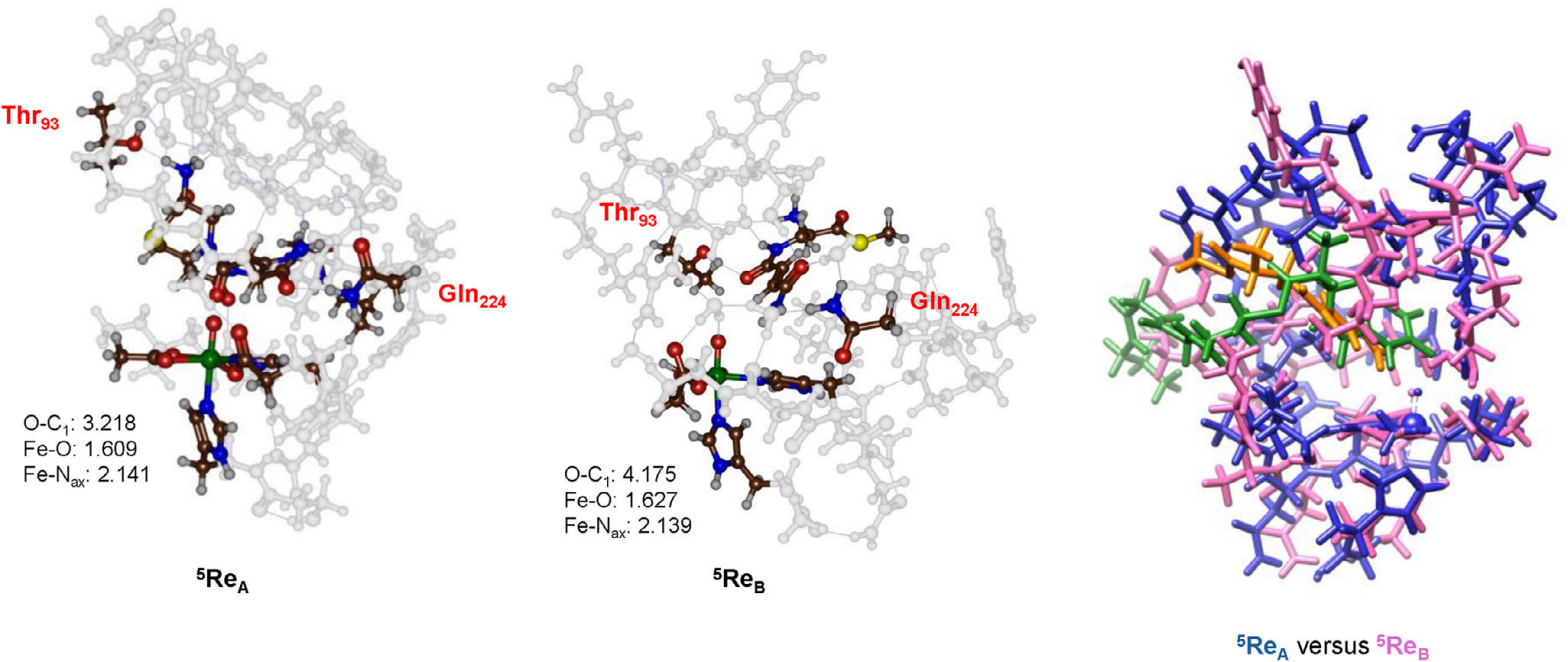
Left: UB3LYP/BS1 optimized geometries of ^5^ **Re**_A_ and ^5^ **Re**_B_ with bond lengths in Å. Right: Overlay of the ^5^ **Re**_A_ (in blue with substrate in green) and ^5^ **Re**_B_ (in plum with substrate in orange) optimized geometries whereby the metal and its first‐coordination sphere were matched between the two structures.

Subsequently, a full geometry optimization of ^5^ **Re**_A_ and ^5^ **Re**_B_ was performed, and the optimized structures are shown in Figure 4. The singlet and triplet spin state structures for model **A** (^1,3^ **Re**_A_) were also calculated and found to be higher in free energy than ^5^ **Re**_A_ by 15.7 and 7.0 kcal mol^−1^. As such, the quintet spin state is the ground state, which matches experimental work on the iron(IV)‐oxo species of the nonheme iron dioxygenases taurine/αKG‐dependent dioxygenase, prolyl‐4‐hydroxylase, and the viomycin synthesis enzyme C that all were characterized with electron spin paramagnetic resonance measurements as a quintet spin ground state.^[^ ^17^ ^]^ The quintet spin reactant structure has four unpaired electrons that are mostly metal‐based with orbital occupation designated π*_xy_^1^ π*_xz_^1^ π*_yz_^1^ σ*_x_2_−y_2^1^. The three π* orbitals contain metal 3d_xy_, 3d_xz_, and 3d_yz_ atom orbital contributions (the *z*‐axis is along the Fe − O bond), and these orbitals contain interactions mostly with the 2p orbitals on the oxygen atom. The σ*_x_2_−y_2 orbital is in the equatorial plane and forms antibonding interactions between the metal 3d_x_2_−y_2 orbital and 2p orbitals on the carboxylate group of Glu_106_, the carboxylate group of succinate, and the nitrogen atom of His_104_. Consequently, both ^5^ **Re**_A_ and ^5^ **Re**_B_ show a spin density of 3.75 on the FeO group. The axial histidine (His_298_) binds the metal with an Fe − N_ax_ bond of 2.141 Å in ^5^ **Re**_A_ and 2.139 Å in ^5^ **Re**_B_; hence, the two structures have the axial His in virtually the same position. The distal oxo group is at a distance of 1.609 Å from the metal in ^5^ **Re**_A_, while it is 1.627 Å in ^5^ **Re**_B_. Clearly, the differences in substrate positioning and orientation affect the Fe − O distance slightly. Nevertheless, both Fe − O distances are in good agreement with extended X‐ray absorption fine structure measurements on taurine/αKG‐dependent dioxygenase that obtained a value of 1.61 Å.^[^ ^18^ ^]^

The DFT‐optimized structures ^5^ **Re**_A_ and ^5^ **Re**_B_ in both cases have the substrate interacting with the alcohol group of Thr_93_ and the amide group of Gln_224_. The alcohol group of Thr_93_ forms a direct hydrogen bonding interaction with the NH_3_^+^ group of the tethered substrate at a distance of 1.693 Å, while it forms a hydrogen bond with the carbonyl group of the substrate that is next to the double bond in the nontethered substrate model. In the model with the tethered substrate (^5^ **Re**_A_), however, the substrate‐protein interactions include a double hydrogen bond between the terminal amide group of the substrate with the amide group of Gln_224_ (with hydrogen bond lengths of 1.901 and 1.960 Å), whereas in ^5^ **Re**_B_ two water molecules bridge the NH_2_ group of Gln_224_ with the oxygen and NH_2_ groups of the substrate amide group. This implies that the tethered substrate in model **A** is positioned differently in the active site as compared to the nontethered substrate in model **B**. Indeed, the O − C distances between the iron(IV)‐oxo species and the terminal carbon atom of the double bond of the substrate is 3.218 Å in ^5^ **Re**_A_, while it is 4.175 Å in ^5^ **Re**_B_. Therefore, the model with the tethered substrate has the double bond positioned closer to the iron(IV)‐oxo species than the nontethered substrate. It appears that the carrier protein prevents the substrate from entering too deep into the substrate binding pocket, as seen for the free substrate model. To gain further insight into substrate positioning in models **A** and **B**, we created an overlay of the two structures, where we matched the iron(IV)‐oxo and the metal first‐coordination sphere of the two structures; see the right‐hand‐side of Figure 4. As can be seen from the overlay of the two structures, the tethered substrate in model **A** is bound in a different conformation and orientation as compared to the nontethered substrate in model **B**. This influences not only the substrate binding and orientation but also the active site structure resulting in different secondary structures and amino acid interactions. These DFT results match the overlays from the average structure of the MD simulations well and highlight that the protein carrier disrupts the protein fold and the substrate binding pocket. In addition, we created an overlay of ^5^ **Re**_A_ and ^5^ **Re**_B_ with the average structure from the MD simulation the model was created from (Supporting Information Figure S31). Little changes are seen after geometry optimization of the QM cluster models, and in general the protein chains are found in the same position in the cluster model and the MD snapshot for both systems.

**Figure 5:**
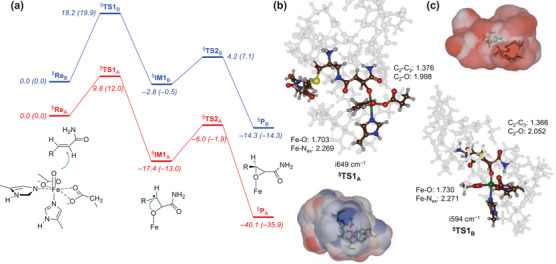
a) UB3LYP‐GD3/BS2//UB3LYP /BS1 calculated the potential energy profile (values in kcal mol^−1^) for C═C bond epoxidation of the substrate in ^5^ **Re**_A_ and ^5^ **Re**_B_. Enthalpies (ΔE + ZPE) are out of parenthesis, and free energy values are in parenthesis. b) and c) Optimized C − O bond formation transition states with bond lengths in Å and the imaginary frequency in cm^−1^ for models **A** and **B**. Electrostatic potential surfaces are shown for the transition state structures with negative regions in blue and positive regions in red.

Next, we calculated the mechanism of C═C bond epoxidation of the QM cluster models with tethered and nontethered substrates ^5^ **Re**_A_ and ^5^ **Re**_B_, and the free energy landscapes are shown in Figure 5. For both systems, the reactions are stepwise with an initial electrophilic addition transition state **TS1** to form the C − O bond to give a radical intermediate **IM1** and a subsequent ring‐closure transition state **TS2** to obtain the epoxide product **P**. The initial C − O bond formation barrier via ^5^ **TS1** is rate‐determining for the models with tethered and nontethered substrate; hence, the tether does not affect the epoxidation reaction mechanism. Calculated enthalpies and free energies for the landscape are shown in Figure 5a, and they generally follow the same trend, consequently, the inclusion of zero‐point energy, thermal corrections and entropy does not influence the reaction patterns. Previous computational studies on C═C epoxidation by iron(IV)‐oxo complexes of heme and nonheme complexes also found a stepwise and radical reaction mechanism with a rate‐determining electrophilic attack of the oxo on the double bond of the substrate.^[^ ^19^ ^]^ The model with the tethered substrate has a ^5^ **TS1**_A_ barrier of only 12.0 kcal mol^−1^ with respect to the reactants complex, whereas the nontethered substrate encounters a barrier of 19.9 kcal mol^−1^. This implies that the tethered substrate is better positioned in the active site for substrate epoxidation guided by the second coordination sphere of the substrate binding pocket. Previously, using a minimal cluster model of a nonheme iron dioxygenase, a barrier of 4.8 kcal mol^−1^ was calculated for propene activation, whereas an octahedral coordinated biomimetic iron(IV)‐oxo species gave a barrier of 17.9 kcal mol^−1^.^[^ ^20^ ^]^ Unfortunately, there are no experimental rate constants for the epoxidation reaction catalyzed by DdaC, but for the hydrogen atom abstraction from taurine by taurine/αKG‐dependent dioxygenase (TauD), a rate constant of 13 s^−1^ at 5 °C was obtained, which would correspond to an activation free energy of ΔG^‡^ = 14.8 kcal mol^−1^ at room temperature.^[^ ^21^ ^]^ Our calculated free energy of activation, therefore, is in line with the value for hydrogen atom abstraction reactions by nonheme iron dioxygenases from the literature. Due to the large energy difference between the quintet spin state and other spin states in the reactant conformation, the epoxidation barriers on the singlet and triplet spin states are also much higher in free energy than the one located for the quintet spin state; see Supporting Information.

The optimized transition state structures ^5^ **TS1**_A_ and ^5^ **TS1**_B_ are shown in Figure 5b. Both have an imaginary frequency that represents the C − O stretch vibration with a magnitude of i649 cm^−1^ (model **A**) and i594 cm^−1^ (model **B**). These values are relatively large for epoxidation barriers, as previous studies located them in the range of i200 – i500 cm^−1^.^[^ ^19^, ^20^ ^]^ The Fe − O distance in the complex has elongated from 1.609/1.627 Å in ^5^ **Re**_A_/^5^ **Re**_B_ to 1.703 Å in ^5^ **TS1**_A_ and 1.730 Å in ^5^ **TS1**_B_, while the Fe − N_ax_ distances have elongated by about 0.13 Å for both models. Not surprisingly, the C − C bond goes from 1.343/1.340 Å in ^5^ **Re**_A_/^5^ **Re**_B_ to 1.376 Å (^5^ **TS1**_A_) and 1.366 Å (^5^ **TS1**_B_). Electronically, the two transition states (as well as the two reactant complexes) are very similar in electronic configuration and orbital occupation. In both cases an electron transfer from the double bond into the σ*_z_2 orbital takes place that leads to a radical intermediate with π*_xy_^1^ π*_xz_^1^ π*_yz_^1^ σ*_x_2_−y_2^1^ σ*_z_2^1^ π_Sub_^1^ configuration, whereby the π_Sub_ orbital is a substrate radical (on atom C_3_ mostly) with a down‐spin electron. This is common in hydrogen atom abstraction intermediates calculated for nonheme iron enzymes and biomimetic models.^[^ ^16^, ^22^, ^23^ ^]^ The two transition states have similar spin densities and charges (Table S9, Supporting Information), and hence the electronic configuration is not the reason for the difference in energy between the two structures. As the ^5^ **TS1**_A_ and ^5^ **TS1**_B_ structures are very similar and proceed via the same electron transfer pathways with similar electronic configurations, we decided to analyze the electrostatic potentials (ESP) around the substrate with the Multiwfn software package.^[^ ^24^ ^]^ The ESP surfaces of ^5^ **TS1**_A_ and ^5^ **TS1**_B_ are shown in Figure 5a, b. As can be seen, the ^5^ **TS1**_A_ structure gives large areas with negative electrostatic potential, whereas the ^5^ **TS1**_B_ structure is dominated by regions of positive electrostatic potential. Consequently, the two transition state structures experience different dipole moments and protein electrostatic and electric field perturbations that influence their stability and relative energies. The local charge distributions, therefore, will stabilize the transition state for the tethered substrate better and thereby lower the free energy of activation for C − O bond formation. Clearly, a substrate surrounded by a positive electrostatic potential has a high barrier for C═C activation, whereas a substrate in a more negative electrostatic potential is stabilized.

**Figure 6:**
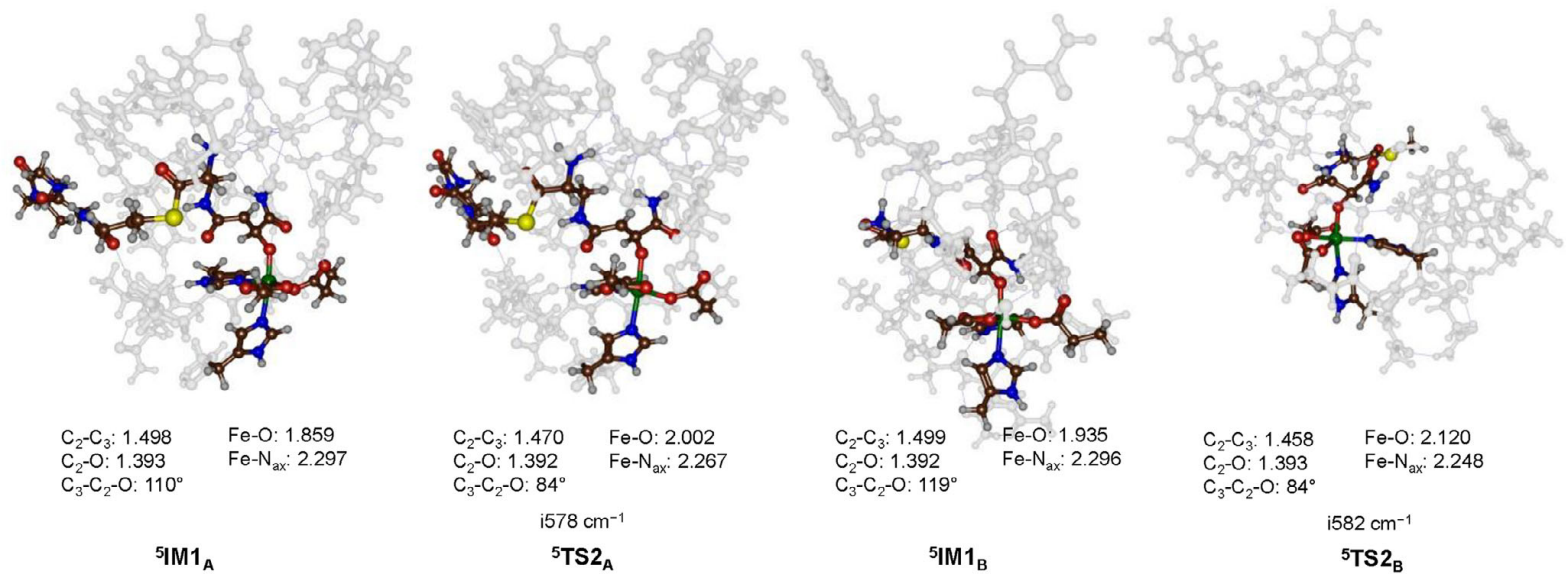
Optimized epoxidation ring‐closure transition states with bond lengths in Å, the angle in degrees, and the imaginary frequency in cm^−1^.

From the transition states the systems relax to a radical intermediate (**IM1**_A_ and **IM1**_B_), of which the structures are shown in Figure 6. In these structures there is a full radical on the substrate with down‐spin, while the metal d‐block contains five singly occupied orbitals with up‐spin. The occupation of the σ*_z_2 orbital with one electron results in additional antibonding character along the Fe − O bond, which is elongated from 1.703 Å in ^5^ **TS1**_A_ to 1.859 Å in ^5^ **IM1**_A_, and from 1.730 Å in ^5^ **TS1**_B_ to 1.935 Å in ^5^ **IM1**_B_. At the same time the C − O distances shorten and the C_1 _− C_2_ distances elongate, which implies that the latter bond is reduced from a double bond to a single bond. From the radical intermediates, a ring‐closure transition state **TS2** leads to the epoxide product complexes. The barriers are substantial for both systems, with a ΔG = 7.6 kcal mol^−1^ for the nontethered system and 11.1 kcal mol^−1^ for the tethered substrate with respect to the radical intermediates. Clearly, the constraints of the tethered substrate and its large number of hydrogen bonding interactions with the protein make the ring closure more difficult and hence encounter a higher barrier than that observed for the model with the nontethered substrate. Optimized ring‐closure transition state structures ^5^ **TS2**_A_ and ^5^ **TS2**_B_ are shown in Figure 6. They have almost identical imaginary frequency of around i580 cm^−1^ for the C_3 _− C_2 _− O bending motion. The C_2 _− C_3_ distance has elongated to 1.470 Å in ^5^ **TS2**_A_ and to 1.458 Å in ^5^ **TS2**_B_, while the C_2 _− O distances are 1.392 and 1.393 Å, respectively. The Fe − O bond has further elongated to 2.002 Å in ^5^ **TS2**_A_ and 2.120 Å in ^5^ **TS2**_B_. Overall, the ring‐closure transition states are not dramatically different from each other and are structurally in good agreement with those calculated previously for analogous iron(IV)‐oxo systems.^[^ ^19^, ^20^ ^]^

## 3 Conclusion

In this work a computational study on the double bond epoxidation performed by the nonheme iron dioxygenase DdaC is presented. Two enzyme models were created with the substrate either tethered to a carrier protein or as a nontethered (free) substrate, and MD simulations were run for both models. The MD analysis shows that the tethered substrate is positioned tightly in the substrate binding pocket with the double bond close to the metal center and is locked in hydrogen bonding interactions with, for instance, Gln_224_. By contrast, the model with the nontethered substrate positions the substrate deeper into the substrate binding pocket with the double bond at a larger distance from the metal center but also leads to changes in the protein fold and structure. In particular, the carrier protein disrupts the protein fold and alters the shape and size of the substrate binding pocket through its hydrogen bonding interactions on the surface as well as with the chain that enters the substrate entrance channel. Subsequently, QM cluster models were created of the metal and substrate with their first‐ and second‐coordination sphere included and either a nontethered or tethered substrate. Both models have the same ground state and electronic configuration and overall iron(IV)‐oxo structure. The substrate epoxidation reaction is stepwise via a radical intermediate. The QM cluster calculations on the tethered model give substantially lower epoxidation barriers than those with the nontethered substrate. Analysis of the structure and electronic properties of the transition state shows that the nontethered substrate is located in an area of high positive electrostatic potential, while the tethered substrate is surrounded by more negative electrostatic potential, which influences the reaction energetics and brings down the barriers for the tethered substrate. Moreover, the tethered substrate reacts in the enzyme through a stereoselective reaction mechanism that forms the *trans*‐epoxide. The work shows that the active oxidant of DdaC is a high‐valent iron(IV)‐oxo species that reacts with the double bond of the substrate through an epoxidation reaction. Our study shows the importance of the carrier protein in triggering a selective reaction mechanism by a nonheme iron enzyme that may be relevant to other enzymes that use a tethered substrate.

## 4 Experimental Section

### Enzyme setup and MD simulation

Since there is no available crystal structure of the DdaC enzyme, we used the Alphafold software package to predict a 3D structure.^[^ ^10d^ ^]^ The amino acid sequences of DdaD and DdaC were obtained with FASTA, that is, the GenBank file ADN39483.1 for DdaD with 578 amino acids and the ADN39482.1 file for DdaC with 329 amino acids.^[^ ^10a^ ^]^ Subsequently, the online tool of AlphaFold2 in combination with the MMseqs2 method generated predicted structures for both proteins.^[^ ^10^ ^]^ The active site iron(II) and αKG groups were inserted through an analogy with the crystal structure coordinates of taurine/αKG‐dependent dioxygenase (1GQW pdb) and manually placed in their coordination environment using Pymol.^[^ ^11^ ^]^ Hydrogen atoms were added in Chimera under pH 7 conditions,^[^ ^25^ ^]^ whereby all Lys and Arg residues were in their protonated states, while all Asp and Glu residues were set in the deprotonated conformations. The substrate *N*_β_FmmDAP is covalently tethered to the T‐domain of the DdaD protein (Scheme 1), and its coordinates were taken from the DdaD structure. Two full enzyme models were constructed, namely one with a tethered substrate with 11 amino acids beyond the thioester linkage (enzyme model **I**) and one where the substrate is truncated after the thioester bond with a methyl group (enzyme model **II**). The online HDOCK server tool^[^ ^12^ ^]^ was used to dock the two substrate geometries into the DdaC protein structure, which created 10 reasonable outcomes for each substrate, and the most stable docking pose with the highest score was selected for the next step.

The substrates were parametrized using the General Amber Force Field (GAFF), while the protein chain was parametrized with the AMBER FF19SB forcefield.^[^ ^26^ ^]^ The metal‐center‐parameter‐builder was used to obtain parameters for the metal and its first coordination sphere ligands.^[^ ^27^ ^]^ Both models were solvated in a box with OPC‐defined water molecules with 10 Å dimensions, and the system was neutralized by adding Na^+^ and Cl^−^ anions to the surface. Thereafter, an MD simulation was performed for 500 ns for enzyme models **I** and **II** in Gromacs.^[^ ^28^ ^]^ The general protocol of the MD runs started with a steepest descent energy minimization, followed by a two‐stage equilibration. First, the system was equilibrated for 1 ns in the NVT ensemble at 300 K using the velocity‐rescale thermostat. This was followed by equilibration in the NPT ensemble at 1 bar using the Berendsen barostat. Finally, 500 ns production simulations were conducted under the NPT ensemble, employing the Parrinello‐Rahman barostat for more accurate pressure coupling. Throughout all dynamic steps, a leap‐frog integrator was employed with a time step of 2 fs, and all bonds involving hydrogen atoms were constrained using the LINCS algorithm. Long‐range electrostatics were handled by Particle Mesh Ewald (PME) with a 1.2 nm cutoff, while van der Waals interactions were treated with a force‐switch function between 1.0 and 1.2 nm. Periodic boundary conditions were applied in all three dimensions. The individual snapshots of the MD simulations were analyzed for substrate‐residue interactions using ProLIF.^[^ ^29^ ^]^

Binding free energies of the substrate in the protein matrix were calculated with the MM‐PBSA module as implemented in Amber.^[^ ^30^, ^31^ ^]^ The adaptive Poisson‐Boltzmann solver software was utilized with a grid spacing of 0.5 Å and an ionic strength of 150 mM. The solvent and solute dielectric constants were taken as 78.5 and 1.0, respectively.

### DFT calculations

A series of density functional theory calculations on the enzymatic reaction mechanisms were performed using the Gaussian‐16 software package.^[^ ^32^ ^]^ In analogy to previous work from our group,^[^ ^33^ ^]^ the unrestricted hybrid B3LYP density functional method was applied for geometry optimizations, constrained geometry scans, and analytical frequencies.^[^ ^34^ ^]^ Geometry optimizations, analytical frequency calculations, and constraint geometry scans utilized the def2‐SVP basis set on all atoms (basis set BS1),^[^ ^35^ ^]^ while single points were followed at the UB3LYP‐GD3/def2‐TZVP level of theory (basis set BS2) to obtain more accurate energies.^[^ ^34^, ^36^ ^]^ Transition states were characterized from a single imaginary mode in the frequency file with a motion that corresponds with the reaction mechanism. Free energies were obtained from the Gaussian frequency files using unscaled vibrational frequencies at a temperature of 298 K. The broken‐symmetry approach was used to characterize open‐shell spin states. We have used these approaches in various previous projects, and the methods used here reproduced experimental product distributions and rate constants well.^[^ ^37^ ^]^ To test the reproducibility of the energetics obtained in this work, we recalculated the spin‐state energies of the reactant complexes through single‐point calculations with a range of alternative functionals and basis sets, that is, at the UB3LYP/BS2,^[^ ^34^, ^35^ ^]^ UB3LYP‐GD3/BS2,^[^ ^34^, ^35^, ^36^ ^]^ and UPBE1PBE‐GD3/BS2.^[^ ^34^, ^35^, ^38^, ^39^ ^]^ These calculations confirmed the energetics and spin state ordering; see Supporting Information.

## Conflict of Interest

The authors declare no conflict of interest.

## Supporting information



Supporting Information

## Data Availability

The data that support the findings of this study are available in the supplementary material of this article.

## References

[1] a) C. J. Schofield , Z. Zhang , Curr. Opin. Struc. Biol. 1999, 9, 722; 10.1016/s0959-440x(99)00036-6 10607676

[2] a) M. D. White , E. Flashman , Curr. Opin. Chem. Biol. 2016, 31, 126; 27015291 10.1016/j.cbpa.2016.02.017 PMC4879150

[3] a) R. P. Hausinger , Crit. Rev. Biochem. Mol. Biol. 2004, 39, 21; 15121720 10.1080/10409230490440541

[4] a) Y. Matsuda , I. Abe , Nat. Prod. Rep. 2016, 33, 26; 26497360 10.1039/c5np00090d

[5] a) C. Wang , W.‐c. Chang , Y. Guo , H. Huang , S. C. Peck , M. E. Pandelia , G. M. Lin , H.‐w. Liu , C. Krebs , J. M. Bollinger Jr. , Science 2013, 342, 991; 24114783 10.1126/science.1240373 PMC4160821

[6] a) W.‐c. Chang , J. Li , A. A. Cronican , Y. Guo , J. Am Chem. Soc. 2016, 138, 10390; 27442345 10.1021/jacs.6b05400

[7] M. A. Hollenhorst , S. B. Bumpus , M. L. Matthews , J. M. Bollinger Jr , N. L. Kelleher , C. T. Walsh , J. Am. Chem. Soc. 2010, 132, 15773. 20945916 10.1021/ja1072367 PMC2974046

[8] a) J. Dawlaty , X. Zhang , M. A. Fischbach , J. Clardy , J. Nat. Prod. 2010, 73, 441; 20041689 10.1021/np900685z PMC2846032

[9] a) M. L. Matthews , C. M. Krest , E. W. Barr , F. H. Vaillancourt , C. T. Walsh , M. T. Green , C. Krebs , J. M. Bollinger Jr. , Biochemistry 2009, 48, 4331; 19245217 10.1021/bi900109z PMC2684568

[10] a) P. Bryant , G. Pozzati , A. Elofsson , Nat. Commun. 2022, 13, 1265; 35273146 10.1038/s41467-022-28865-w PMC8913741

[11] a) J. M. Elkins , M. J. Ryle , I. J. Clifton , J. C. Dunning Hotopp , J. S. Lloyd , N. I. Burzlaff , J. E. Baldwin , R. P. Hausinger , P. L. Roach , Biochemistry 2002, 41, 5185; 11955067 10.1021/bi016014e

[12] Y. Yan , H. Tao , J. He , S. Y. Huang , Nat. Protoc. 2020, 15, 1829. 32269383 10.1038/s41596-020-0312-x

[13] H. S. Ali , R. H. Henchman , S. P. de Visser , Chem. Eur. J. 2021, 27, 1795. 32965733 10.1002/chem.202004019

[14] F. Pedregosa , G. Varoquaux , A. Gramfort , V. Michel , B. Thirion , O. Grisel , M. Blondel , P. Prettenhofer , R. Weiss , V. Dubourg , J. Vanderplas , A. Passos , D. Cournapeau , M. Brucher , M. Perrot , É. Duchesnay , J. Mach Learn. Res. 2011, 12, 2825.

[15] a) P. E. M. Siegbahn , M. R. A. Blomberg , Chem. Rev. 2010, 110, 7040; 20677732 10.1021/cr100070p

[16] a) T. Borowski , A. Bassan , P. E. M. Siegbahn , Chem. Eur. J. 2004, 10, 1031; 14978830 10.1002/chem.200305306

[17] a) J. C. Price , E. W. Barr , B. Tirupati , J. M. Bollinger Jr. , C. Krebs , Biochemistry 2003, 42, 7497; 12809506 10.1021/bi030011f

[18] D. Galonić Fujimori , E. W. Barr , M. L. Matthews , G. M. Koch , J. R. Yonce , C. T. Walsh , J. M. Bollinger Jr , C. Krebs , P. J. Riggs‐Gelasco , J. Am. Chem. Soc. 2007, 129, 13408. 17939667 10.1021/ja076454e

[19] a) C. Linde , B. Åkermark , P.‐O. Norrby , M. Svensson , J. Am. Chem. Soc. 1999, 121, 5083;

[20] a) S. P. de Visser , Angew. Chem. Int. Ed. 2006, 45, 1790; Angew. Chem. 2006, 118, 1822−1825;

[21] J. M. Bollinger Jr , J. C. Price , L. M. Hoffart , E. W. Barr , C. Krebs , Eur. J. Inorg. Chem. 2005, 21, 4245.

[22] a) S. P. de Visser , J. Am. Chem. Soc. 2006, 128, 9813; 16866538 10.1021/ja061581g

[23] a) D. Kumar , H. Hirao , L. Que Jr. , S. Shaik , J. Am. Chem. Soc. 2005, 127, 8026; 15926822 10.1021/ja0512428

[24] T. Lu , J. Chem. Phys. 2024, 161, 082503. 39189657 10.1063/5.0216272

[25] E. F. Pettersen , T. D. Goddard , C. C. Huang , G. S. Couch , D. M. Greenblatt , E. C. Meng , T. E. Ferrin , J. Comput. Chem. 2004, 25, 1605. 15264254 10.1002/jcc.20084

[26] a) J. Träg , D. Zahn , J. Mol. Model. 2019, 25, 39; 30659357 10.1007/s00894-018-3911-5

[27] P. Li , K. M. Merz Jr. , J. Chem. Inf. Model. 2016, 56, 599. 26913476 10.1021/acs.jcim.5b00674

[28] D. van Der Spoel , E. Lindahl , B. Hess , G. Groenhof , A. E. Mark , H. J. Berendsen , J. Comput. Chem. 2005, 26, 1701. 16211538 10.1002/jcc.20291

[29] C. Bouysset , S. Fiorucci , J. Cheminform. 2021, 13, No. 72. 10.1186/s13321-021-00548-6 PMC8466659 34563256

[30] J. Wang , R. M. Wolf , J. W. Caldwell , P. A. Kollman , D. A. Case , J. Comput. Chem. 2004, 25, 1157. 15116359 10.1002/jcc.20035

[31] <number>[31]</number > B. R. Miller 3rd , T. D. McGee Jr , J. M. Swails , N. Homeyer , H. Gohlke , A. E. Roitberg , J. Chem. Theory Comput. 2012, 8, 3314. 26605738 10.1021/ct300418h

[32] Gaussian‐16, Revision C.01, M. J. Frisch , G. W. Trucks , H. B. Schlegel , G. E. Scuseria , M. A. Robb , J. R. Cheeseman , G. Scalmani , V. Barone , G. A. Petersson , H. Nakatsuji , X. Li , M. Caricato , A. V. Marenich , J. Bloino , B. G. Janesko , R. Gomperts , B. Mennucci , H. P. Hratchian , J. V. Ortiz , A. F. Izmaylov , J. L. Sonnenberg , D. Williams‐Young , F. Ding , F. Lipparini , F. Egidi , J. Goings , B. Peng , A. Petrone , T. Henderson , D. Ranasinghe , V. G. Zakrzewski , J. Gao , N. Rega , G. Zheng , W. Liang , M. Hada , M. Ehara , K. Toyota , R. Fukuda , J. Hasegawa , M. Ishida , T. Nakajima , Y. Honda , O. Kitao , H. Nakai , T. Vreven , K. Throssell , J. A. Montgomery, Jr. , J. E. Peralta , F. Ogliaro , M. J. Bearpark , J. J. Heyd , E. N. Brothers , K. N. Kudin , V. N. Staroverov , T. A. Keith , R. Kobayashi , J. Normand , K. Raghavachari , A. P. Rendell , J. C. Burant , S. S. Iyengar , J. Tomasi , M. Cossi , J. M. Millam , M. Klene , C. Adamo , R. Cammi , J. W. Ochterski , R. L. Martin , K. Morokuma , O. Farkas , J. B. Foresman , D. J. Fox , Gaussian, Inc., Wallingford CT, 2019, Gaussian, Inc., Wallingford CT, 2010.

[33] a) A. Timmins , M. Saint‐André , S. P. de Visser , J. Am. Chem. Soc. 2017, 139, 9855; 28657747 10.1021/jacs.7b02839

[34] a) A. D. Becke , J. Chem. Phys. 1993, 98, 5648;

[35] F. Weigend , R. Ahlrichs , Phys. Chem. Chem. Phys. 2005, 7, 3297. 16240044 10.1039/b508541a

[36] J. A. Grimme , S. Ehrlich , H. Krieg , J. Chem. Phys. 2010, 132, 154104. 20423165 10.1063/1.3382344

[37] a) F. G. Cantú Reinhard , A. S. Faponle , S. P. de Visser , J. Phys. Chem. A 2016, 120, 9805; 27973805 10.1021/acs.jpca.6b09765

[38] C. Adamo , V. Barone , J. Chem. Phys. 1999, 110, 6158.

[39] a) P. J. Hay , W. R. Wadt , J. Chem. Phys. 1985, 82, 270;

