# Supplementary material for: Substrate Epoxidation Catalyzed by the Nonheme Iron Dioxygenase Dapdiamide Biosynthesis Enzyme C. Why Is the Substrate Tethered?
Source: Chemistry. 2025 Aug 21;31(53):e01610. doi: 10.1002/chem.202501610 (PMC12451424; doi:10.1002/chem.202501610)
Supplement: Supplementary file 1 — Supporting Information [file CHEM-31-e01610-s001.pdf]

Supporting Information

Substrate Epoxidation Catalyzed by the Nonheme Iron Dioxygenase Dapdiamide Biosynthesis Enzyme C. Why is the Substrate Tethered?

Jingyu Cao,[a,b] and Sam P. de Visser*[a,b]

[a] Miss J. Cao, Dr S. P. de Visser
Manchester Institute of Biotechnology
The University of Manchester
131 Princess Street, Manchester M1 7DN, United Kingdom
E-mail: sam.devisser@manchester.ac.uk

[b] Miss J. Cao, Dr S. P. de Visser
Department of Chemical Engineering
The University of Manchester
Oxford Road, Manchester M13 9PL, United Kingdom

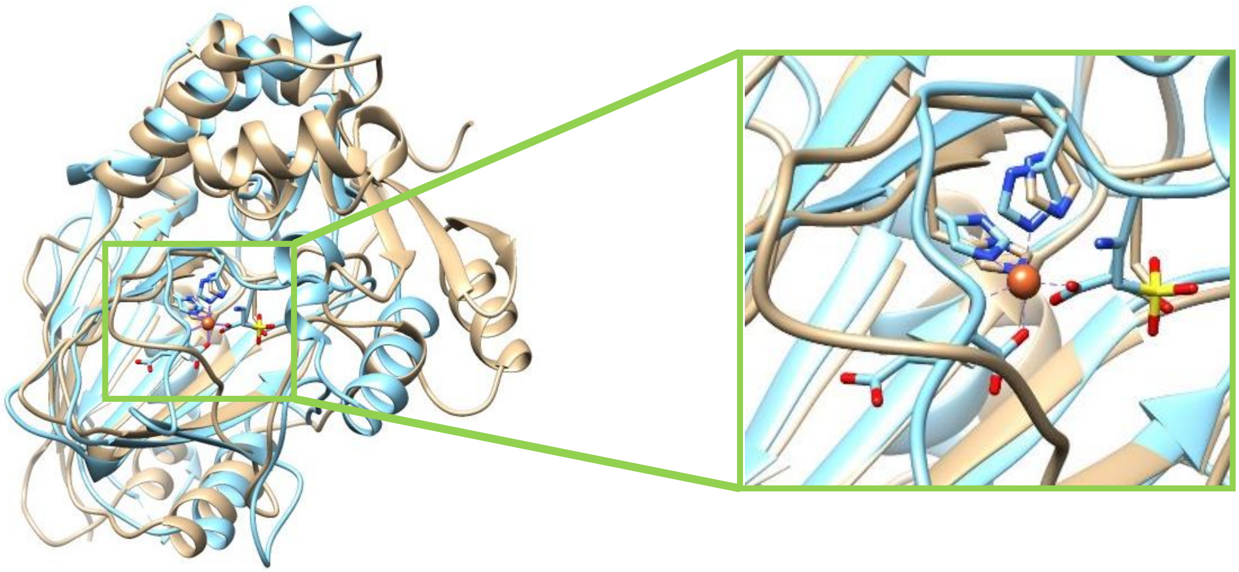

Figure S1: Overlay of the crystal structure coordinates of chain A of the 1GQW pdb file the computationally generated model of DdaC with iron(II) and αKG inserted (cyan: 1GQW; yellow: DdaC).

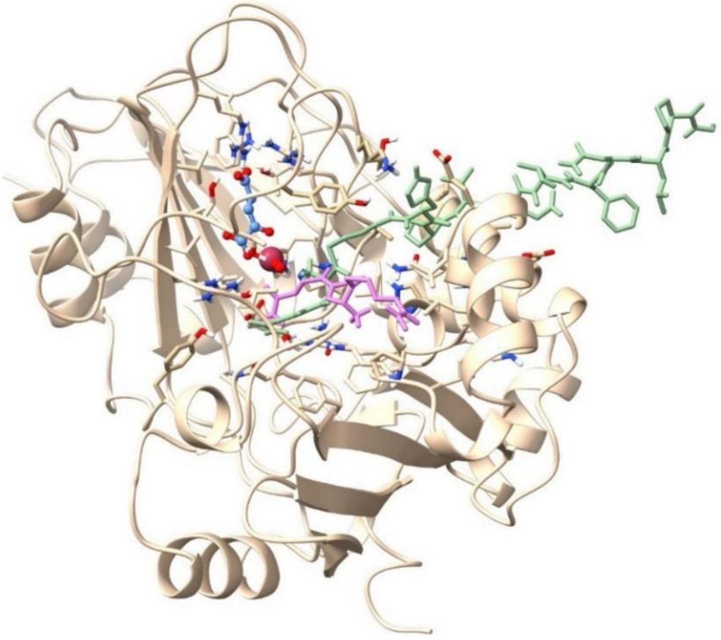

Figure S2: Overlay of the top-scoring docked structures for DdaC for the tethered (green) and non-tethered (magenta) substrate. The positions of the metal and its first-coordination sphere and oxygen were kept constant and αKG is shown in blue with heteroatoms.

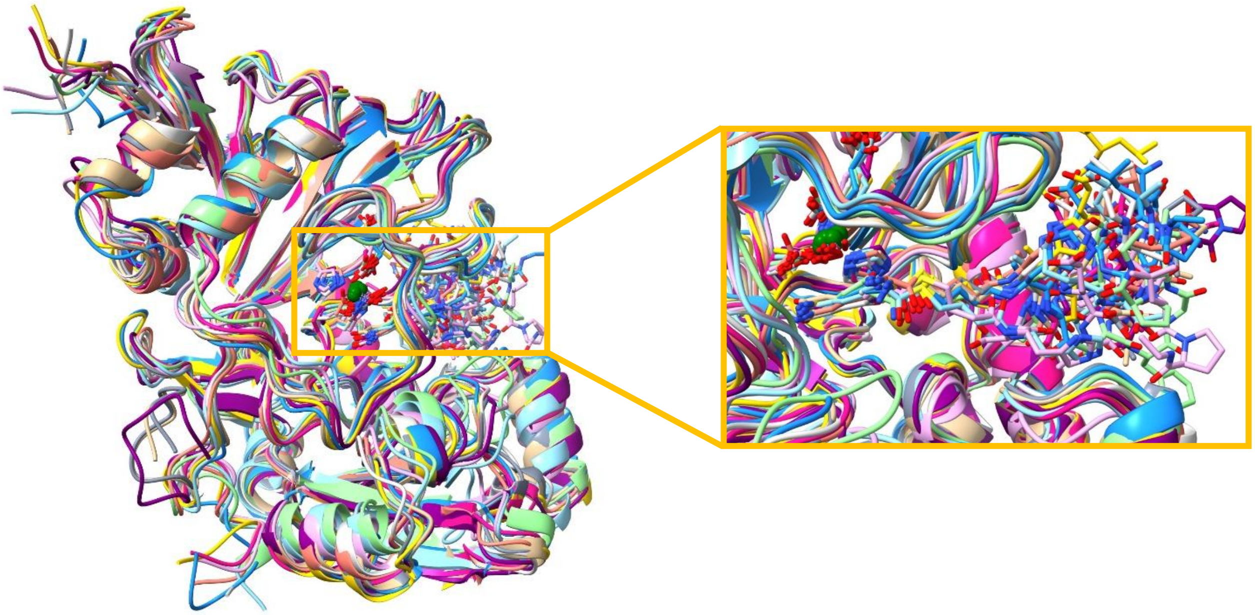

Figure S3: Overlay of the MD frames for the DdaC model with tethered substrate obtained after 0 ns (starting point, tan), 50 ns, 100 ns, 150 ns, 200 ns, 250 ns, 300 ns, 350 ns, 400 ns, 450 ns and 500 ns (other colours).

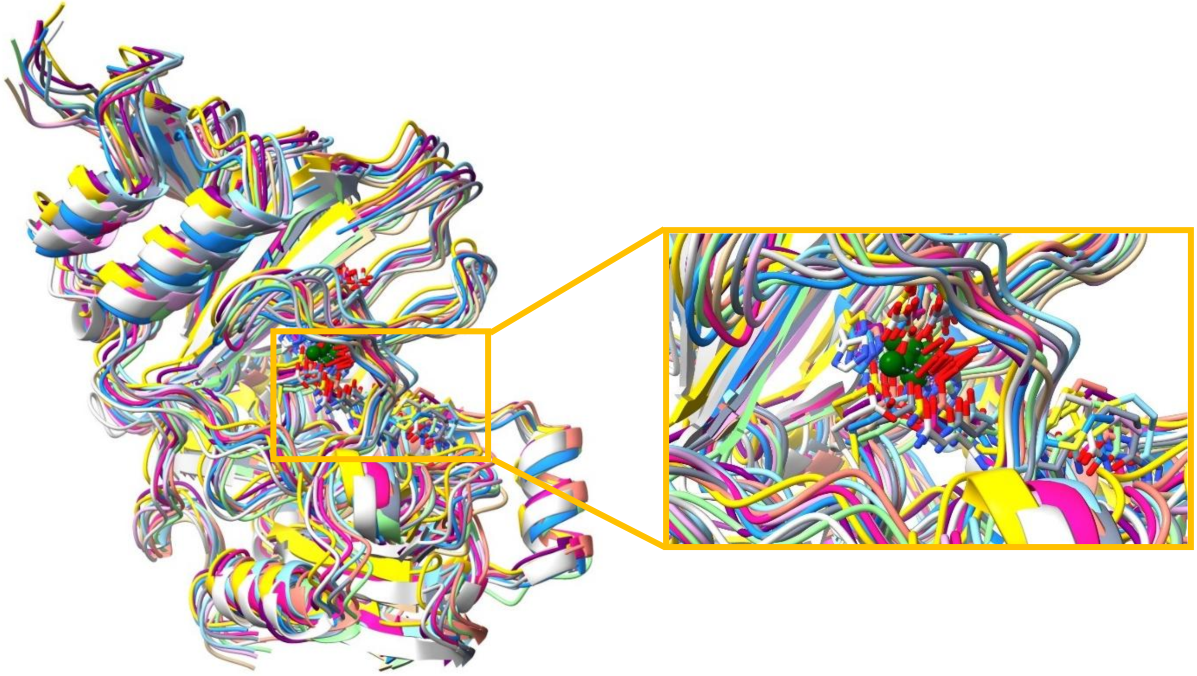

Figure S4: Overlay of the MD frames for the DdaC model with non-tethered substrate obtained after 0 ns (starting point, tan), 50 ns, 100 ns, 150 ns, 200 ns, 250 ns, 300 ns, 350 ns, 400 ns, 450 ns and 500 ns (other colours).

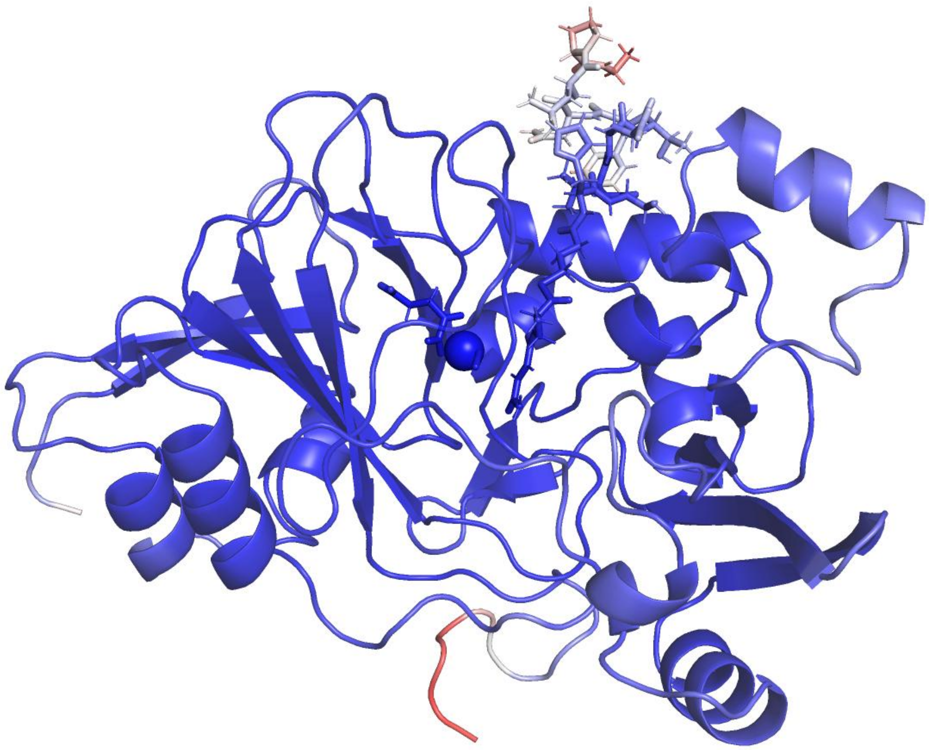

Figure S5: The B-factors (Å²) of all atoms for the dynamics of the DdaC model with tethered substrate during the 500 ns MD simulation (red: high RMSF, blue: low RMSF).

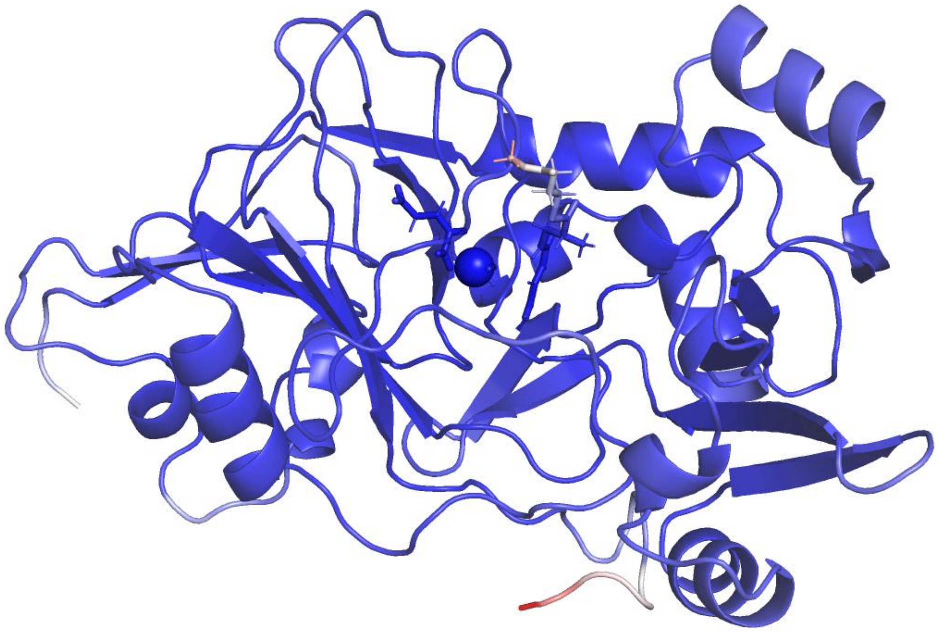

Figure S6: The B-factors (Å²) of all atoms for the dynamics of the DdaC model with non-tethered substrate during the 500 ns MD simulation (red: high RMSF, blue: low RMSF).

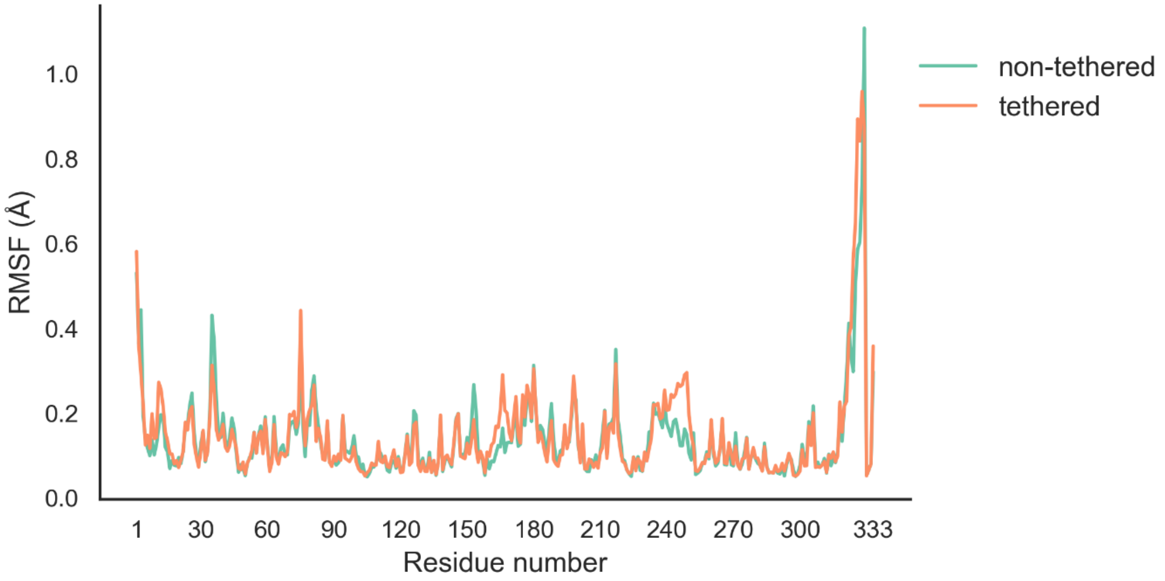

Figure S7: RMSF (Å²) of all residues for the dynamics of the DdaC model with non-tethered and tethered substrate during the 500 ns MD simulation.

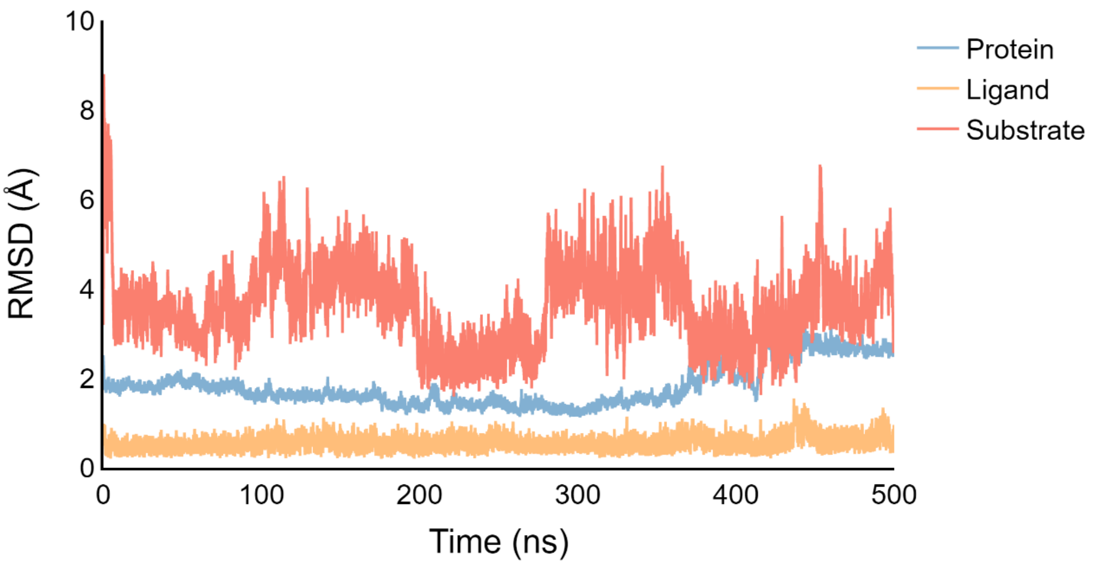

Figure S8: RMSD plot of the 500 ns MD simulation for the DdaC model with tethered substrate.

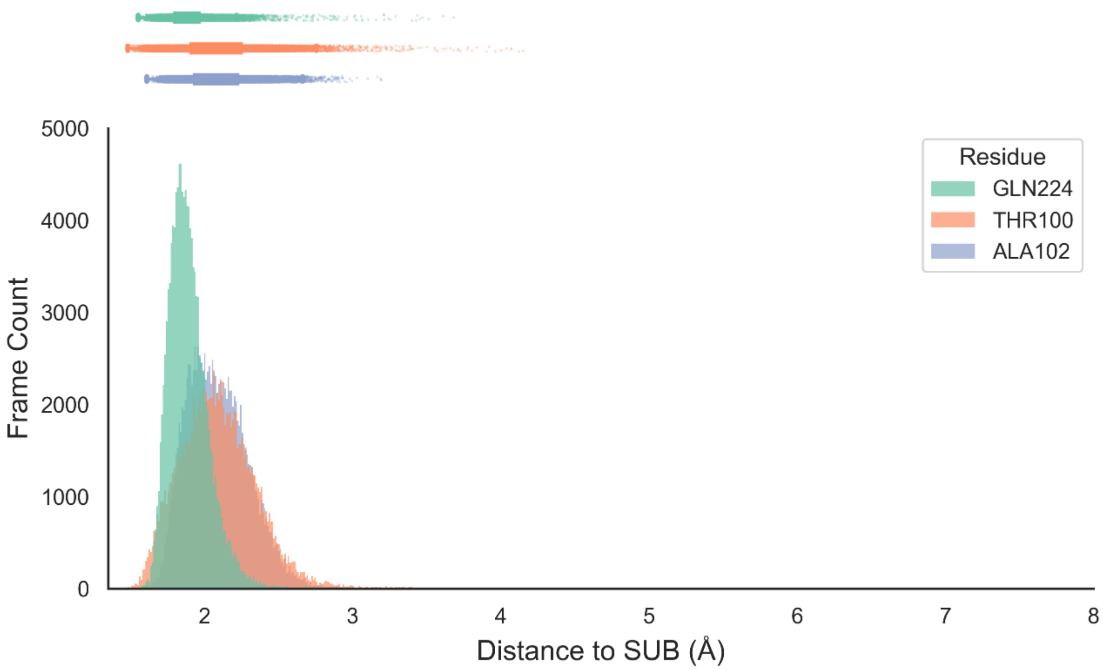

Figure S9: Radial Distribution Function plot of the top three hydrogen-bond interactions between residues of the backbone and the substrate during the 500 ns MD simulation for the DdaC model with tethered substrate.

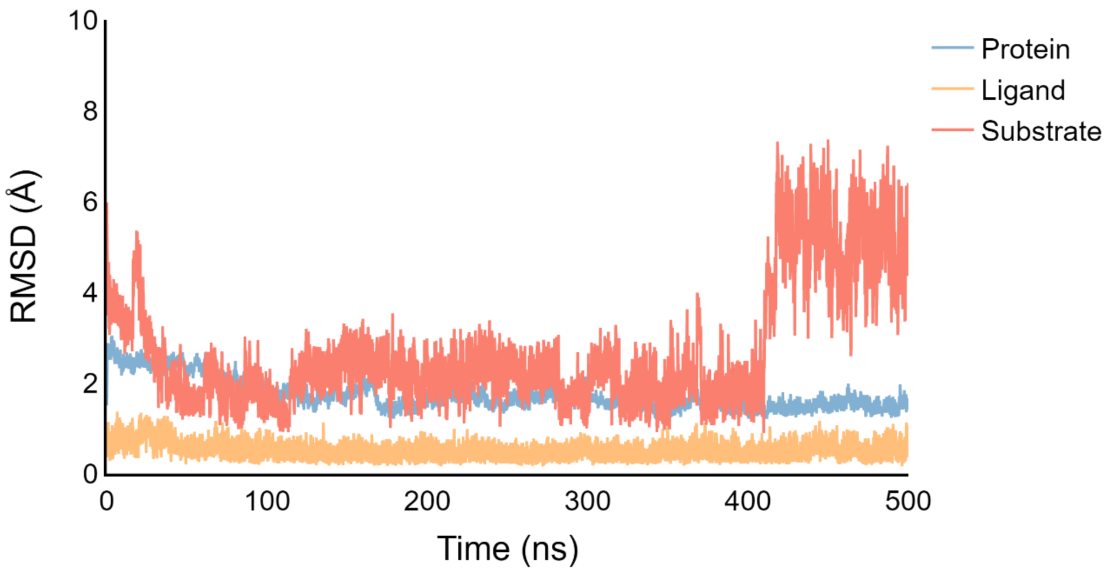

Figure S10: RMSD plot of the 500 ns MD simulation for the DdaC model with non-tethered substrate.

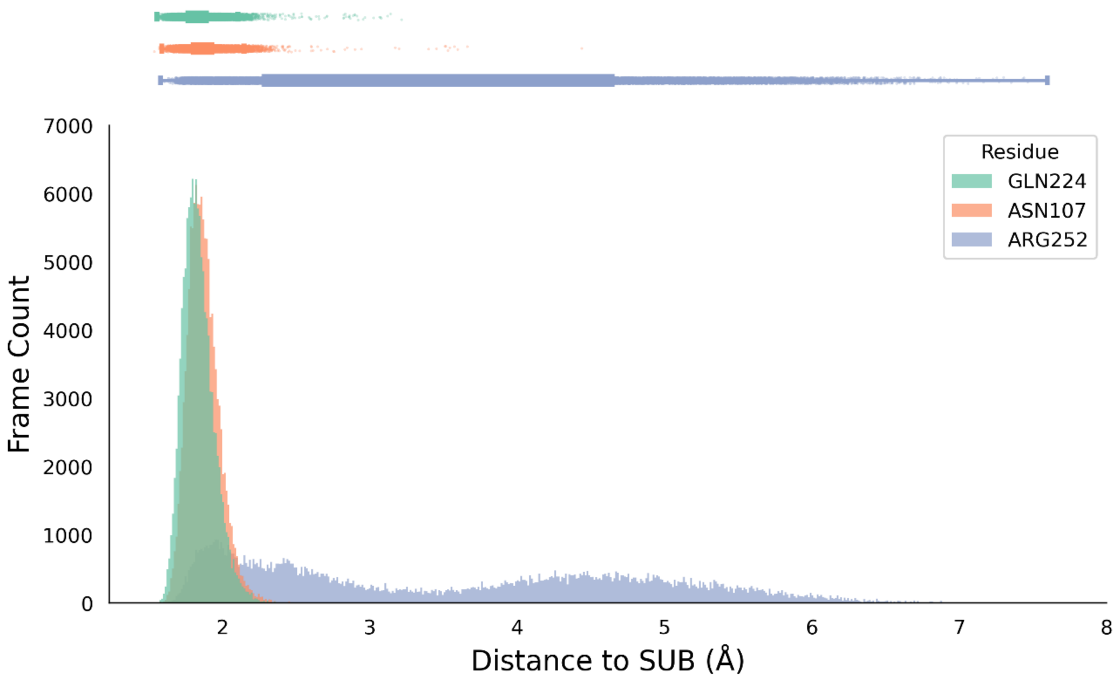

Figure S11: Radial Distribution Function plot of the top three hydrogen-bond interactions between residues of the backbone and the substrate during the 500 ns MD simulation for the DdaC model with non-tethered substrate.

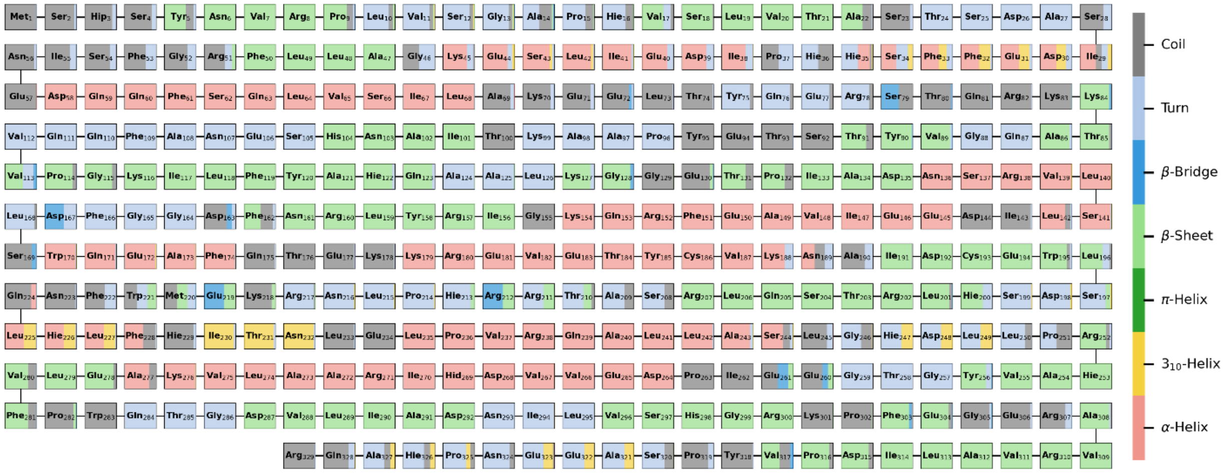

Figure S12: Secondary structure analysis by STRIDE approach of the protein during the 500 ns MD simulation for the DdaC model with tethered substrate. The surface area of each colored segment is proportional to the occupancy frequency of the corresponding secondary structure type at each residue.

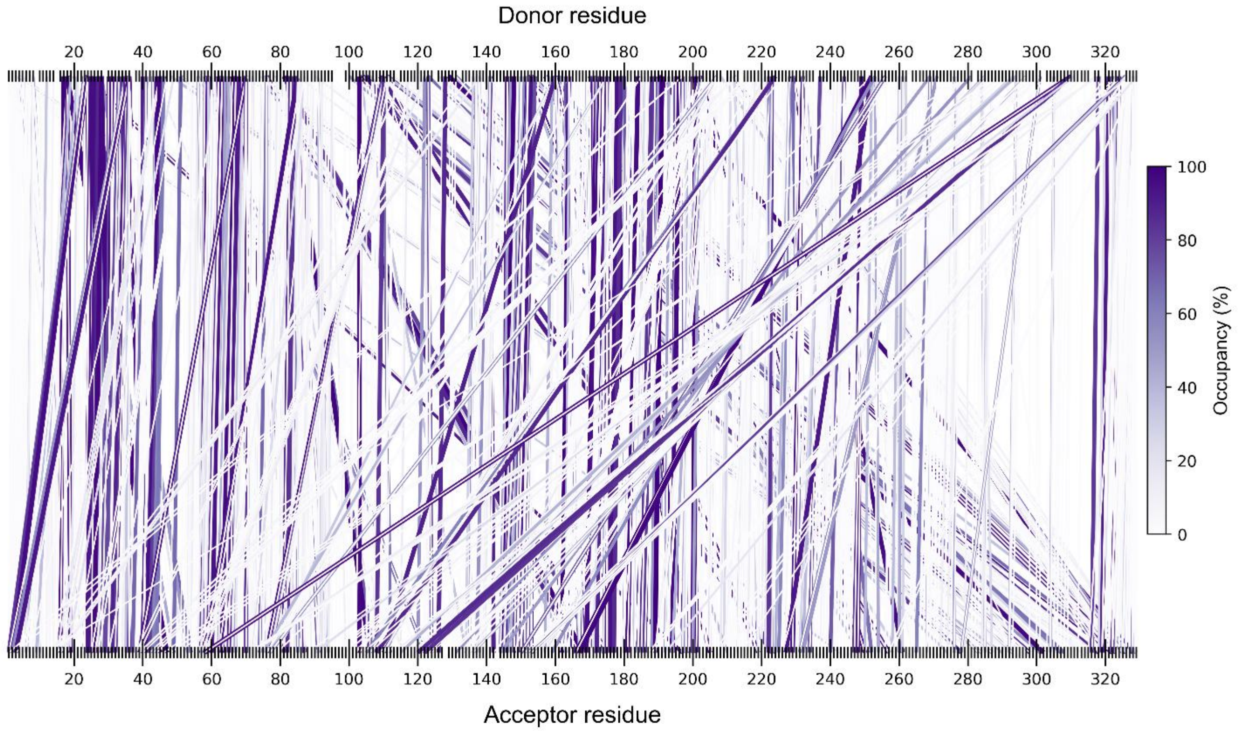

Figure S13: Hydrogen bond analysis performed using GROMACS over the 500 ns MD simulation of the DdaC model with tethered substrate, evaluating inter-residue hydrogen bonding patterns. Line intensity correlates positively with occupancy.

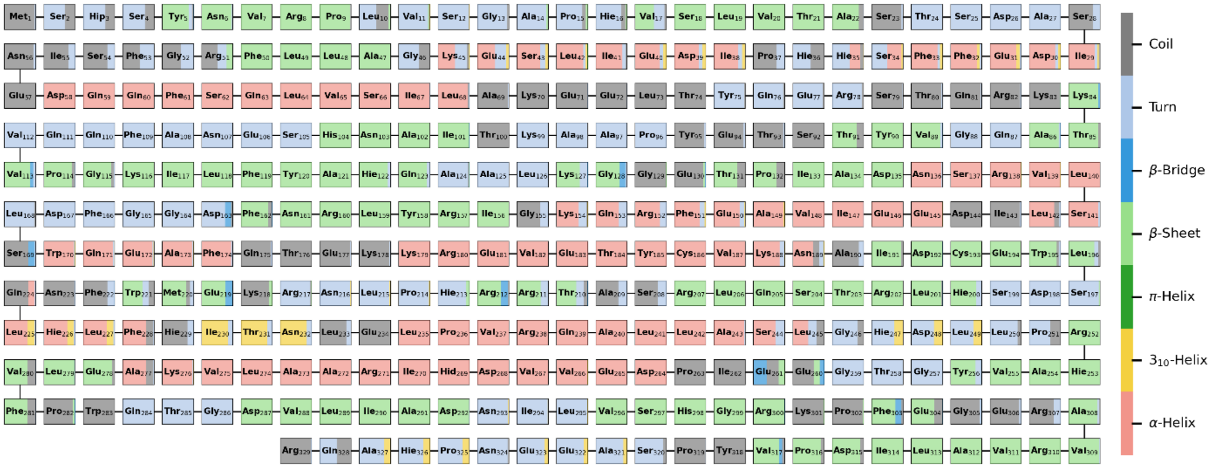

Figure S14: Secondary structure analysis by STRIDE approach of the protein during the 500 ns MD simulation for the DdaC model with non-tethered substrate. The surface area of each colored segment is proportional to the occupancy frequency of the corresponding secondary structure type at each residue.

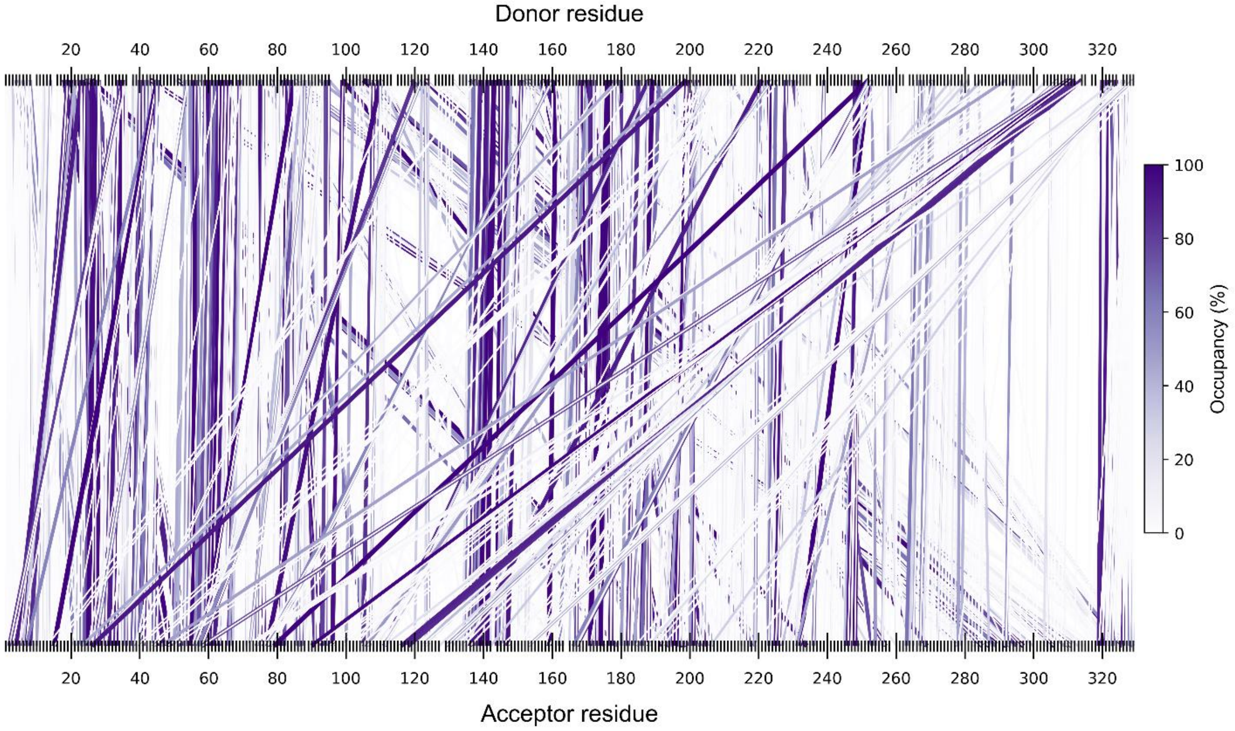

Figure S15: Hydrogen bond analysis performed using GROMACS over the 500 ns MD simulation of the DdaC model with non-tethered substrate, evaluating inter-residue hydrogen bonding patterns. Line intensity correlates positively with occupancy.

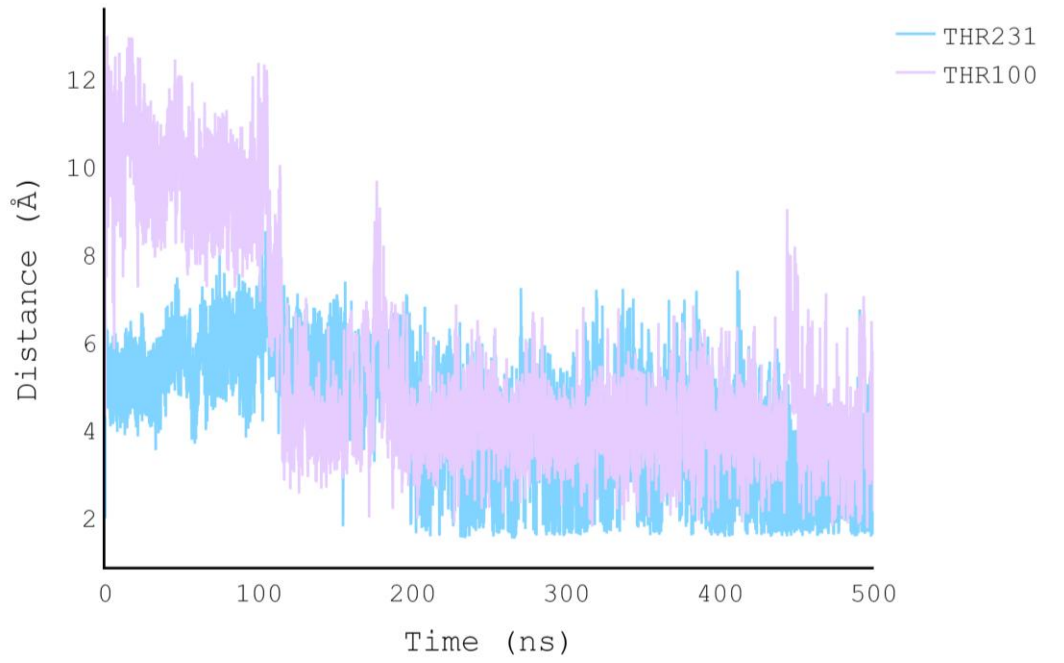

Figure S16: Distance plot of the hydrogen-bond interactions found between residues of the protein backbone and the substrate during the 500 ns MD simulation for the DdaC model with tethered substrate.

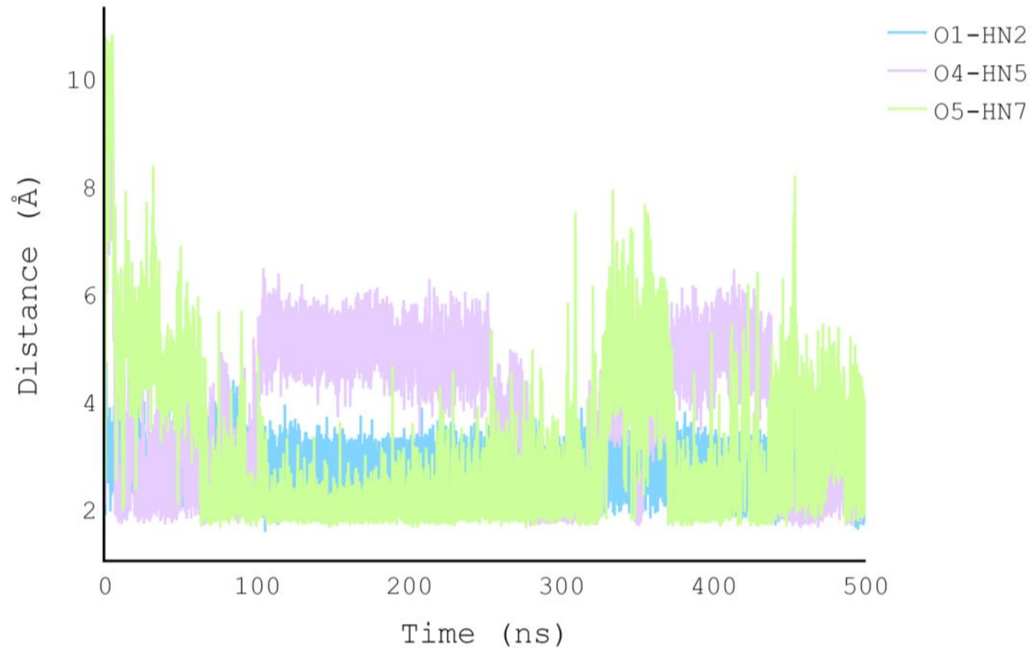

Figure S17: Distance plot of the intramolecular hydrogen-bonding interactions found at the tail covalently attached to DdaD during the 500 ns MD simulation for the DdaC model with tethered substrate.

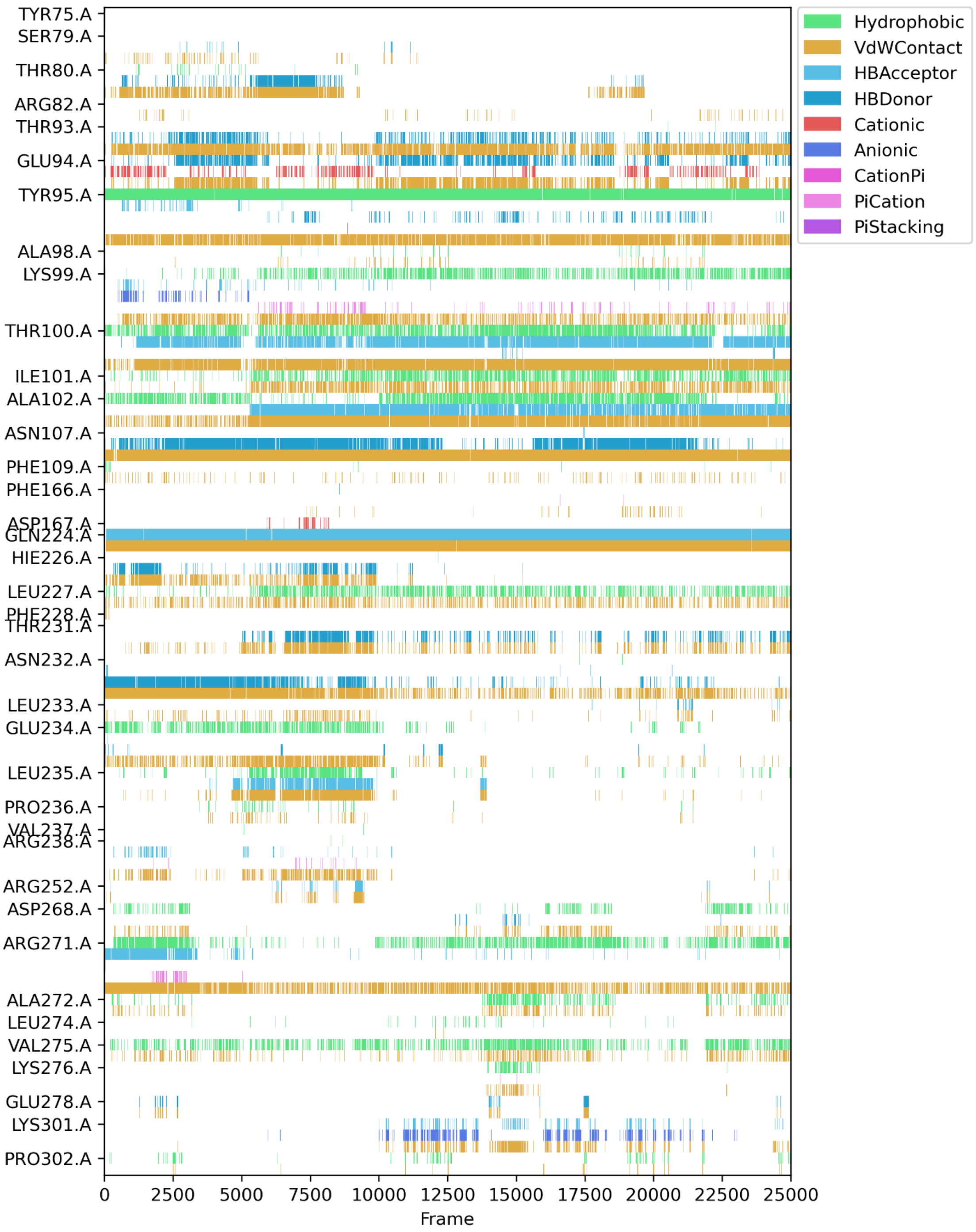

Figure S18: Residue-substrate interaction fingerprint during the 500 ns MD simulation for the DdaC model with tethered substrate. Hydrogen bonds defined in the fingerprint analysis between residues and the substrate followed the cutoff of 3.5 Å and 130°-180° (Donor-H-Acceptor), while in the secondary structure analysis among residue pairs the cutoff was 3.5 Å and 30°(H-Donor-Acceptor). All Hydrogen-bond analysis was based on the instantaneous structures over the whole 500 ns MD trajectory.

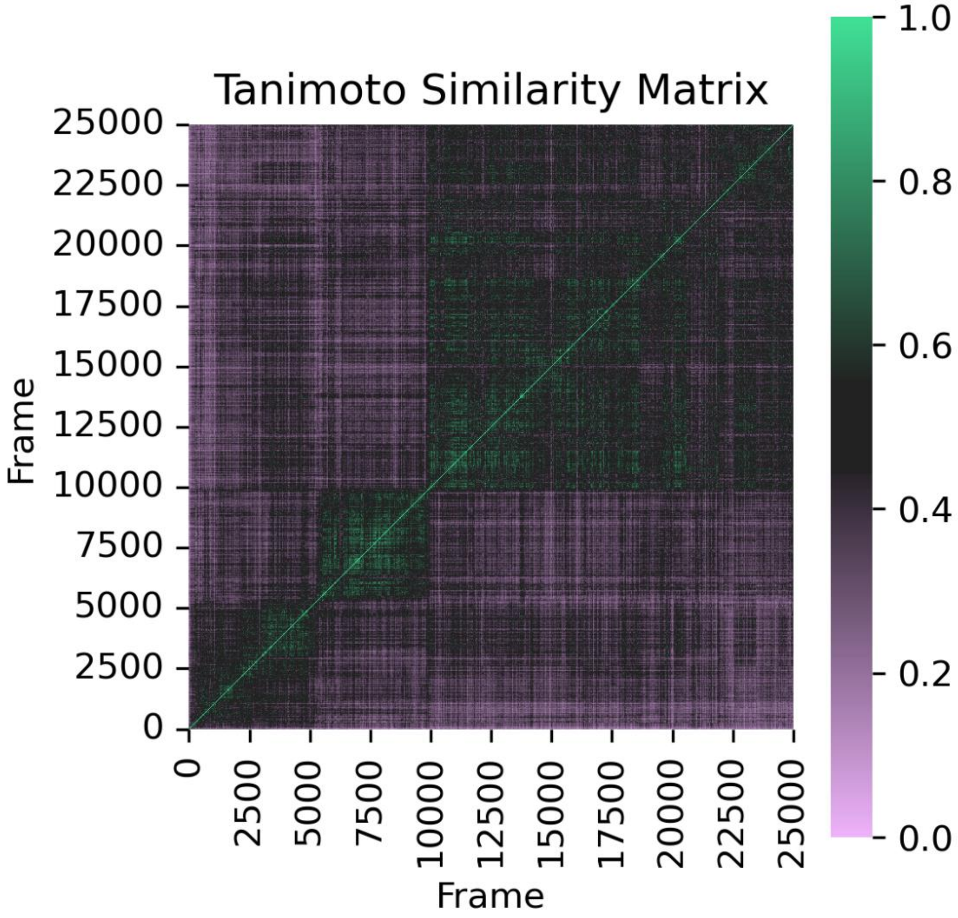

Figure S19: Tanimoto similarity matrix during the 500 ns MD simulation for the DdaC model with tethered substrate.

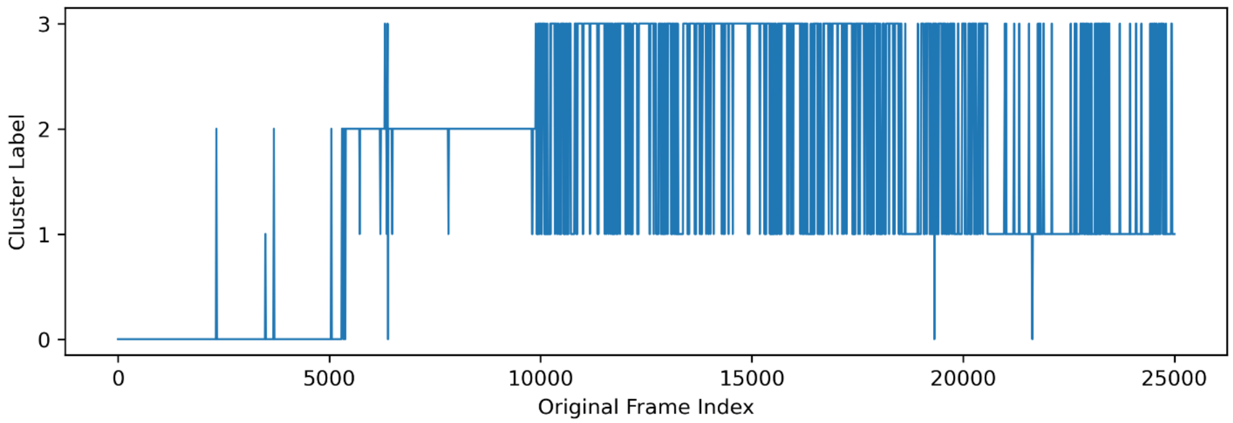

Figure S20: Agglomerative clustering analysis during the 500 ns MD simulation for the DdaC model with tethered substrate.

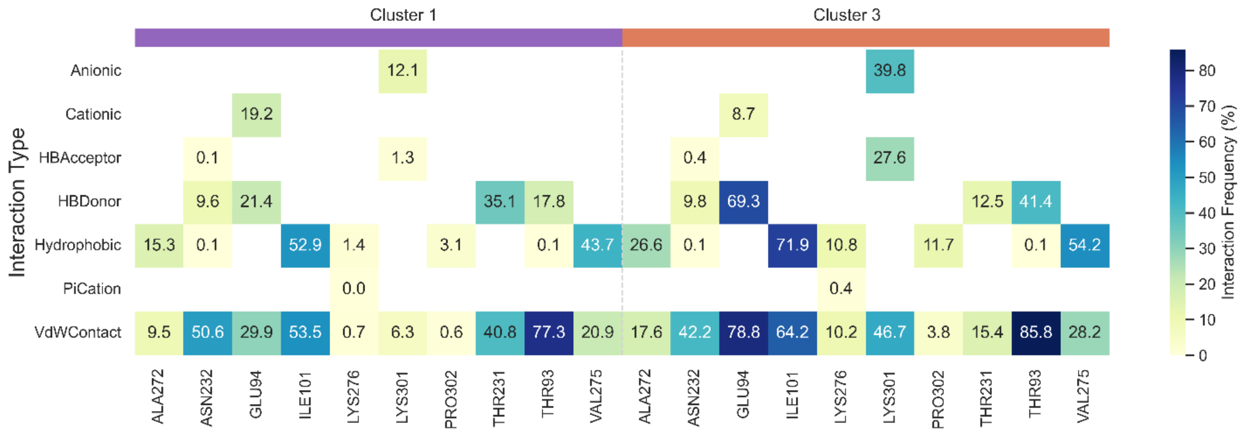

Figure S21: Top 10 residue-substrate interaction differences between Cluster 1 and Cluster 3 during the 500 ns MD simulation for the DdaC model with tethered substrate.

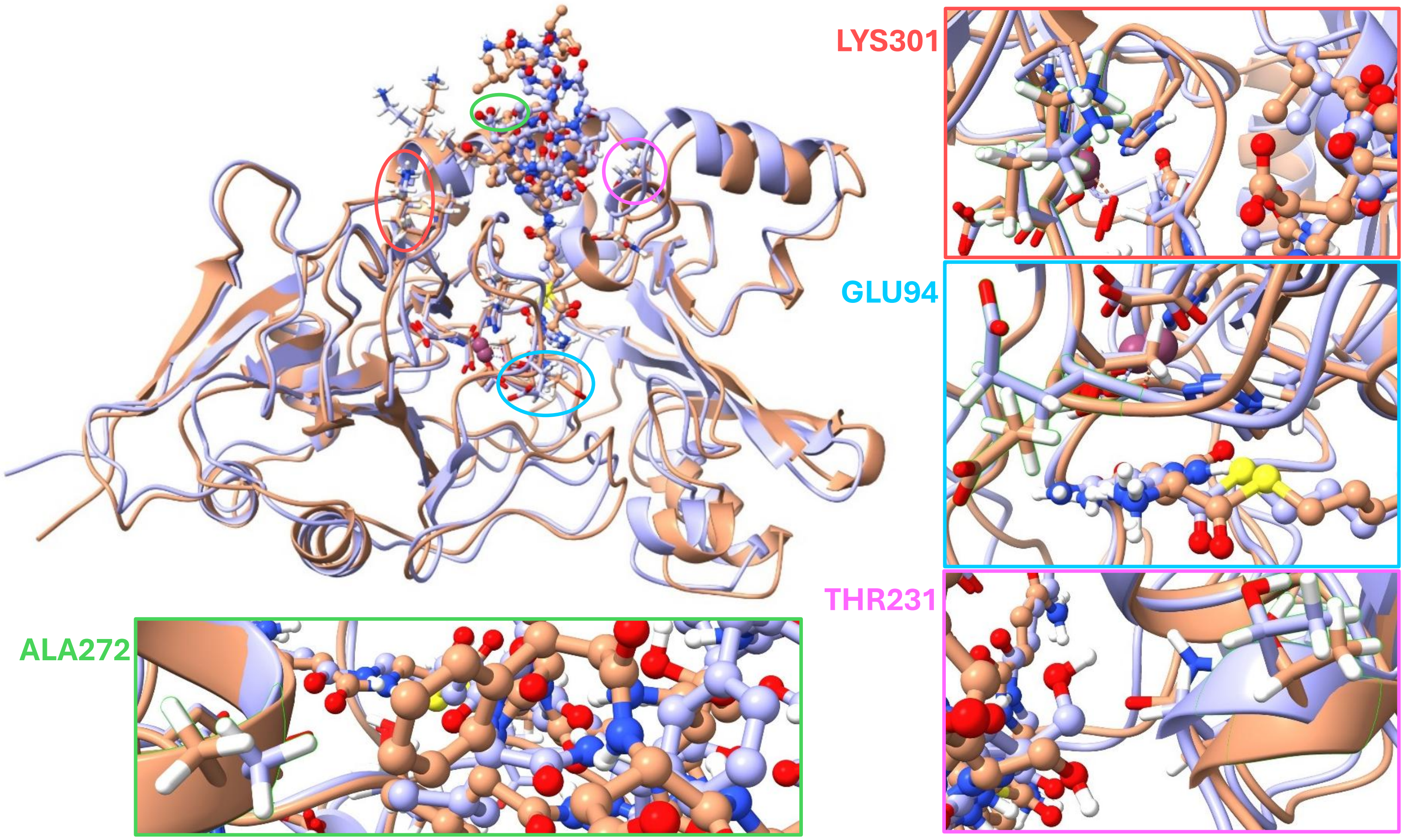

Figure S22: Overlay of the average frames of Cluster 1 (purple) and Cluster 3 (orange) for the DdaC model with tethered substrate. The top 10 residues with the largest interaction discrepancy are shown in stick, and the substrates are shown in ball stick.

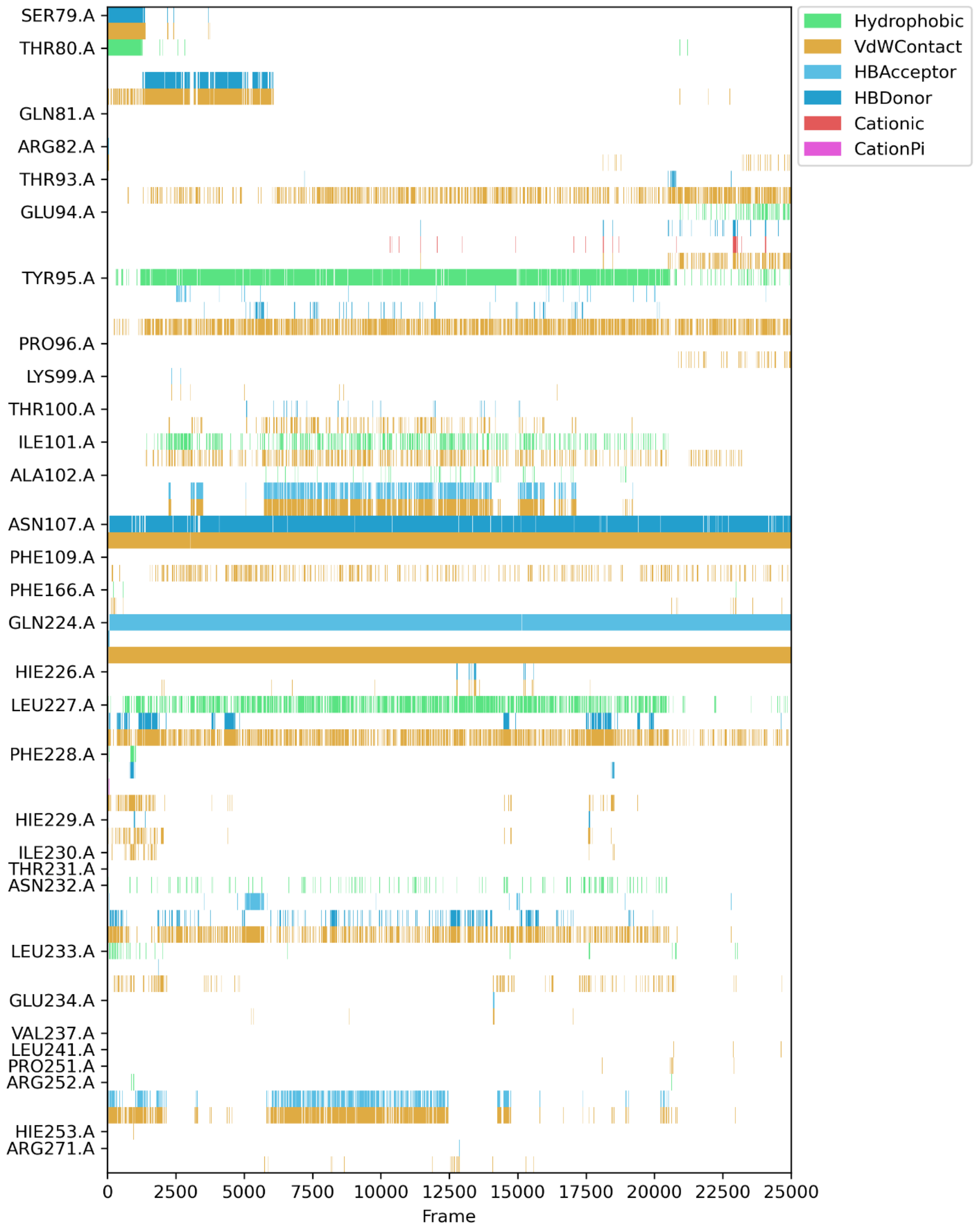

Figure S23: Residue-substrate interaction fingerprint during the 50 ns MD simulation for the DdaC model with non-tethered substrate. Hydrogen bonds defined in the fingerprint analysis between residues and the substrate followed the cutoff of 3.5 Å and 130°-180° (Donor-H-Acceptor), while in the secondary structure analysis among residue pairs the cutoff was 3.5 Å and 30°(H-Donor-Acceptor). All Hydrogen-bond analysis was based on the instantaneous structures over the whole 500 ns MD trajectory.

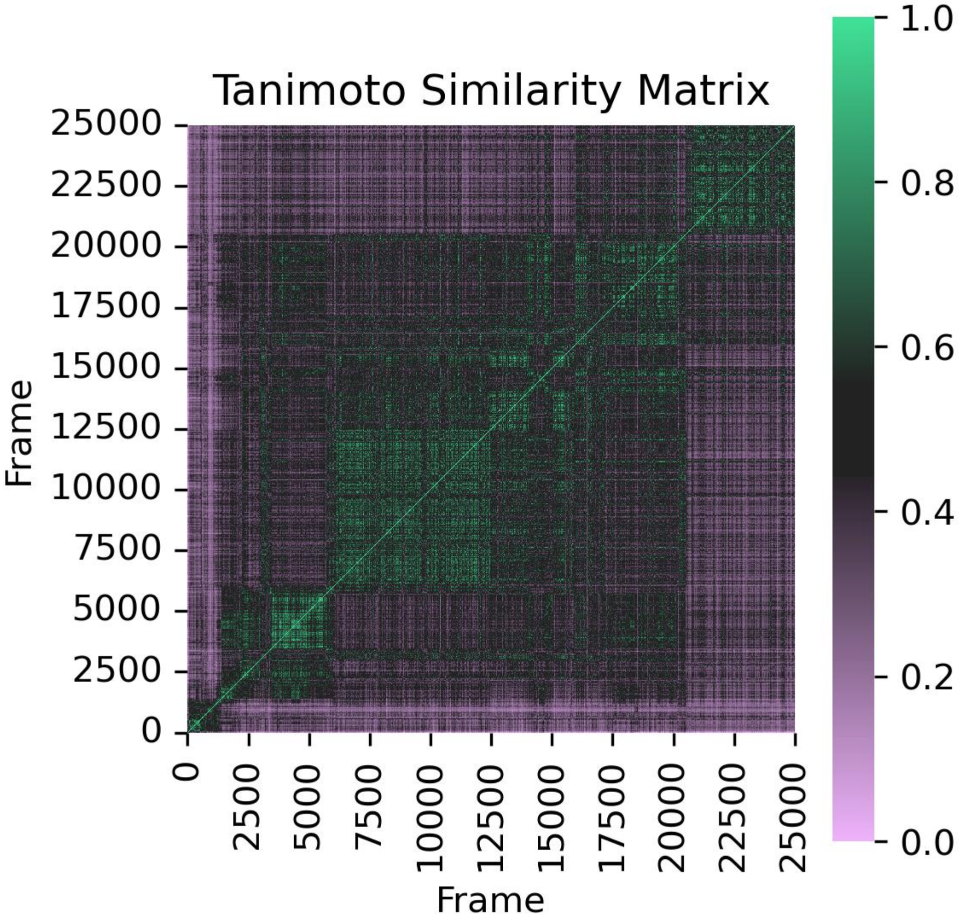

Figure S24: Tanimoto similarity matrix during the 500 ns MD simulation for the DdaC model with non-tethered substrate.

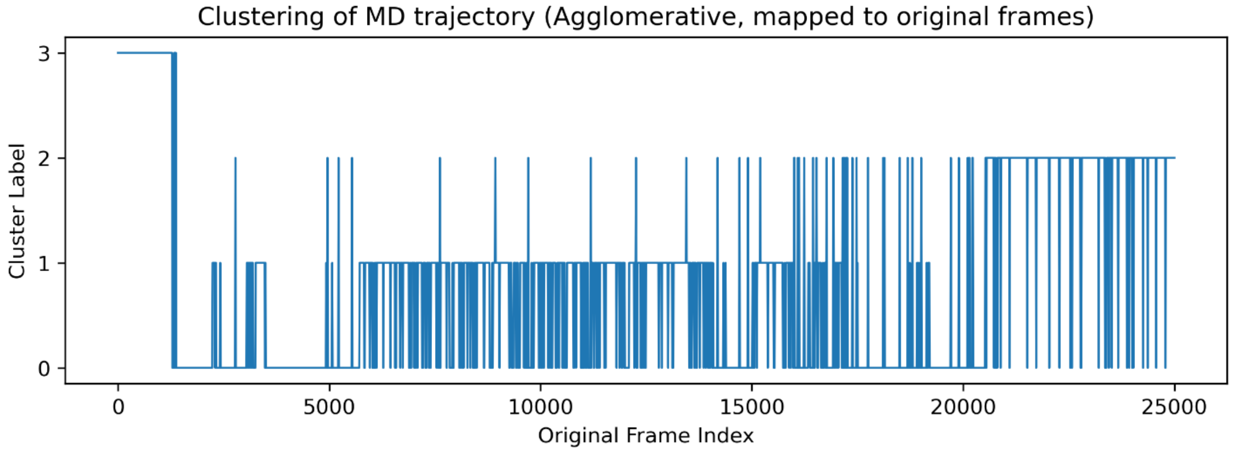

Figure S25: Agglomerative clustering analysis during the 500 ns MD simulation for the DdaC model with non-tethered substrate.

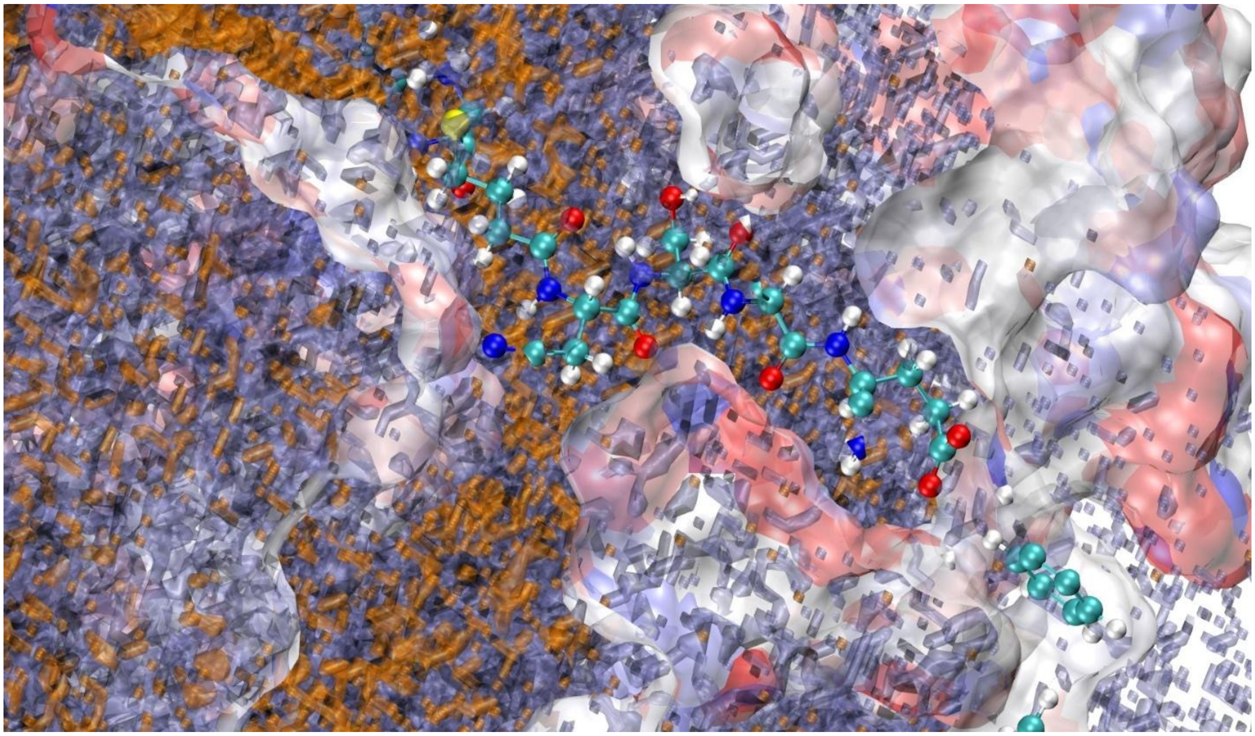

Figure S26: Thermodynamic mapping of water within the binding pocket of the DdaC model with tethered substrate performed using Grid Inhomogeneous Solvation Theory (GIST) over a 500 ns MD simulation. The solvent accessible surface (SASA; rendered in iceblue) represents voxels on the protein surface where water oxygens were observed. Overlaid in orange are voxels exhibiting both elevated water occupancy (g_O > 0.9) and unfavorable solvent–protein interaction energy (E_{tot} > -0.25 kcal·mol^{-1}), thereby identifying hydration sites whose displacement may contribute most favorably to ligand binding.

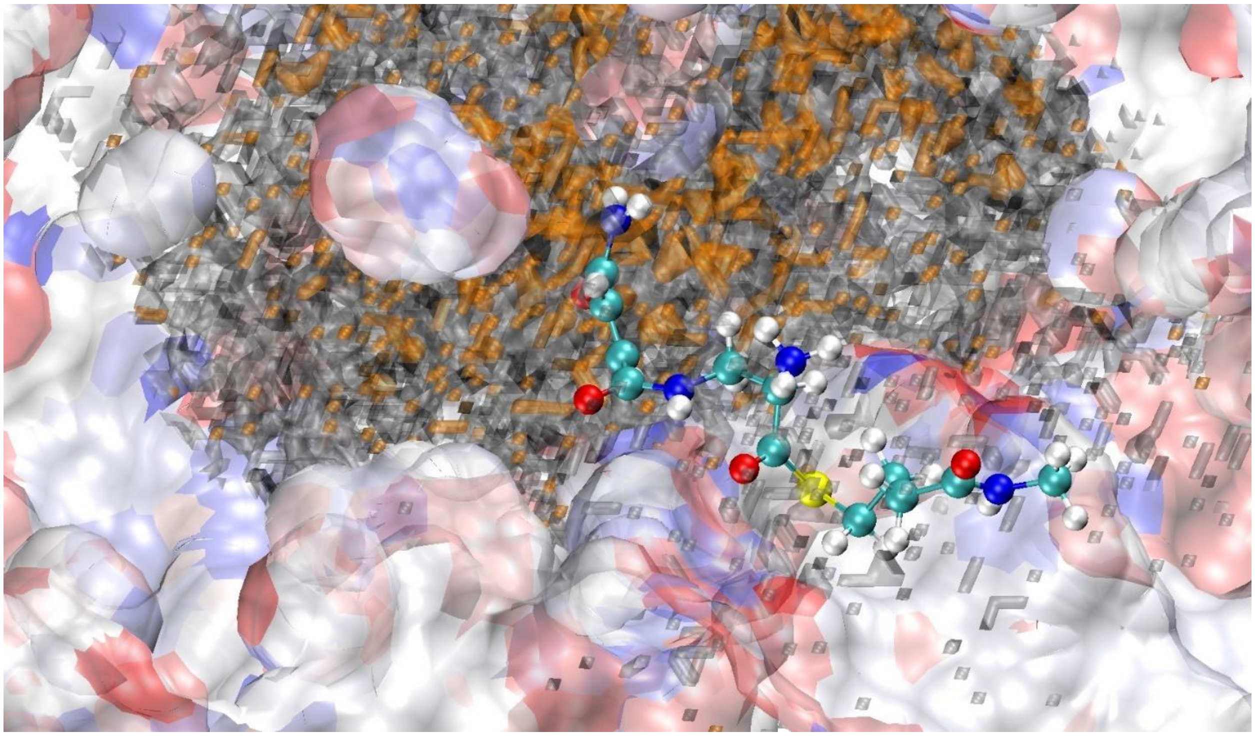

Figure S27: Thermodynamic mapping of water within the binding pocket of the DdaC model with non-tethered substrate performed using Grid Inhomogeneous Solvation Theory (GIST) over a 500 ns MD simulation. The solvent accessible surface (SASA; rendered in silver) represents voxels on the protein surface where water oxygens were observed. Overlaid in orange are voxels exhibiting both elevated water occupancy (g_O > 0.9) and unfavorable solvent-protein interaction energy (E_{tot} > -0.25 kcal·mol^{-1}), thereby identifying hydration sites whose displacement may contribute most favorably to ligand binding.

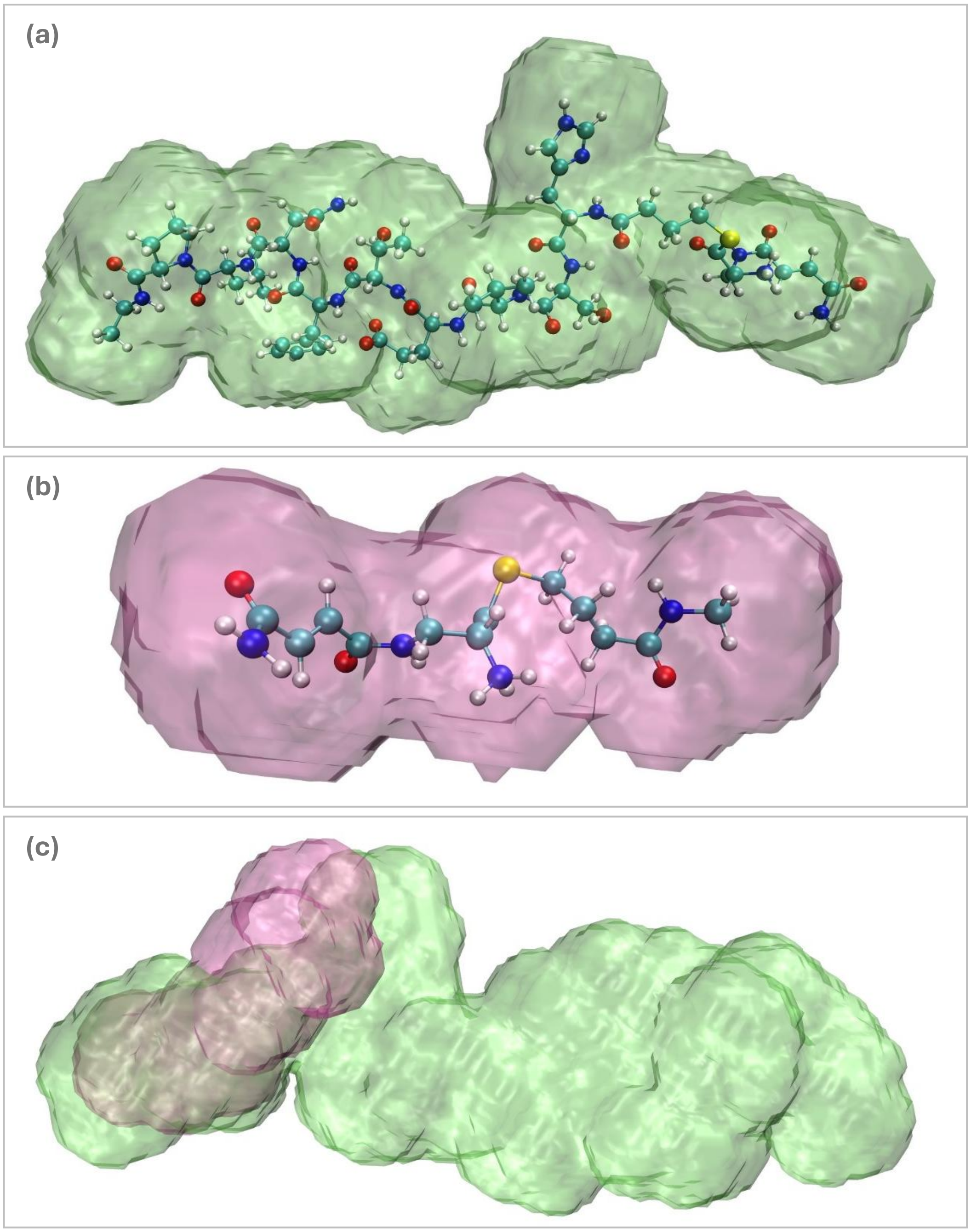

Figure S28: The voxels above define the region within 3 Å of any heavy atom of the ligand binding with the protein DdaC over a 500 ns MD simulation, estimating the volume of water that will be expelled from the binding surface. (a: tethered substrate; b: non-tethered substrate; c: overlap of both models)

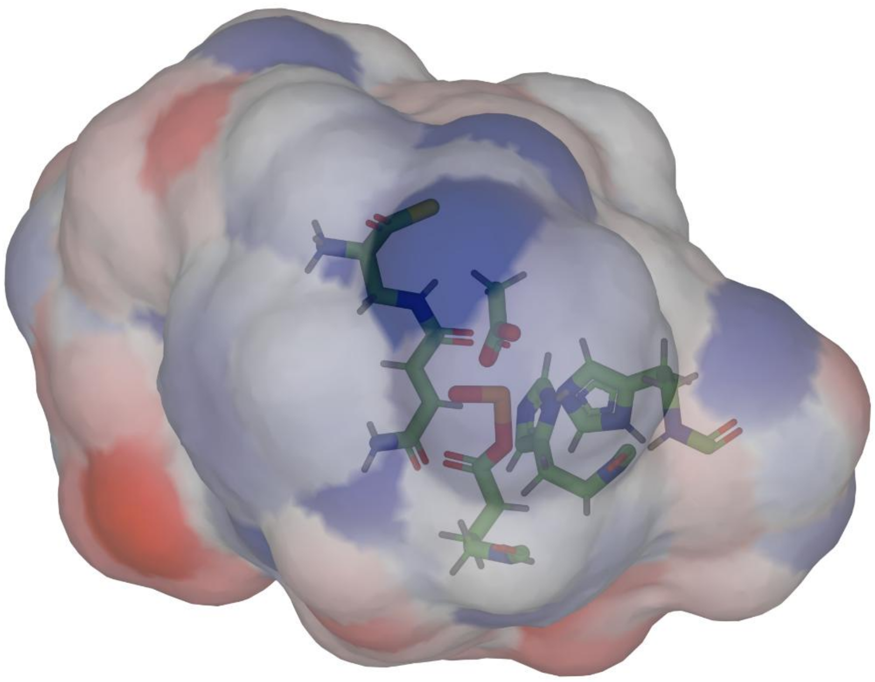

Figure S29: Binding cavity in the cluster model of DdaC with tethered substrate at TS1. The first-coordination sphere and N_βFmmDAP are shown in stick. The ESP values (in kcal mol^{-1}) ranged from -34.02 to 94.72 are colored from blue (negative) to red (positive).

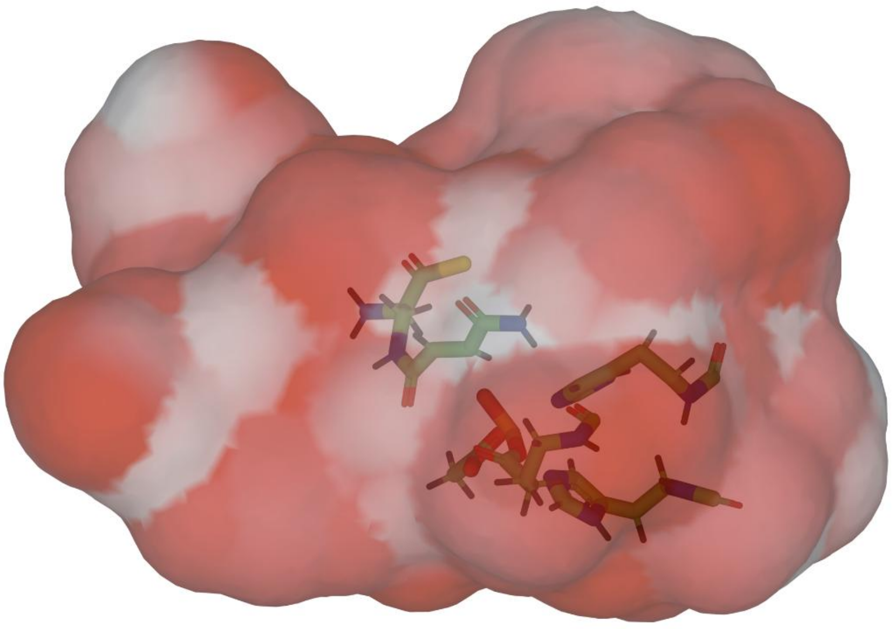

Figure S30: Binding cavity in the cluster model of DdaC with non-tethered substrate at TS1. The first-coordination sphere and N_βFmmDAP are shown in stick. The ESP values (in kcal mol^{-1}) ranged from 13.16 to 118.11 are colored from blue (negative) to red (positive).

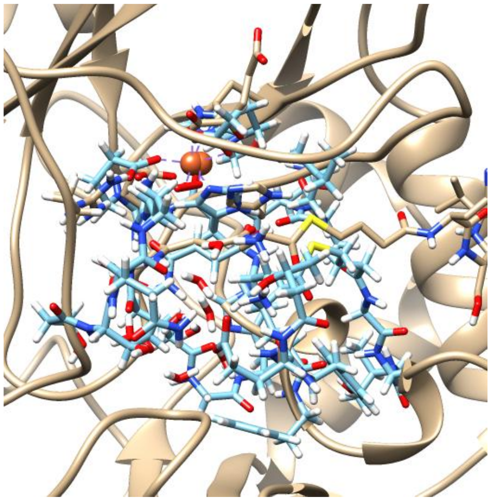

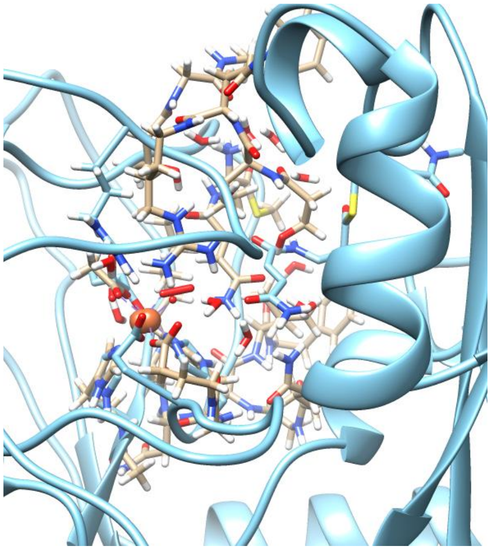

Overlay $^{5}Re_{A}$ vs average MD_{I}

Overlay $^{5}Re_{B}$ vs average MD_{II}

Figure S31: Overlays of the optimized geometries of the reactant complexes with the average snapshot of the MD simulation the cluster models were taken from. As can be seen the overlays give a good match and show little changes between the cluster model and the MD snapshots.

Table S1: Frequency of top 10 residue-substrate interaction types for the DdaC model with tethered substrate during the 500 ns MD simulation.

**residue**	GLN224	ASN107	TYR95	GLN224	THR100
**interaction**	VdWContact	VdWContact	Hydrophobic	HBAcceptor	VdWContact
**frequency (%)**	99.7	99.6	99.4	99.4	93.2
**residue**	TYR95	ALA102	THR100	THR93	ALA102
**interaction**	VdWContact	VdWContact	HBAcceptor	VdWContact	HBAcceptor
**Frequency (%)**	88.1	86.6	83.4	80.1	71.1

Table S2: Frequency of top 10 residue-substrate interaction types for the DdaC model with non-tethered substrate during the 500 ns MD simulation.

**residue**	ASN107	GLN224	GLN224	ASN107	TYR95
**interaction**	VdWContact	VdWContact	HBAcceptor	HBDonor	Hydrophobic
**frequency (%)**	99.9	99.8	99.6	96.4	77.1
**residue**	LEU227	LEU227	TYR93	ASN232	PHE109
**interaction**	VdWContact	HBDonor	VdWContact	VdWContact	VdWContact
**Frequency (%)**	65.6	50.8	44.4	42.3	34.9

Table S3: Frequency of top 10 residue-substrate interaction differences between Cluster 1 and Cluster 3 during the 500 ns MD simulation for the DdaC model with tethered substrate.

residue	Cluster1 (%)	Cluster3 (%)	Difference (%)
LYS301	14.6	55.1	40.5
GLU94	44.7	81.4	36.7
THR231	41.1	16.1	25.0
ILE101	67.1	80.6	13.4
LYS276	1.9	13.8	11.9
ALA272	18.0	29.1	11.1
PRO302	3.2	12.4	9.2
VAL275	47.4	56.5	9.1
ASN232	50.8	42.2	8.7
THR93	77.6	86.2	8.6

Table S4: Energy component (in kcal mol^{-1}) obtained by MM-PBSA analysis for all featured frames of Cluster 1 and Cluster 3 and the whole trajectory during the 500 ns MD simulation for the DdaC model with tethered substrate. (SEM: Standard Error of the Mean)

	Cluster1	SEM	Cluster3	SEM	whole	SEM
**Van der Waals**	-69.21	0.19	-72.01	0.18	-74.80	0.53
**Electrostatic**	-38.23	0.52	-47.56	0.49	-50.76	1.08
**Polar Solvation**	56.00	0.49	63.28	0.46	69.05	1.13
**Non-Polar (SASA+SAV)**	-47.71	0.13	-50.65	0.13	-52.21	0.35
**Dispersion**	90.94	0.18	94.23	0.18	97.61	0.53
**ΔG_gas**	-107.44	0.57	-119.57	050	-125.56	1.40
**ΔG_solv**	99.24	0.52	106.86	0.48	114.45	1.27
**ΔG_total**	-8.20	0.20	-12.70	0.19	-11.11	0.38

Table S5: Per-residue free energy (in kcal mol^{-1}) decomposition by MM-PBSA for selected residues for all featured frames of Cluster 1 during the 500 ns MD simulation for the DdaC model with tethered substrate. (SEM: Standard Error of the Mean)

residue	Van der Waals	SEM	Electrostatic	SEM	Polar Solvation	SEM	Total	SEM
LYS301	-0.29	0.008	-13.71	0.225	6.78	0.111	-7.22	0.122
GLU94	-0.26	0.011	-19.47	0.236	9.20	0.113	-10.53	0.127
THR231	-0.36	0.012	-0.88	0.046	0.46	0.013	-0.79	0.034
ILE101	-2.34	0.011	0.37	0.017	0.11	0.005	-1.86	0.019
LYS276	-0.13	0.016	-5.66	0.007	2.78	0.003	-3.01	0.020
ALA272	-0.51	0.007	0.12	0.062	-0.08	0.032	-0.47	0.033
PRO302	-0.33	0.008	-0.19	0.011	0.11	0.005	-0.41	0.012
VAL275	-1.03	0.023	-0.39	0.008	0.21	0.004	-1.21	0.023
ASN232	-1.38	0.018	-1.30	0.053	1.82	0.028	-0.86	0.035
THR93	-1.06	0.013	-0.19	0.064	0.43	0.021	-0.82	0.048

Table S6: Per-residue free energy (in kcal mol^{-1}) decomposition by MM-PBSA for selected residues for all featured frames of Cluster 3 during the 500 ns MD simulation for the DdaC model with tethered substrate. (SEM: Standard Error of the Mean)

residue	Van der Waals	SEM	Electrostatic	SEM	Polar Solvation	SEM	Total	SEM
LYS301	-0.40	0.015	-19.62	0.285	9.66	0.128	-10.36	0.164
GLU94	-0.27	0.016	-21.21	0.219	9.65	0.099	-11.83	0.122
THR231	-0.37	0.009	-0.51	0.038	0.40	0.012	-0.47	0.027
ILE101	-2.42	0.012	0.47	0.016	0.08	0.005	-1.87	0.020
LYS276	-0.27	0.013	-5.82	0.053	2.93	0.031	-3.16	0.031
ALA272	-0.63	0.019	0.19	0.007	-0.09	0.004	-0.54	0.020
PRO302	-0.43	0.010	-0.18	0.013	0.11	0.006	-0.50	0.014
VAL275	-1.35	0.027	-0.36	0.008	0.20	0.004	-1.51	0.025
ASN232	-1.34	0.018	-1.38	0.047	1.77	0.027	-0.95	0.032
THR93	-1.09	0.015	-1.41	0.048	0.88	0.015	-1.62	0.035

Table S7: Absolute (in au) energies and free energies of UB3LYP/BS1 optimized geometries for the epoxidation mechanism of the models with tethered and non-tethered substrate as calculated in Gaussian-16. Gc is the free energy correction at 298 K.

	E [BS1, au]	ZPE [au]	G [au]	E [BS2, au]	Gc [au]
^{5}**Re**$_A$	-11504.982268	3.389507	-11501.895416	-11516.319365	3.086852
^{5}**TS1**$_A$	-11504.968043	3.387080	-11501.880189	-11516.301293	3.087854
^{5}**IM1**$_A$	-11505.018381	3.388798	-11501.925255	-11516.346408	3.093126
^{5}**TS2**$_A$	-11505.003610	3.388984	-11501.91076	-11516.328464	3.092846
^{5}**P**$_A$	-11505.066236	3.391531	-11501.97073	-11516.385208	3.095508
^{5}**Re**$_B$	-9963.185381	2.876046	-9960.574561	-9972.810644	2.610820
^{5}**TS1**$_B$	-9963.159738	2.873022	-9960.549254	-9972.778680	2.610484
^{5}**IM1**$_B$	-9963.202744	2.874686	-9960.589557	-9972.813723	2.613187
^{5}**TS2**$_B$	-9963.191468	2.874385	-9960.577686	-9972.802246	2.613782
^{5}**P**$_B$	-9963.220532	2.877027	-9960.608652	-9972.834425	2.611880

Table S8: Relative (in kcal mol^{-1}) energies and free energies of UB3LYP/BS1 optimized geometries for the epoxidation mechanism of the models with tethered and non-tethered substrate as calculated in Gaussian-16.

	ΔE [BS1]	ΔE+ZPE	ΔG [BS1]	ΔE [BS2]	ΔE+ZPE	ΔG [BS2]
^{5}**Re**$_A$	0.00	0.00	0.00	0.00	0.00	0.00
^{5}**TS1**$_A$	8.93	7.40	9.56	11.34	9.82	11.97
^{5}**IM1**$_A$	-22.66	-23.11	-18.72	-16.97	-17.41	-13.03
^{5}**TS2**$_A$	-13.39	-13.72	-9.63	-5.71	-6.04	-1.95
^{5}**P**$_A$	-52.69	-51.42	-47.26	-41.32	-40.05	-35.89
^{5}**Re**$_B$	0.00	0.00	0.00	0.00	0.00	0.00
^{5}**TS1**$_B$	16.09	14.19	15.88	20.06	18.16	19.85
^{5}**IM1**$_B$	-10.90	-11.75	-9.41	-1.93	-2.79	-0.45
^{5}**TS2**$_B$	-3.82	-4.86	-1.96	5.27	4.23	7.13
^{5}**P**$_B$	-22.06	-21.44	-21.39	-14.92	-14.31	-14.26

Table S9: Mulliken group charges (Q) and spin densities (ρ) of UB3LYP/BS1 optimized geometries for the epoxidation mechanism of the models with tethered and non-tethered substrate as calculated in Gaussian-16. Succ stands for succinate, Sub for *N*$_β$FmmDAP substrate and Prot all protein atoms.

Charge	Q(Fe)	Q(O)	Q(Succ)	Q(Sub)	Q(Prot)	Q(Total)
^{5}**Re**$_A$	0.72	-0.46	-0.42	0.80	0.36	1.00
^{5}**TS1**$_A$	0.87	-0.51	-0.46	0.93	0.17	1.00
^{5}**IM1**$_A$	0.86	-0.54	-0.47	1.02	0.13	1.00
^{5}**TS2**$_A$	0.83	-0.47	-0.52	1.13	0.04	1.00
^{5}**P**$_A$	0.81	-0.45	-0.62	1.30	-0.04	1.00
^{5}**Re**$_B$	0.76	-0.56	-0.42	0.84	1.38	2.00
^{5}**TS1**$_B$	0.92	-0.61	-0.45	0.96	1.18	2.00
^{5}**IM1**$_B$	0.92	-0.66	-0.44	1.04	1.15	2.00
^{5}**TS2**$_B$	0.84	-0.55	-0.52	1.27	0.95	2.00
^{5}**P**$_B$	0.75	-0.38	-0.57	1.22	0.98	2.00
**Spin**	**ρ(Fe)**	**ρ(O)**	**ρ(Succ)**	**ρ(Sub)**	**ρ(Prot)**	**ρ(Total)**
^{5}**Re**$_A$	3.15	0.60	0.13	0.00	0.11	4.00
^{5}**TS1**$_A$	3.83	0.12	0.14	-0.27	0.17	4.00
^{5}**IM1**$_A$	4.21	0.25	0.18	-0.90	0.26	4.00
^{5}**TS2**$_A$	4.03	0.12	0.12	-0.45	0.18	4.00
^{5}**P**$_A$	3.81	0.00	0.07	0.02	0.10	4.00
^{5}**Re**$_B$	3.21	0.53	0.14	0.00	0.12	4.00
^{5}**TS1**$_B$	3.88	0.02	0.16	-0.25	0.19	4.00
^{5}**IM1**$_B$	4.24	0.20	0.20	-0.95	0.30	4.00
^{5}**TS2**$_B$	3.94	0.11	0.10	-0.30	0.16	4.00
^{5}**P**$_B$	3.80	0.00	0.07	0.00	0.14	4.00

Table S10: Absolute (in au) and relative (in kcal mol^{-1}) single point energies of UB3LYP/BS1 optimized reactant complex as calculated in the quintet, singlet and triplet spin states with Gaussian-16 at UB3LYP/Def2TZVP, UB3LYP-GD3 /Def2TZVP, UPBE1PBE-GD3/Def2TZVP levels of theory.

Absolute	UB3LYP/ Def2TZVP	UB3LYP-GD3/ Def2TZVP	UPBE1PBE-GD3/ Def2TZVP
^{5}**Re**$_{A}$	-11515.34313	-11516.31937	-11504.31985
^{1}**Re**$_{A}$	-11515.30919	-11516.28543	-11504.27008
^{3}**Re**$_{A}$	-11515.32345	-11516.29973	-11504.29016
Relative	ΔE [BS2]	ΔE [BS2]	ΔE [BS2]
^{5}**Re**$_{A}$	0.00	0.00	0.00
^{1}**Re**$_{A}$	21.30	21.30	31.23
^{3}**Re**$_{A}$	12.35	12.32	18.63
Absolute	UB3LYP/ Def2TZVP	UB3LYP-GD3/ Def2TZVP	UPBE1PBE-GD3/ Def2TZVP
^{5}**Re**$_{B}$	-9972.005757	-9972.810644	-9962.618021
^{1}**Re**$_{B}$	-9971.967527	-9972.772413	-9962.569184
^{3}**Re**$_{B}$	-9971.981935	-9972.786822	-9962.584355
Relative	ΔE [BS2]	ΔE [BS2]	ΔE [BS2]
^{5}**Re**$_{B}$	0.00	0.00	0.00
^{1}**Re**$_{B}$	23.99	23.99	30.65
^{3}**Re**$_{B}$	14.95	14.95	21.13

Table S11: Absolute (in au) and relative (in kcal mol^{-1}) single point energies of UB3LYP/BS1 optimized first transition state complex as calculated in the quintet, singlet and triplet spin states with Gaussian-16 at UB3LYP/Def2TZVP, UB3LYP-GD3/Def2TZVP, UPBE1PBE-GD3/Def2TZVP levels of theory.

Absolute	UB3LYP/ Def2TZVP	UB3LYP-GD3/ Def2TZVP	UPBE1PBE-GD3/ Def2TZVP
^{5}**TS1**$_{A}$	-11515.313999	-11516.301293	-11504.305433
^{1}**TS1**$_{A}$	-11515.273602	-11516.260895	-11504.252216
^{3}**TS1**$_{A}$	-11515.281965	-11516.269258	-11504.256251
Relative	ΔE [BS2]	ΔE [BS2]	ΔE [BS2]
^{5}**TS1**$_{A}$	0.00	0.00	0.00
^{1}**TS1**$_{A}$	25.35	25.35	33.39
^{3}**TS1**$_{A}$	20.10	20.10	30.86
Absolute	UB3LYP/ Def2TZVP	UB3LYP-GD3/ Def2TZVP	UPBE1PBE-GD3/ Def2TZVP
^{5}**TS1**$_{B}$	-9971.953206	-9972.778680	-9962.588917
^{1}**TS1**$_{B}$	-9971.907836	-9972.734140	-9962.531087
^{3}**TS1**$_{B}$	-9971.914894	-9972.740368	-9962.546859
Relative	ΔE [BS2]	ΔE [BS2]	ΔE [BS2]
^{5}**TS1**$_{B}$	0.00	0.00	0.00
^{1}**TS1**$_{B}$	28.47	27.95	36.29
^{3}**TS1**$_{B}$	24.04	24.04	26.39

Table S12: Expectation value of the total spin-squared operator derived from the DFT calculations of single point energy at UB3LYP-GD3/BS2 level.

S**2	Before annihilation	After annihilation
^{5}**Re**$_A$	6.0592	6.0006
^{5}**TS1**$_A$	6.5001	6.0107
^{5}**IM1**$_A$	6.9719	6.0145
^{5}**TS2**$_A$	6.3927	6.0024
^{5}**P**$_A$	6.0090	6.0000
^{5}**Re**$_B$	6.0593	6.0006
^{5}**TS1**$_B$	6.5129	6.0110
^{5}**IM1**$_B$	7.0079	6.0143
^{5}**TS2**$_B$	6.2325	6.0011
^{5}**P**$_B$	6.0077	6.0000

Cartesian coordinates of optimized geometries:

^{5}Re$_{A}$

Element	X	Y	Z
C	9.227868	2.179873	5.709084
H	9.647493	1.766378	6.645142
C	7.710782	1.998137	5.689621
H	7.299283	2.319226	4.721247
H	7.244383	2.616934	6.475396
O	7.306769	0.628784	5.835888
H	7.526142	0.323799	6.728754
C	10.013794	1.579656	4.538643
O	11.060878	2.065461	4.147112
N	9.471808	0.459625	3.977101
H	8.637674	0.065115	4.404882
C	10.153958	-0.274496	2.937120
H	11.168494	-0.535491	3.291812
C	9.390900	-1.613019	2.687740
H	9.348089	-2.096972	3.687204
C	7.970287	-1.433138	2.167015
H	7.981818	-0.935091	1.186916
H	7.340063	-0.852990	2.858020
H	7.518670	-2.424967	2.026210
O	10.057770	-2.442249	1.771482
H	11.019392	-2.492466	1.918813
C	10.352793	0.585677	1.671426
O	9.566367	1.470256	1.314259
N	11.455501	0.291948	0.966003
H	12.028810	-0.503225	1.249315
C	11.842076	0.936843	-0.275830
H	12.192715	1.960527	-0.051335
C	13.020839	0.161673	-0.920042
H	13.447262	0.792560	-1.714919
H	13.803773	0.053609	-0.152716
C	12.702304	-1.223346	-1.514814
H	13.651356	-1.660144	-1.870064
H	12.041676	-1.140138	-2.389123
C	12.118713	-2.198695	-0.497584
O	12.584930	-2.263530	0.648732
N	11.098200	-2.971680	-0.907904
H	10.582109	-3.456562	-0.178035
H	10.553256	-2.735277	-1.730645
C	10.671380	1.024994	-1.269038
O	9.925839	0.065275	-1.446614

N	10.610929	2.165080	-1.993070
H	11.238039	2.921621	-1.741587
C	9.678380	2.387524	-3.092249
H	9.190254	1.423183	-3.291134
C	8.631049	3.469510	-2.807142
H	9.145288	4.417160	-2.560198
H	8.084092	3.658606	-3.747265
C	7.645405	3.101440	-1.696821
H	7.088287	2.194717	-1.983490
H	8.197070	2.865987	-0.770907
C	6.641277	4.218415	-1.413592
H	7.172585	5.171385	-1.217780
H	6.021827	4.376271	-2.313511
N	5.779842	3.863314	-0.293091
H	6.223120	3.308499	0.445148
C	4.703711	4.599078	0.081526
N	4.263093	5.601056	-0.673364
H	3.414764	6.116769	-0.374630
H	4.602445	5.735239	-1.614558
N	4.105153	4.331818	1.244288
H	3.168071	4.704167	1.417383
H	4.336465	3.471663	1.760500
C	7.374806	-4.447247	-5.533868
H	8.390285	-4.227074	-5.164919
C	6.511994	-3.172253	-5.433155
H	7.092720	-2.352295	-5.898238
C	5.177003	-3.291599	-6.155121
H	4.590579	-2.368449	-6.029407
H	4.599879	-4.147465	-5.774485
H	5.336680	-3.445369	-7.233003
O	6.244316	-2.817872	-4.071883
H	6.988131	-3.016542	-3.465441
C	6.786359	-5.634055	-4.774597
O	5.906958	-6.350564	-5.227891
N	7.316273	-5.810259	-3.529759
H	7.863015	-5.040146	-3.152891
C	6.730706	-6.737683	-2.587665
H	6.757170	-7.740868	-3.043917
C	7.530920	-6.775965	-1.245707
H	6.817791	-6.899917	-0.416965
H	8.168348	-7.673789	-1.243297
C	8.438529	-5.567646	-0.938993
H	9.285625	-5.536460	-1.640795
H	8.852531	-5.722662	0.070184

C	7.724563	-4.207573	-0.933477
O	7.998633	-3.406942	-1.898677
O	6.933431	-3.985142	0.002244
C	5.246904	-6.414581	-2.298752
O	4.850153	-5.259085	-2.165656
N	4.472171	-7.501745	-2.099620
H	4.854415	-8.403819	-2.359585
C	3.081936	-7.452658	-1.670959
H	2.954279	-6.543628	-1.066213
C	2.051459	-7.462677	-2.817502
H	2.189955	-6.553157	-3.423200
H	2.254357	-8.324815	-3.474881
C	0.642575	-7.541585	-2.273351
C	-0.021146	-8.773047	-2.142304
H	0.456861	-9.684490	-2.515346
C	-1.278430	-8.865884	-1.548235
H	-1.789931	-9.826026	-1.452296
C	-1.912740	-7.714945	-1.051445
O	-3.116859	-7.853537	-0.459735
H	-3.450215	-6.995475	-0.099380
C	-1.269648	-6.471744	-1.190985
H	-1.761397	-5.571143	-0.815733
C	-0.012312	-6.396150	-1.794042
H	0.473680	-5.421389	-1.896763
C	-2.718614	1.269944	-4.771042
H	-1.976981	1.550612	-4.000361
C	-2.326088	1.901700	-6.127202
H	-3.186673	1.781619	-6.812966
C	-2.026509	3.401925	-6.002429
H	-2.880890	3.915954	-5.541992
H	-1.135288	3.580346	-5.376346
H	-1.843198	3.858468	-6.987882
C	-1.144698	1.118910	-6.735498
H	-1.423885	0.050099	-6.775480
H	-0.284029	1.181799	-6.044028
C	-0.714762	1.564037	-8.134844
H	-0.315053	2.589877	-8.139324
H	-1.560193	1.534792	-8.842890
H	0.074054	0.906322	-8.534382
C	-4.104248	1.656774	-4.268977
O	-4.525457	2.815345	-4.268233
N	-4.863396	0.625640	-3.813214
H	-4.479816	-0.322178	-3.784876
C	-6.226323	0.838566	-3.377798

H	-6.806529	1.263605	-4.217236
C	-6.869908	-0.485329	-2.946814
H	-6.343825	-0.911535	-2.080826
H	-6.822644	-1.211472	-3.771607
H	-7.924962	-0.332326	-2.678920
C	-6.302165	1.881229	-2.247215
O	-5.358494	2.128455	-1.492637
N	-7.503013	2.477485	-2.107523
H	-8.268378	2.310594	-2.771026
C	-7.730726	3.636704	-1.260608
H	-7.393770	4.543308	-1.799070
C	-9.231708	3.782755	-0.953358
H	-9.560411	2.875233	-0.422479
H	-9.384469	4.634851	-0.277635
C	-10.072445	3.925668	-2.220011
O	-9.993660	3.094227	-3.122806
N	-10.895530	4.993086	-2.260317
H	-10.813581	5.763933	-1.594276
H	-11.447764	5.127891	-3.101256
C	-6.961291	3.562174	0.071272
O	-7.233091	2.742060	0.937851
N	-6.056789	4.559209	0.248670
C	-5.201279	4.644345	1.406190
C	-4.793787	6.106051	1.616462
C	-3.931999	3.731255	1.312384
O	-4.816471	6.932214	0.706803
C	-2.968770	4.074436	0.218562
C	-1.848398	4.879138	0.208407
N	-3.086849	3.599904	-1.073900
C	-2.076361	4.102002	-1.805735
N	-1.316234	4.893825	-1.060034
H	-5.842587	5.180046	-0.523816
H	-5.785113	4.297407	2.273541
H	-3.397359	3.778196	2.273419
H	-4.292157	2.698404	1.193286
H	-1.384244	5.418223	1.029388
H	-1.909121	3.870782	-2.853542
H	-3.837146	2.967752	-1.404334
N	-4.364687	6.385118	2.873244
H	-4.243761	5.599899	3.507766
C	-3.510939	7.508654	3.184750
H	-3.480904	8.174535	2.311143
C	-2.113878	6.970739	3.545744
O	-1.996294	5.811951	3.942706

N	-1.086010	7.820824	3.386898
C	0.319672	7.432463	3.536648
C	1.253819	8.511741	2.987534
C	1.021456	8.864591	1.512764
C	1.334567	7.706472	0.554171
O	2.409841	7.120721	0.677063
O	0.418069	7.479040	-0.326009
H	-1.286052	8.754667	3.046392
H	0.476790	6.480776	3.001914
H	1.168795	9.424431	3.603582
H	2.286195	8.147522	3.098406
H	1.696476	9.692096	1.236128
H	-0.004128	9.221014	1.329247
C	-2.899911	0.655479	6.393482
H	-3.725054	1.312246	6.067741
C	-1.579885	1.075035	5.736876
H	-1.731036	1.130448	4.646677
H	-0.838701	0.278954	5.901949
C	-1.040201	2.415298	6.264659
H	-0.759693	2.311989	7.326469
H	-1.807505	3.204330	6.199279
C	0.199984	2.814705	5.486272
O	1.219481	2.101475	5.502217
N	0.130210	3.924429	4.739815
H	-0.722754	4.473347	4.642941
H	0.845497	4.077095	4.018016
C	-3.264902	-0.772378	6.008982
O	-3.298499	-1.152467	4.845959
N	-3.563377	-1.600081	7.055721
H	-3.402922	-1.241867	7.988539
C	-3.560266	-3.041081	6.897751
H	-2.605340	-3.369608	6.456789
C	-4.673453	-3.635468	6.039789
O	-4.577806	-4.804362	5.660413
N	-5.719468	-2.843198	5.728206
H	-5.705729	-1.848377	5.962665
C	-6.766401	-3.300125	4.829833
H	-7.125705	-4.281187	5.179094
C	-7.952378	-2.314175	4.814277
H	-8.728938	-2.748878	4.170362
H	-8.362712	-2.248292	5.835700
C	-7.575357	-0.952219	4.315225
N	-6.715776	-0.137243	5.022872
C	-6.547037	0.935308	4.274317

H	-5.923703	1.793453	4.525067
N	-7.252953	0.862209	3.116371
H	-7.266254	1.552871	2.360323
C	-7.915048	-0.341663	3.124535
H	-8.551592	-0.662632	2.304183
C	-6.285310	-3.579326	3.392079
O	-7.011007	-4.198042	2.623651
N	-5.041767	-3.155668	3.042676
H	-4.484698	-2.575705	3.672637
C	-4.492524	-3.553924	1.761454
H	-5.254060	-3.381334	0.986202
C	-3.208373	-2.784443	1.413551
H	-2.452826	-2.951501	2.198407
H	-2.808200	-3.234073	0.488781
C	-3.377968	-1.270166	1.187050
H	-3.729498	-0.822917	2.133598
C	-2.009376	-0.652036	0.872341
H	-1.603253	-1.059665	-0.070722
H	-2.080124	0.440765	0.748360
H	-1.284415	-0.865919	1.671787
C	-4.394149	-0.951319	0.084679
H	-4.443272	0.128451	-0.123930
H	-4.121724	-1.450321	-0.862075
H	-5.412495	-1.272794	0.357195
C	-4.230210	-5.067188	1.672693
O	-4.155671	-5.579315	0.534533
N	-4.059432	-5.747755	2.806702
H	-4.223745	-5.264262	3.694718
C	-3.873017	-7.194270	2.874182
H	-3.348886	-7.525162	1.968070
C	-3.103658	-7.619807	4.133565
H	-3.114256	-8.722067	4.156063
H	-3.656784	-7.277358	5.022027
C	-1.674243	-7.121986	4.201438
C	-1.330389	-6.043242	5.030297
H	-2.113350	-5.570606	5.628045
C	-0.014006	-5.569504	5.094287
H	0.216492	-4.711388	5.731865
C	0.981649	-6.175133	4.319232
H	2.013121	-5.815044	4.368883
C	0.652095	-7.250826	3.485695
H	1.424884	-7.732146	2.880371
C	-0.663321	-7.717722	3.427972
H	-0.907804	-8.560471	2.774263

C	-7.713814	8.349711	-0.513272
H	-6.868259	8.303273	0.188042
C	-8.896693	7.535893	0.043567
H	-9.230681	7.985743	0.993969
H	-8.529463	6.517859	0.284955
O	-9.994782	7.477418	-0.842694
H	-9.639860	7.670921	-1.738614
C	-7.338468	7.855824	-1.899788
O	-8.188358	7.857601	-2.793601
N	-6.074969	7.389011	-2.094641
C	-5.644923	6.884033	-3.394808
C	-4.615367	7.786736	-4.107570
C	-3.296991	7.844404	-3.404352
C	-2.220007	6.991321	-3.446852
N	-2.989288	8.747754	-2.398874
C	-1.783327	8.419106	-1.876086
N	-1.299716	7.352966	-2.486401
H	-5.446008	7.351618	-1.291623
H	-6.549907	6.808202	-4.012164
H	-5.038940	8.796663	-4.237020
H	-4.455667	7.379166	-5.117746
H	-2.054287	6.136318	-4.095891
H	-1.291155	8.943375	-1.062345
H	-3.563133	9.532503	-2.112425
Fe	0.276902	6.102068	-1.755366
O	1.409232	5.097271	-1.211501
O	1.565109	6.902217	-3.248047
C	1.107151	6.090259	-4.102704
O	0.198936	5.277589	-3.755668
C	1.614820	6.097976	-5.522910
H	2.483000	6.760657	-5.626861
H	1.873942	5.073800	-5.829382
C	-2.424850	-3.334833	-5.271610
C	-3.510885	-3.270306	-4.199973
C	-1.134525	-2.697377	-4.732565
O	-3.856091	-2.195937	-3.699103
C	0.035016	-2.761887	-5.715726
S	1.435153	-1.686441	-5.207089
N	-4.005226	-4.457163	-3.804581
C	-4.977681	-4.625115	-2.747495
C	-5.462184	-6.079766	-2.779950
O	-5.412599	-6.723680	-3.816836
N	-5.958145	-6.540002	-1.599455
C	-6.357498	-7.928554	-1.436435

C	-7.733449	-8.160858	-0.803683
O	-8.035058	-9.271514	-0.400643
N	-8.563351	-7.081320	-0.769944
C	-9.929026	-7.148439	-0.278371
C	-10.189799	-6.384350	1.035514
C	-9.368923	-6.942845	2.203545
C	-9.985836	-4.871677	0.876375
C	2.075456	-2.605053	-3.824003
O	1.781250	-3.748249	-3.563202
C	3.004483	-1.790831	-2.911271
N	4.065034	-2.695562	-2.414412
C	2.204520	-1.199043	-1.723086
N	1.214413	-0.248913	-2.184990
C	0.712447	0.865806	-1.558953
O	-0.070394	1.603111	-2.142787
C	1.139117	1.110293	-0.145670
C	1.031382	2.334482	0.395147
C	1.335748	2.683783	1.816651
O	1.417135	3.882105	2.153874
N	1.508567	1.682338	2.685851
H	-2.780926	-2.774682	-6.153169
H	-2.246284	-4.374483	-5.588452
H	-0.843858	-3.206621	-3.800310
H	-1.349324	-1.650110	-4.473111
H	0.426737	-3.782917	-5.832935
H	-0.239945	-2.379976	-6.711031
H	-3.816254	-5.301808	-4.341002
H	-4.548970	-4.378945	-1.762481
H	-5.645394	-6.049251	-0.758928
H	-6.364046	-8.392874	-2.433880
H	-8.205974	-6.209047	-1.140841
H	-10.149041	-8.216695	-0.139437
H	-11.260552	-6.557964	1.254307
H	3.493004	-0.957747	-3.437327
H	1.753570	-2.033960	-1.152374
H	2.931331	-0.700957	-1.065910
H	0.830511	-0.378247	-3.118992
H	1.491366	0.258458	0.442687
H	0.711819	3.178124	-0.221564
H	1.685237	1.906383	3.669970
H	1.384531	0.689082	2.463320
H	4.846106	-2.789606	-3.116960
H	4.516040	-2.356938	-1.517346
H	3.751813	-3.670576	-2.252681

H	-8.922787	-4.624004	0.724604
H	-10.571008	-4.470560	0.030919
H	-10.301328	-4.335672	1.785855
H	-8.294984	-6.746642	2.060162
H	-9.508754	-8.029963	2.310310
H	-9.658812	-6.461184	3.151143
H	10.254192	2.660203	-3.992640
H	9.472099	3.253038	5.725584
H	2.901293	-8.313381	-1.007101
H	7.461874	-4.731677	-6.592848
H	-4.860687	-7.689950	2.871802
H	-3.633533	-3.504643	7.893168
H	-5.844323	-3.958067	-2.902862
H	-10.610660	-6.766907	-1.060425
H	-5.618590	-8.479336	-0.834268
H	0.541763	7.240523	4.599591
H	0.810522	6.444155	-6.191107
H	-5.224285	5.870670	-3.285549
H	-8.021122	9.403053	-0.625037
H	-3.908524	8.084405	4.039410
H	-2.674963	0.172733	-4.846773
H	-2.830221	0.753504	7.489654
O	-0.416337	-2.882787	3.363070
H	-0.169285	-3.819387	3.386722
H	0.348220	-2.408583	2.967700
O	1.618573	-1.168056	2.737636
H	1.747542	-0.973123	3.703004
H	2.533403	-1.189756	2.378618
O	4.239095	-0.731503	1.975690
H	4.639939	-0.907518	2.850207
H	4.714850	-1.265147	1.282452
O	5.385072	-1.931672	-0.107112
H	5.947030	-2.777094	0.001166
H	6.062329	-1.312778	-0.473983
O	7.360889	-0.828040	-1.544440
H	8.223356	-0.372073	-1.449205
H	7.612106	-1.783392	-1.702067
O	5.200855	0.494045	-2.621489
H	4.998444	1.001356	-1.824103
H	6.018462	0.001917	-2.382387
O	4.825390	1.869583	2.599813
H	5.787502	1.987444	2.440496
H	4.569808	1.034395	2.138589
O	7.355612	2.673411	1.992982

H	7.687092	3.492892	2.385767
H	8.176713	2.130964	1.807810
O	2.100595	-0.509356	5.348790
H	3.073372	-0.390644	5.314081
H	1.740581	0.382616	5.555098
O	4.798020	-0.037196	4.615559
H	5.611104	0.073332	5.144733
H	4.726625	0.805092	4.110459
O	-0.264989	-2.117659	6.076963
H	0.634243	-1.746926	6.048285
H	-0.442113	-2.327070	5.133189

^{5}**TS1**$_{A}$

Element	X	Y	Z
C	9.323554	2.105767	5.792586
H	9.745201	1.675189	6.720046
C	7.806047	1.928798	5.774971
H	7.391701	2.272081	4.815378
H	7.344907	2.533094	6.575112
O	7.398690	0.557910	5.895478
H	7.633645	0.231826	6.776863
C	10.105661	1.524138	4.610441
O	11.161000	2.004962	4.235492
N	9.550904	0.426151	4.018255
H	8.713914	0.027610	4.436771
C	10.228661	-0.288829	2.962172
H	11.237120	-0.575450	3.314712
C	9.446927	-1.607621	2.669731
H	9.386331	-2.117390	3.655333
C	8.034791	-1.391263	2.140090
H	8.064068	-0.866138	1.174560
H	7.406205	-0.820908	2.840586
H	7.569971	-2.371678	1.966700
O	10.109701	-2.423304	1.738306
H	11.069315	-2.490321	1.891354
C	10.447199	0.603734	1.722808
O	9.675704	1.508946	1.385946
N	11.548660	0.314745	1.013555
H	12.110554	-0.495648	1.275613
C	11.940454	0.985813	-0.212444
H	12.296079	2.002375	0.035190
C	13.115215	0.218349	-0.873306
H	13.550201	0.866398	-1.649489

H	13.894151	0.083218	-0.106215
C	12.786987	-1.147630	-1.506126
H	13.734492	-1.586103	-1.863251
H	12.136192	-1.034794	-2.384551
C	12.182329	-2.141251	-0.519212
O	12.643545	-2.247056	0.625874
N	11.148378	-2.882704	-0.954297
H	10.619325	-3.374459	-0.238228
H	10.610844	-2.608683	-1.770250
C	10.771382	1.101681	-1.204609
O	10.016653	0.152171	-1.398467
N	10.722985	2.252093	-1.913637
H	11.350872	3.003013	-1.647987
C	9.804726	2.482651	-3.023100
H	9.368480	1.506004	-3.275822
C	8.692926	3.494830	-2.726104
H	9.140875	4.474702	-2.477532
H	8.132632	3.647893	-3.665268
C	7.735556	3.058595	-1.615277
H	7.334141	2.060688	-1.846846
H	8.281801	2.970845	-0.661413
C	6.563695	4.023237	-1.448127
H	6.931643	5.062287	-1.340579
H	5.945267	3.992917	-2.361992
N	5.740459	3.654349	-0.296134
H	6.244537	3.179927	0.459594
C	4.708652	4.443254	0.111837
N	4.235027	5.403828	-0.673444
H	3.436500	5.953761	-0.307654
H	4.388875	5.405692	-1.672630
N	4.200498	4.284663	1.335945
H	3.318191	4.755980	1.546592
H	4.443169	3.458231	1.901772
C	7.333723	-4.178116	-5.659932
H	8.358151	-3.976351	-5.305473
C	6.486848	-2.896993	-5.514838
H	7.066012	-2.074420	-5.977412
C	5.134392	-2.988242	-6.208393
H	4.558693	-2.063769	-6.046673
H	4.559046	-3.848438	-5.835031
H	5.267489	-3.117230	-7.293097
O	6.253836	-2.565355	-4.141604
H	7.002960	-2.795627	-3.553004
C	6.747656	-5.377533	-4.918392

O	5.850434	-6.071738	-5.371479
N	7.302846	-5.593498	-3.691005
H	7.865399	-4.839390	-3.305155
C	6.728398	-6.540497	-2.761654
H	6.734449	-7.531215	-3.245154
C	7.557329	-6.623153	-1.439739
H	6.861658	-6.776651	-0.601172
H	8.195339	-7.519645	-1.481360
C	8.470871	-5.424793	-1.112057
H	9.308239	-5.375547	-1.824398
H	8.898694	-5.608813	-0.113573
C	7.761023	-4.063349	-1.059440
O	8.022864	-3.239060	-2.007746
O	6.985920	-3.862692	-0.105545
C	5.254250	-6.211499	-2.431755
O	4.869474	-5.055547	-2.272748
N	4.477908	-7.297401	-2.230954
H	4.851437	-8.197483	-2.510093
C	3.096281	-7.252424	-1.774505
H	2.974322	-6.336164	-1.179927
C	2.046644	-7.286434	-2.902952
H	2.158512	-6.376062	-3.512924
H	2.256973	-8.146051	-3.561370
C	0.646584	-7.394266	-2.340724
C	0.002608	-8.637387	-2.221884
H	0.491107	-9.536113	-2.611777
C	-1.248840	-8.757771	-1.620286
H	-1.745120	-9.726860	-1.534923
C	-1.897579	-7.623571	-1.104078
O	-3.097276	-7.787973	-0.510090
H	-3.444240	-6.939199	-0.140819
C	-1.273350	-6.369100	-1.229076
H	-1.775994	-5.481558	-0.837556
C	-0.021193	-6.265748	-1.838991
H	0.450474	-5.282829	-1.928830
C	-3.019390	1.112047	-4.669651
H	-2.274576	1.337861	-3.883492
C	-2.596730	1.772171	-6.002416
H	-3.477294	1.767019	-6.672382
C	-2.163548	3.231051	-5.805089
H	-2.966414	3.789486	-5.305011
H	-1.252130	3.291594	-5.184708
H	-1.952457	3.725342	-6.766461
C	-1.503130	0.923660	-6.681935

H	-1.877959	-0.112283	-6.771138
H	-0.623891	0.873031	-6.012754
C	-1.067178	1.406177	-8.067096
H	-0.577954	2.391808	-8.028851
H	-1.928121	1.489691	-8.751611
H	-0.349739	0.705028	-8.523272
C	-4.391335	1.541235	-4.162699
O	-4.785363	2.709150	-4.178404
N	-5.168328	0.533517	-3.685466
H	-4.798591	-0.419437	-3.658982
C	-6.526613	0.771386	-3.244749
H	-7.117959	1.157385	-4.095280
C	-7.164590	-0.530036	-2.743633
H	-6.627947	-0.914144	-1.864338
H	-7.129396	-1.294531	-3.534154
H	-8.216208	-0.363973	-2.469822
C	-6.581475	1.861992	-2.159980
O	-5.643152	2.090779	-1.392788
N	-7.751487	2.522430	-2.067555
H	-8.513217	2.369418	-2.738877
C	-7.916698	3.734028	-1.281228
H	-7.483171	4.582543	-1.844317
C	-9.412071	4.017676	-1.049824
H	-9.835808	3.174109	-0.482095
H	-9.519753	4.921203	-0.434448
C	-10.186435	4.143362	-2.360856
O	-10.168028	3.232637	-3.187228
N	-10.882183	5.286162	-2.528735
H	-10.728926	6.105560	-1.936733
H	-11.377204	5.405065	-3.407009
C	-7.207786	3.658078	0.084790
O	-7.540775	2.860363	0.951296
N	-6.276045	4.627001	0.279994
C	-5.462997	4.711705	1.468936
C	-4.947476	6.147982	1.613424
C	-4.258604	3.712085	1.463890
O	-4.885581	6.918626	0.658808
C	-3.242035	3.957890	0.393684
C	-2.056744	4.663724	0.403786
N	-3.377169	3.491640	-0.899794
C	-2.311160	3.899630	-1.610184
N	-1.497018	4.623846	-0.853963
H	-6.010599	5.228748	-0.492576
H	-6.099650	4.456925	2.331538

H	-3.754599	3.763379	2.440894
H	-4.681044	2.700431	1.368463
H	-1.561634	5.158725	1.234916
H	-2.141687	3.634253	-2.649082
H	-4.161689	2.906305	-1.241027
N	-4.522460	6.464190	2.863253
H	-4.473448	5.709616	3.542070
C	-3.610647	7.551490	3.132541
H	-3.537408	8.171187	2.227905
C	-2.240063	6.972543	3.533077
O	-2.157951	5.806130	3.915532
N	-1.191334	7.807325	3.427016
C	0.200838	7.397306	3.633186
C	1.171295	8.402518	3.012577
C	1.001564	8.590043	1.499866
C	1.412277	7.358392	0.682123
O	2.537447	6.884775	0.856482
O	0.534292	6.951797	-0.164532
H	-1.365129	8.758143	3.119578
H	0.337141	6.405041	3.175274
H	1.069775	9.378098	3.521191
H	2.195836	8.051057	3.208026
H	1.655057	9.414583	1.168719
H	-0.030732	8.869949	1.240071
C	-2.871127	0.624027	6.411700
H	-3.708112	1.287443	6.131809
C	-1.573733	1.067068	5.726800
H	-1.762195	1.172585	4.646166
H	-0.830114	0.263360	5.832545
C	-1.018488	2.381289	6.300742
H	-0.683570	2.221737	7.339389
H	-1.796941	3.161923	6.320496
C	0.174812	2.849623	5.489562
O	1.224527	2.183820	5.443983
N	0.028890	3.980944	4.786858
H	-0.854635	4.483577	4.721925
H	0.717241	4.195581	4.055666
C	-3.249859	-0.793710	6.006093
O	-3.285721	-1.160490	4.839234
N	-3.564520	-1.629607	7.042107
H	-3.403845	-1.286476	7.980513
C	-3.589176	-3.067006	6.862681
H	-2.644683	-3.406652	6.407362
C	-4.717361	-3.629114	6.002564

O	-4.647484	-4.797216	5.615629
N	-5.745362	-2.811252	5.695459
H	-5.708656	-1.817487	5.930684
C	-6.801023	-3.243564	4.794636
H	-7.172484	-4.222222	5.137659
C	-7.976258	-2.245690	4.790478
H	-8.760860	-2.671076	4.150022
H	-8.380249	-2.184180	5.815033
C	-7.602233	-0.880198	4.297006
N	-6.705241	-0.084104	4.979365
C	-6.575158	1.008292	4.252029
H	-5.937752	1.859036	4.492603
N	-7.341714	0.966145	3.130912
H	-7.407588	1.679985	2.398492
C	-8.003316	-0.238169	3.142383
H	-8.684627	-0.535382	2.349533
C	-6.324159	-3.522597	3.355564
O	-7.059028	-4.125091	2.583191
N	-5.072054	-3.119667	3.012342
H	-4.507680	-2.552431	3.647115
C	-4.522758	-3.522508	1.733008
H	-5.284861	-3.350664	0.958434
C	-3.240840	-2.751040	1.380926
H	-2.479433	-2.916258	2.160357
H	-2.845498	-3.196863	0.452448
C	-3.416601	-1.236228	1.161240
H	-3.733813	-0.790634	2.120709
C	-2.061026	-0.617809	0.795467
H	-1.702684	-1.006010	-0.174053
H	-2.135844	0.476810	0.692680
H	-1.301206	-0.849858	1.556158
C	-4.472294	-0.917058	0.097098
H	-4.520419	0.160919	-0.118741
H	-4.240709	-1.425652	-0.855341
H	-5.482181	-1.226718	0.411501
C	-4.262085	-5.035876	1.645178
O	-4.176336	-5.544597	0.506264
N	-4.108485	-5.720457	2.778968
H	-4.281358	-5.240623	3.667463
C	-3.905152	-7.164264	2.846295
H	-3.416676	-7.492998	1.919968
C	-3.074540	-7.573401	4.072160
H	-3.056885	-8.675676	4.093155
H	-3.598350	-7.244991	4.983466

C	-1.657774	-7.037282	4.078764
C	-1.306616	-5.955940	4.900644
H	-2.073329	-5.512676	5.540520
C	-0.004397	-5.441548	4.906253
H	0.230631	-4.582637	5.541080
C	0.970047	-6.009792	4.077849
H	1.991844	-5.619589	4.082357
C	0.633340	-7.087799	3.249825
H	1.389699	-7.539353	2.602333
C	-0.668196	-7.595074	3.251186
H	-0.918383	-8.438768	2.600933
C	-7.413413	8.465652	-0.977013
H	-6.619055	8.417400	-0.218759
C	-8.698046	7.823779	-0.422062
H	-9.035481	8.393305	0.460446
H	-8.447600	6.803970	-0.065295
O	-9.748831	7.784882	-1.364914
H	-9.326479	7.832500	-2.251440
C	-7.006597	7.798629	-2.280326
O	-7.805607	7.767139	-3.219372
N	-5.774113	7.224702	-2.351499
C	-5.312010	6.557684	-3.567017
C	-4.193752	7.315534	-4.316723
C	-2.902283	7.375168	-3.564846
C	-1.830843	6.512706	-3.533437
N	-2.630587	8.302588	-2.570338
C	-1.453315	7.970347	-1.983658
N	-0.952174	6.883370	-2.538426
H	-5.199934	7.216999	-1.509003
H	-6.188578	6.471127	-4.222539
H	-4.548690	8.327385	-4.576264
H	-4.016583	6.789687	-5.267345
H	-1.649802	5.636497	-4.149110
H	-1.005443	8.509249	-1.153881
H	-3.204210	9.104783	-2.336904
Fe	0.412014	5.436423	-1.446687
O	1.247577	4.198016	-0.629260
O	2.057842	5.891456	-2.749593
C	1.566326	5.142558	-3.649049
O	0.470829	4.551810	-3.429876
C	2.284937	4.942821	-4.960417
H	3.126853	5.639385	-5.061760
H	2.654132	3.905940	-5.011803
C	-2.476716	-3.361598	-5.178359

C	-3.598452	-3.310024	-4.143214
C	-1.220523	-2.668979	-4.626345
O	-3.988253	-2.238271	-3.670269
C	-0.037573	-2.708343	-5.595389
S	1.311267	-1.556970	-5.118057
N	-4.079887	-4.503357	-3.751764
C	-5.082674	-4.678431	-2.724404
C	-5.526199	-6.145736	-2.743012
O	-5.454244	-6.800619	-3.771305
N	-6.013831	-6.605583	-1.558209
C	-6.366804	-8.004368	-1.378442
C	-7.713059	-8.269860	-0.697377
O	-7.963725	-9.380712	-0.261254
N	-8.578397	-7.218405	-0.661350
C	-9.924757	-7.319728	-0.124452
C	-10.173164	-6.521121	1.171114
C	-9.292981	-7.006446	2.328641
C	-10.035537	-5.008073	0.954854
C	2.039226	-2.429762	-3.753253
O	1.779786	-3.571571	-3.449177
C	3.015433	-1.584094	-2.918890
N	4.099560	-2.478150	-2.451451
C	2.298404	-0.945465	-1.704220
N	1.237847	-0.057473	-2.124297
C	0.677235	1.012734	-1.462494
O	-0.399256	1.464502	-1.840624
C	1.401372	1.584186	-0.302989
C	0.800452	2.572408	0.442954
C	1.280643	2.974076	1.817456
O	1.260505	4.148791	2.210885
N	1.669544	1.952954	2.592831
H	-2.826033	-2.834933	-6.083207
H	-2.255599	-4.401679	-5.465668
H	-0.925188	-3.152312	-3.681589
H	-1.477360	-1.626004	-4.389148
H	0.399681	-3.714054	-5.679335
H	-0.322160	-2.369713	-6.603528
H	-3.847903	-5.349712	-4.267560
H	-4.691405	-4.401451	-1.732305
H	-5.709614	-6.098244	-0.724941
H	-6.393400	-8.471911	-2.374129
H	-8.263313	-6.343594	-1.063559
H	-10.098914	-8.390171	0.056317
H	-11.228291	-6.728800	1.432016

H	3.466967	-0.771607	-3.504913
H	1.930925	-1.755586	-1.044093
H	3.066095	-0.385818	-1.155345
H	0.627519	-0.386268	-2.869019
H	2.407738	1.244116	-0.053006
H	-0.253394	2.770739	0.245855
H	1.841126	2.141642	3.586796
H	1.550331	0.961732	2.343222
H	4.871251	-2.554576	-3.165632
H	4.557570	-2.154781	-1.553329
H	3.796864	-3.459070	-2.300304
H	-8.989210	-4.723674	0.758218
H	-10.664350	-4.659838	0.117387
H	-10.341309	-4.453427	1.856333
H	-8.232906	-6.773682	2.142357
H	-9.385480	-8.093880	2.476330
H	-9.571072	-6.504118	3.268997
H	10.388578	2.822194	-3.894844
H	9.570951	3.177788	5.827347
H	2.934466	-8.105905	-1.096351
H	7.398285	-4.438717	-6.726663
H	-4.886306	-7.670594	2.890873
H	-3.663406	-3.545838	7.850698
H	-5.961706	-4.039254	-2.922240
H	-10.644816	-6.990939	-0.895995
H	-5.593033	-8.532945	-0.800384
H	0.404787	7.286928	4.711793
H	1.580559	5.077984	-5.794186
H	-4.970262	5.537336	-3.330355
H	-7.613196	9.524541	-1.212862
H	-3.986611	8.189994	3.951810
H	-3.022830	0.017091	-4.774730
H	-2.766145	0.693055	7.507294
O	-0.331459	-2.681347	3.223099
H	-0.066376	-3.611942	3.178924
H	0.418940	-2.163890	2.854316
O	1.670383	-0.893817	2.654367
H	1.794524	-0.755408	3.630585
H	2.587989	-0.970300	2.312507
O	4.349510	-0.635187	1.992938
H	4.715383	-0.887349	2.863511
H	4.804089	-1.160166	1.277970
O	5.451813	-1.794303	-0.131623
H	6.008731	-2.646558	-0.058144

H	6.126208	-1.168424	-0.490221
O	7.420216	-0.665394	-1.574100
H	8.295014	-0.239739	-1.454579
H	7.648459	-1.620691	-1.764141
O	5.181776	0.712198	-2.439849
H	5.110785	1.273017	-1.654892
H	6.014939	0.211850	-2.293351
O	4.975028	1.902438	2.741338
H	5.933059	2.042931	2.569700
H	4.727178	1.080138	2.250549
O	7.459672	2.710892	2.034464
H	7.786012	3.550995	2.385961
H	8.284139	2.169013	1.863654
O	2.126811	-0.415306	5.306343
H	3.103986	-0.334281	5.314722
H	1.782547	0.483264	5.513050
O	4.866251	-0.062049	4.683710
H	5.676579	0.007714	5.223078
H	4.836349	0.794181	4.198646
O	-0.212672	-2.062000	5.977872
H	0.677788	-1.668564	5.954676
H	-0.392932	-2.232997	5.027177

^{5}**IM1**$_{A}$

Element	X	Y	Z
C	9.265627	2.075024	5.810917
H	9.666776	1.646180	6.748264
C	7.750402	1.885292	5.754168
H	7.356982	2.228918	4.786030
H	7.264795	2.483066	6.544719
O	7.350140	0.510970	5.859340
H	7.569863	0.182315	6.743781
C	10.085147	1.503087	4.649210
O	11.144894	1.995406	4.302941
N	9.558672	0.398588	4.043671
H	8.712264	-0.005385	4.437428
C	10.272273	-0.314353	3.010064
H	11.278914	-0.575432	3.386687
C	9.520863	-1.651836	2.722406
H	9.448796	-2.146918	3.714520
C	8.117561	-1.468069	2.159035
H	8.158673	-0.951533	1.189411
H	7.460015	-0.903966	2.837799

H	7.676189	-2.458805	1.984671
O	10.213523	-2.471927	1.817504
H	11.170101	-2.529863	1.989159
C	10.499969	0.567834	1.764080
O	9.733557	1.472897	1.415886
N	11.606441	0.273065	1.064163
H	12.165497	-0.536495	1.334236
C	12.010903	0.941902	-0.159318
H	12.361764	1.959602	0.090485
C	13.193766	0.176226	-0.808026
H	13.635849	0.824985	-1.579520
H	13.964852	0.040548	-0.033206
C	12.871138	-1.190112	-1.443148
H	13.821429	-1.629986	-1.790374
H	12.227828	-1.078438	-2.327377
C	12.257427	-2.176220	-0.454162
O	12.728592	-2.289969	0.685897
N	11.201942	-2.893272	-0.879195
H	10.663834	-3.363608	-0.154429
H	10.669278	-2.605582	-1.693958
C	10.853595	1.056127	-1.165471
O	10.084200	0.117099	-1.351291
N	10.833259	2.194426	-1.896254
H	11.471391	2.939353	-1.638207
C	9.930444	2.418481	-3.019450
H	9.489703	1.441918	-3.264794
C	8.821449	3.440397	-2.746204
H	9.270283	4.426040	-2.523799
H	8.258198	3.569709	-3.687244
C	7.869721	3.031583	-1.620458
H	7.485425	2.018703	-1.812063
H	8.417695	2.986660	-0.664545
C	6.682588	3.982070	-1.486601
H	7.034392	5.029944	-1.418435
H	6.060303	3.905773	-2.395186
N	5.870381	3.642392	-0.317701
H	6.382953	3.192820	0.449327
C	4.844045	4.445794	0.082385
N	4.365542	5.388053	-0.720339
H	3.577634	5.965972	-0.368782
H	4.539879	5.378752	-1.715821
N	4.352622	4.313139	1.315999
H	3.451733	4.753904	1.523302
H	4.575901	3.473248	1.870318

C	7.206324	-3.961580	-5.760117
H	8.244540	-3.777745	-5.437401
C	6.371216	-2.685772	-5.527604
H	6.935002	-1.846265	-5.979062
C	4.991611	-2.744471	-6.169019
H	4.424871	-1.828247	-5.942563
H	4.429123	-3.619447	-5.810800
H	5.080875	-2.825698	-7.262872
O	6.196214	-2.411318	-4.133316
H	6.966338	-2.674204	-3.587875
C	6.645568	-5.191393	-5.049049
O	5.727608	-5.862712	-5.495261
N	7.250836	-5.464052	-3.857312
H	7.827888	-4.727416	-3.458553
C	6.717490	-6.456982	-2.951707
H	6.699349	-7.421889	-3.484556
C	7.606868	-6.604660	-1.675542
H	6.951166	-6.796190	-0.812826
H	8.237729	-7.499857	-1.790104
C	8.540919	-5.425849	-1.334840
H	9.340023	-5.339873	-2.086453
H	9.020198	-5.664245	-0.371989
C	7.840327	-4.067533	-1.172688
O	8.046970	-3.197966	-2.093329
O	7.127708	-3.913050	-0.162848
C	5.261895	-6.144421	-2.537117
O	4.893941	-4.997579	-2.293313
N	4.486294	-7.235140	-2.363982
H	4.838943	-8.119836	-2.711317
C	3.125755	-7.207005	-1.845794
H	3.037121	-6.323390	-1.198251
C	2.029400	-7.173957	-2.928970
H	2.122072	-6.232833	-3.493408
H	2.207032	-7.997960	-3.640620
C	0.651060	-7.305285	-2.319405
C	0.027235	-8.558606	-2.197079
H	0.516213	-9.446042	-2.611537
C	-1.206121	-8.702728	-1.565090
H	-1.687745	-9.679165	-1.479913
C	-1.858293	-7.582930	-1.021976
O	-3.044177	-7.772429	-0.408090
H	-3.420290	-6.930591	-0.052042
C	-1.252663	-6.319414	-1.144776
H	-1.755963	-5.443059	-0.729069

C	-0.017235	-6.192366	-1.784997
H	0.440130	-5.202692	-1.875357
C	-3.119874	1.143956	-4.775263
H	-2.382028	1.349452	-3.976571
C	-2.677783	1.819801	-6.091449
H	-3.554022	1.839818	-6.766101
C	-2.227990	3.268649	-5.862741
H	-3.026324	3.825023	-5.353475
H	-1.317808	3.302267	-5.237921
H	-2.006235	3.781287	-6.811881
C	-1.589903	0.970317	-6.778615
H	-1.976213	-0.059196	-6.888719
H	-0.715739	0.898063	-6.104087
C	-1.138925	1.471966	-8.152053
H	-0.638379	2.450974	-8.093634
H	-1.994540	1.577664	-8.840017
H	-0.427330	0.770200	-8.616079
C	-4.493053	1.575783	-4.269120
O	-4.911063	2.731627	-4.344866
N	-5.238755	0.580476	-3.721957
H	-4.848416	-0.361894	-3.644946
C	-6.584862	0.829737	-3.255155
H	-7.179270	1.240855	-4.091186
C	-7.238671	-0.469065	-2.768386
H	-6.696723	-0.881213	-1.905461
H	-7.229895	-1.217583	-3.574687
H	-8.282728	-0.290519	-2.474392
C	-6.605601	1.911171	-2.158903
O	-5.629682	2.169853	-1.451119
N	-7.784586	2.541852	-1.994391
H	-8.578551	2.375207	-2.624139
C	-7.921631	3.754715	-1.205189
H	-7.505194	4.602271	-1.782287
C	-9.408530	4.039774	-0.924719
H	-9.810880	3.202036	-0.333297
H	-9.495607	4.950302	-0.316437
C	-10.231217	4.147939	-2.207848
O	-10.250226	3.221900	-3.016943
N	-10.926276	5.291906	-2.370145
H	-10.744081	6.121493	-1.800993
H	-11.452790	5.397994	-3.231637
C	-7.170494	3.677951	0.138217
O	-7.469921	2.873778	1.011089
N	-6.240293	4.652267	0.312098

C	-5.413686	4.749917	1.490988
C	-4.871841	6.180544	1.594425
C	-4.230114	3.726476	1.506239
O	-4.813570	6.928465	0.621602
C	-3.195117	3.930742	0.444929
C	-2.005202	4.627403	0.457801
N	-3.300410	3.413651	-0.831940
C	-2.213330	3.783033	-1.528546
N	-1.411577	4.532181	-0.781726
H	-5.993318	5.254343	-0.466502
H	-6.046254	4.528895	2.366173
H	-3.733903	3.779994	2.487212
H	-4.672738	2.722527	1.423829
H	-1.533013	5.153566	1.282898
H	-2.015950	3.479298	-2.551976
H	-4.089888	2.840595	-1.180837
N	-4.422402	6.521051	2.829111
H	-4.373550	5.783782	3.526432
C	-3.499124	7.608224	3.057117
H	-3.439401	8.206812	2.137374
C	-2.123781	7.032331	3.444392
O	-2.038653	5.869374	3.837495
N	-1.074284	7.862133	3.315324
C	0.318837	7.451545	3.516846
C	1.288160	8.459855	2.899286
C	1.119552	8.657271	1.386436
C	1.523427	7.425811	0.567352
O	2.675831	7.001814	0.673687
O	0.606645	6.953086	-0.203236
H	-1.248557	8.810482	3.000495
H	0.457146	6.459202	3.058719
H	1.186069	9.432819	3.412738
H	2.313035	8.108813	3.094258
H	1.777674	9.480453	1.062896
H	0.087550	8.942310	1.130656
C	-2.935169	0.638229	6.310734
H	-3.780136	1.286313	6.019421
C	-1.647634	1.068279	5.598614
H	-1.842733	1.127291	4.515541
H	-0.894333	0.277784	5.730793
C	-1.104068	2.409109	6.115511
H	-0.816923	2.307106	7.176389
H	-1.873616	3.196921	6.060286
C	0.135023	2.827984	5.344911

O	1.144110	2.101262	5.309175
N	0.087339	3.990054	4.682053
H	-0.761677	4.547210	4.600574
H	0.835150	4.196823	4.007933
C	-3.311907	-0.793572	5.955252
O	-3.375405	-1.192801	4.800115
N	-3.594825	-1.603655	7.020113
H	-3.415942	-1.236134	7.945867
C	-3.627054	-3.045254	6.875701
H	-2.698360	-3.396200	6.397143
C	-4.784057	-3.623864	6.064457
O	-4.728174	-4.798274	5.696228
N	-5.819317	-2.807603	5.775761
H	-5.771280	-1.811193	5.997298
C	-6.902072	-3.244488	4.909754
H	-7.279977	-4.210340	5.280957
C	-8.061974	-2.227907	4.911967
H	-8.865384	-2.651693	4.294225
H	-8.444395	-2.141843	5.942937
C	-7.675862	-0.876574	4.388750
N	-6.768294	-0.076397	5.052368
C	-6.627128	0.998715	4.301825
H	-5.980245	1.847545	4.522747
N	-7.396200	0.941862	3.183266
H	-7.448146	1.643148	2.438689
C	-8.071781	-0.254474	3.221263
H	-8.757749	-0.561523	2.436214
C	-6.465079	-3.555451	3.463888
O	-7.228650	-4.155746	2.718896
N	-5.216441	-3.172286	3.088034
H	-4.637044	-2.597903	3.702513
C	-4.695203	-3.570672	1.796486
H	-5.487434	-3.429960	1.045478
C	-3.454382	-2.751608	1.406056
H	-2.656183	-2.908994	2.149529
H	-3.082369	-3.162002	0.452238
C	-3.694064	-1.239971	1.237018
H	-4.018832	-0.838930	2.212670
C	-2.372284	-0.552510	0.878534
H	-2.015985	-0.872916	-0.114894
H	-2.488095	0.541879	0.829940
H	-1.589812	-0.791217	1.614077
C	-4.774303	-0.933657	0.193709
H	-4.872105	0.148944	0.019682

H	-4.522536	-1.394563	-0.777691
H	-5.765998	-1.303362	0.501178
C	-4.382889	-5.073958	1.708658
O	-4.252286	-5.574401	0.569760
N	-4.244023	-5.759728	2.843489
H	-4.432304	-5.280532	3.729182
C	-4.012462	-7.199013	2.916480
H	-3.542307	-7.525942	1.980479
C	-3.140964	-7.585024	4.121459
H	-3.110997	-8.686728	4.155118
H	-3.639446	-7.250508	5.044808
C	-1.731111	-7.033919	4.074713
C	-1.367382	-5.933218	4.864478
H	-2.118172	-5.487239	5.521279
C	-0.072477	-5.402865	4.817650
H	0.170583	-4.528588	5.427767
C	0.881470	-5.974784	3.968155
H	1.897802	-5.571963	3.931848
C	0.531922	-7.072162	3.171394
H	1.271918	-7.526237	2.507037
C	-0.762230	-7.595100	3.225113
H	-1.023188	-8.452953	2.598125
C	-7.370217	8.461145	-0.986732
H	-6.562514	8.434002	-0.241588
C	-8.649902	7.851165	-0.386409
H	-8.964364	8.451963	0.483703
H	-8.402960	6.840391	-0.002043
O	-9.718998	7.794607	-1.307128
H	-9.312358	7.799583	-2.202337
C	-6.991083	7.744090	-2.272105
O	-7.809524	7.677857	-3.192448
N	-5.760316	7.167343	-2.346250
C	-5.317354	6.458956	-3.545379
C	-4.191154	7.178391	-4.319391
C	-2.898852	7.252077	-3.569722
C	-1.835564	6.381205	-3.507839
N	-2.612387	8.217133	-2.616137
C	-1.434122	7.898441	-2.023477
N	-0.946238	6.784712	-2.535689
H	-5.171762	7.186917	-1.513860
H	-6.198642	6.367976	-4.193783
H	-4.534576	8.184666	-4.613732
H	-4.019394	6.617807	-5.250838
H	-1.668075	5.480120	-4.089648

H	-0.974524	8.469826	-1.222342
H	-3.177531	9.033300	-2.412254
Fe	0.467025	5.372134	-1.402807
O	1.405161	4.068832	-0.465706
O	2.069192	5.707144	-2.778044
C	1.549211	4.897814	-3.606837
O	0.442196	4.354726	-3.320240
C	2.244718	4.565981	-4.901599
H	3.079389	5.253231	-5.089851
H	2.624820	3.532950	-4.847311
C	-2.437237	-3.174037	-5.094696
C	-3.577528	-3.198515	-4.079390
C	-1.210468	-2.476715	-4.484398
O	-4.023152	-2.156543	-3.589028
C	-0.012295	-2.415482	-5.433602
S	1.304537	-1.289098	-4.825336
N	-4.009372	-4.420589	-3.720128
C	-5.021971	-4.660845	-2.715759
C	-5.416780	-6.140569	-2.785323
O	-5.300822	-6.763236	-3.829694
N	-5.914566	-6.648555	-1.625204
C	-6.231578	-8.061229	-1.491631
C	-7.574237	-8.379588	-0.826418
O	-7.795744	-9.505289	-0.413413
N	-8.468448	-7.353166	-0.773765
C	-9.807681	-7.495405	-0.228530
C	-10.066325	-6.703544	1.069356
C	-9.164762	-7.167440	2.218995
C	-9.968326	-5.187851	0.851100
C	2.030587	-2.289051	-3.557234
O	1.777336	-3.459073	-3.370467
C	2.987802	-1.530581	-2.627696
N	4.129389	-2.421083	-2.321470
C	2.255621	-1.144769	-1.312114
N	1.034654	-0.419182	-1.549171
C	0.631282	0.827856	-1.125843
O	-0.531550	1.183587	-1.331786
C	1.577820	1.707948	-0.432929
C	1.050190	2.930353	0.253366
C	1.453842	3.076099	1.736248
O	1.579204	4.206231	2.234992
N	1.580593	1.954188	2.447531
H	-2.783925	-2.614396	-5.980329
H	-2.182623	-4.193063	-5.426286

H	-0.912549	-3.013436	-3.569261
H	-1.503053	-1.459564	-4.183911
H	0.443005	-3.403854	-5.592464
H	-0.288116	-1.997781	-6.413676
H	-3.734419	-5.243852	-4.251967
H	-4.657974	-4.401242	-1.708493
H	-5.648605	-6.155187	-0.770137
H	-6.238683	-8.498922	-2.501102
H	-8.176522	-6.462831	-1.158977
H	-9.948573	-8.570723	-0.047837
H	-11.113672	-6.936653	1.339819
H	3.379029	-0.613840	-3.089877
H	2.048865	-2.069963	-0.743759
H	2.945483	-0.541733	-0.708910
H	0.260822	-0.931195	-1.963433
H	2.657557	1.581429	-0.543190
H	-0.049562	2.815203	0.287219
H	1.706376	2.032079	3.462987
H	1.449535	1.003402	2.084639
H	4.849745	-2.422749	-3.089082
H	4.639212	-2.152380	-1.431622
H	3.867354	-3.421233	-2.223374
H	-8.932887	-4.878789	0.634244
H	-10.619813	-4.855103	0.024794
H	-10.265770	-4.639274	1.758403
H	-8.109652	-6.926873	2.014482
H	-9.243719	-8.254364	2.378424
H	-9.431699	-6.656809	3.157615
H	10.526595	2.743315	-3.888375
H	9.502196	3.149172	5.854772
H	2.986748	-8.094956	-1.208001
H	7.228446	-4.176273	-6.838768
H	-4.983664	-7.720390	2.991709
H	-3.668950	-3.500716	7.876549
H	-5.917563	-4.043984	-2.909577
H	-10.543270	-7.187485	-0.994052
H	-5.448974	-8.585369	-0.921482
H	0.524086	7.339900	4.595135
H	1.525856	4.605015	-5.732797
H	-4.991482	5.439277	-3.284946
H	-7.565675	9.512610	-1.257053
H	-3.856390	8.267420	3.868300
H	-3.130494	0.050467	-4.893135
H	-2.819164	0.743150	7.402472

O	-0.414997	-2.666842	3.067420
H	-0.162761	-3.601747	3.044034
H	0.338341	-2.170609	2.677405
O	1.616240	-0.914436	2.442718
H	1.708061	-0.771637	3.420985
H	2.549886	-0.986762	2.146966
O	4.341655	-0.662799	1.964552
H	4.650935	-0.879015	2.867478
H	4.850243	-1.210433	1.305835
O	5.579440	-1.859950	-0.047174
H	6.146428	-2.707809	-0.019445
H	6.230553	-1.211622	-0.407933
O	7.458373	-0.641155	-1.536242
H	8.341095	-0.235782	-1.407947
H	7.673812	-1.588017	-1.777172
O	5.134597	0.812320	-2.086689
H	5.214777	1.415817	-1.333737
H	5.969998	0.297273	-2.061042
O	5.004905	1.871930	2.684321
H	5.968021	1.985964	2.526720
H	4.740975	1.052512	2.196197
O	7.512220	2.679733	2.030924
H	7.830312	3.506955	2.418909
H	8.339544	2.137971	1.871852
O	2.040366	-0.471063	5.121822
H	3.015359	-0.384061	5.172325
H	1.685067	0.418377	5.348442
O	4.802976	-0.067172	4.643048
H	5.613154	-0.020188	5.184882
H	4.797706	0.789616	4.158001
O	-0.288188	-2.061420	5.837059
H	0.608633	-1.682456	5.801406
H	-0.482550	-2.221191	4.887573

^{5}**TS2**$_{A}$

Element	X	Y	Z
C	9.330854	1.902668	5.839353
H	9.751253	1.433459	6.748505
C	7.813384	1.724981	5.811346
H	7.399190	2.110761	4.868037
H	7.351260	2.292562	6.637433
O	7.406191	0.349698	5.869624
H	7.654211	-0.018253	6.730814

C	10.118445	1.374120	4.635904
O	11.178556	1.866377	4.291056
N	9.562201	0.307978	3.989126
H	8.721024	-0.105630	4.384101
C	10.240372	-0.358930	2.902430
H	11.249526	-0.659577	3.241078
C	9.457006	-1.663318	2.555557
H	9.398316	-2.214989	3.518258
C	8.044033	-1.420923	2.039952
H	8.071560	-0.848166	1.101742
H	7.416816	-0.884978	2.768428
H	7.578647	-2.391283	1.819707
O	10.114028	-2.441104	1.587868
H	11.073648	-2.519985	1.733762
C	10.457487	0.587544	1.702769
O	9.694853	1.517083	1.416679
N	11.548383	0.320473	0.968842
H	12.106573	-0.504568	1.188750
C	11.936449	1.048735	-0.225243
H	12.296771	2.050613	0.070622
C	13.105083	0.311499	-0.929655
H	13.540103	0.996237	-1.673511
H	13.886557	0.132256	-0.174315
C	12.764201	-1.018165	-1.629070
H	13.706460	-1.447175	-2.010226
H	12.111616	-0.856469	-2.498707
C	12.153347	-2.049982	-0.686454
O	12.632457	-2.227611	0.442041
N	11.090661	-2.738608	-1.139462
H	10.558905	-3.245838	-0.435930
H	10.545594	-2.401445	-1.926476
C	10.764249	1.222037	-1.206682
O	9.979832	0.302506	-1.425909
N	10.749700	2.394375	-1.881366
H	11.398178	3.118951	-1.593033
C	9.838622	2.685531	-2.982389
H	9.405706	1.723905	-3.292678
C	8.721012	3.675412	-2.636111
H	9.161090	4.645632	-2.341012
H	8.156608	3.868951	-3.565303
C	7.772980	3.174549	-1.544961
H	7.403303	2.174098	-1.813452
H	8.321388	3.063092	-0.594627
C	6.575912	4.098261	-1.340945

H	6.911612	5.138774	-1.171419
H	5.964050	4.106681	-2.258342
N	5.753219	3.645878	-0.211608
H	6.280256	3.152054	0.517042
C	4.771741	4.461556	0.279762
N	4.283039	5.448651	-0.459361
H	3.533059	6.018816	-0.007905
H	4.227198	5.362888	-1.471861
N	4.351708	4.302239	1.537055
H	3.472286	4.755867	1.799651
H	4.597621	3.455045	2.068511
C	7.176843	-3.849500	-5.878887
H	8.213226	-3.654726	-5.556637
C	6.333827	-2.575339	-5.668005
H	6.892411	-1.739950	-6.132823
C	4.955185	-2.653599	-6.309552
H	4.383539	-1.735982	-6.101316
H	4.397288	-3.525473	-5.936793
H	5.045721	-2.753883	-7.401633
O	6.155817	-2.276965	-4.278642
H	6.926663	-2.521444	-3.724181
C	6.621476	-5.072698	-5.152318
O	5.711116	-5.757466	-5.592953
N	7.223263	-5.323614	-3.953859
H	7.796174	-4.579205	-3.565985
C	6.695926	-6.306655	-3.034207
H	6.697977	-7.282853	-3.546607
C	7.575823	-6.413705	-1.748002
H	6.916503	-6.616023	-0.890668
H	8.235333	-7.290041	-1.845931
C	8.469372	-5.202549	-1.412588
H	9.279752	-5.107775	-2.151001
H	8.937975	-5.409385	-0.437045
C	7.730263	-3.861126	-1.285263
O	7.944317	-2.991581	-2.203339
O	6.981552	-3.719151	-0.299526
C	5.231482	-6.009375	-2.639255
O	4.837805	-4.863211	-2.437836
N	4.478592	-7.110400	-2.433335
H	4.853519	-7.998962	-2.745701
C	3.115905	-7.095914	-1.920037
H	3.003948	-6.191664	-1.305551
C	2.022864	-7.128648	-3.006424
H	2.094472	-6.204906	-3.602165

H	2.223544	-7.971248	-3.689503
C	0.646677	-7.274769	-2.395357
C	0.039038	-8.535036	-2.264439
H	0.537846	-9.418471	-2.675698
C	-1.189696	-8.691489	-1.625951
H	-1.658038	-9.673635	-1.532576
C	-1.852871	-7.577452	-1.084386
O	-3.031302	-7.777429	-0.460016
H	-3.404253	-6.938256	-0.094572
C	-1.264478	-6.306704	-1.218668
H	-1.777690	-5.434684	-0.806346
C	-0.033723	-6.167231	-1.864676
H	0.410803	-5.172171	-1.959904
C	-3.178171	1.012494	-4.784907
H	-2.427523	1.190585	-3.991526
C	-2.736939	1.703191	-6.093977
H	-3.627900	1.786866	-6.743689
C	-2.211134	3.120190	-5.831699
H	-2.971378	3.698522	-5.289531
H	-1.288429	3.089210	-5.225187
H	-1.982047	3.651934	-6.768409
C	-1.709900	0.825284	-6.836506
H	-2.144274	-0.183558	-6.960233
H	-0.818796	0.698504	-6.193091
C	-1.279924	1.344318	-8.210256
H	-0.736944	2.299744	-8.142304
H	-2.151022	1.503495	-8.867815
H	-0.613136	0.626316	-8.714303
C	-4.533893	1.469549	-4.255406
O	-4.926801	2.635054	-4.317377
N	-5.293153	0.487360	-3.703528
H	-4.923094	-0.464026	-3.641870
C	-6.629149	0.758612	-3.217353
H	-7.245474	1.133732	-4.054617
C	-7.268849	-0.517974	-2.658799
H	-6.704867	-0.890922	-1.792046
H	-7.278610	-1.301911	-3.430826
H	-8.305662	-0.325880	-2.348036
C	-6.620097	1.878737	-2.161697
O	-5.648506	2.105884	-1.436800
N	-7.770071	2.569323	-2.043446
H	-8.554420	2.427185	-2.690808
C	-7.866547	3.802863	-1.281576
H	-7.384940	4.615017	-1.858918

C	-9.343925	4.174004	-1.055856
H	-9.809764	3.373305	-0.459731
H	-9.399513	5.102319	-0.471055
C	-10.119062	4.295158	-2.367185
O	-10.168343	3.349528	-3.151958
N	-10.738897	5.473201	-2.581711
H	-10.524974	6.307019	-2.029731
H	-11.230263	5.585946	-3.462910
C	-7.165018	3.707720	0.086860
O	-7.520842	2.915989	0.950017
N	-6.209143	4.650916	0.288885
C	-5.414116	4.723302	1.490984
C	-4.877080	6.149987	1.643304
C	-4.226075	3.706164	1.507211
O	-4.770453	6.911250	0.685980
C	-3.201906	3.911794	0.436063
C	-1.994652	4.579070	0.435895
N	-3.344646	3.419224	-0.847430
C	-2.259075	3.771217	-1.556096
N	-1.421362	4.483139	-0.814243
H	-5.922205	5.248102	-0.479753
H	-6.069427	4.479060	2.342491
H	-3.724141	3.768264	2.484600
H	-4.663603	2.699139	1.429878
H	-1.500007	5.091923	1.257138
H	-2.090509	3.471351	-2.585694
H	-4.146216	2.857472	-1.186613
N	-4.488653	6.467008	2.905873
H	-4.478031	5.716592	3.589749
C	-3.591160	7.557653	3.200355
H	-3.529478	8.197911	2.309092
C	-2.203621	7.006074	3.584192
O	-2.072439	5.821404	3.888077
N	-1.197855	7.898997	3.551110
C	0.217941	7.569460	3.732127
C	1.103542	8.530681	2.937009
C	0.861384	8.490941	1.424837
C	1.442165	7.248928	0.731769
O	2.589681	6.899409	1.043845
O	0.702465	6.720323	-0.164240
H	-1.423552	8.859059	3.311680
H	0.369445	6.534255	3.390975
H	0.956672	9.558495	3.316316
H	2.155961	8.271857	3.127811

H	1.363172	9.356916	0.958947
H	-0.209498	8.572248	1.183270
C	-3.003071	0.658734	6.374410
H	-3.821328	1.314601	6.030022
C	-1.668179	1.081718	5.749967
H	-1.796866	1.161088	4.658264
H	-0.932583	0.280111	5.913577
C	-1.143153	2.407767	6.321520
H	-0.897052	2.279210	7.389467
H	-1.909633	3.197603	6.254739
C	0.122992	2.841045	5.605840
O	1.141828	2.127990	5.613775
N	0.081276	4.001735	4.936806
H	-0.777945	4.533578	4.806475
H	0.846064	4.217061	4.287269
C	-3.362812	-0.768687	5.987606
O	-3.378801	-1.154679	4.826302
N	-3.679894	-1.591604	7.032683
H	-3.539949	-1.231832	7.968118
C	-3.697214	-3.031570	6.874102
H	-2.747832	-3.373455	6.430949
C	-4.817588	-3.608086	6.012679
O	-4.741150	-4.779566	5.637753
N	-5.845655	-2.795942	5.690623
H	-5.813905	-1.800495	5.919854
C	-6.892824	-3.236312	4.783896
H	-7.271117	-4.210017	5.133597
C	-8.065325	-2.235234	4.756263
H	-8.841014	-2.662149	4.106046
H	-8.484932	-2.167664	5.774132
C	-7.681761	-0.871917	4.263505
N	-6.796567	-0.073958	4.959069
C	-6.656261	1.017841	4.232807
H	-6.024753	1.870222	4.482862
N	-7.404686	0.973827	3.099772
H	-7.457588	1.689493	2.368193
C	-8.065096	-0.231382	3.101881
H	-8.734531	-0.529824	2.299414
C	-6.401682	-3.532274	3.353074
O	-7.129910	-4.141464	2.579786
N	-5.145616	-3.134353	3.018897
H	-4.587576	-2.560915	3.653694
C	-4.584316	-3.544502	1.747470
H	-5.342943	-3.385695	0.966557

C	-3.306906	-2.764912	1.397557
H	-2.543989	-2.929506	2.175637
H	-2.910492	-3.204880	0.466773
C	-3.490876	-1.250214	1.185622
H	-3.803021	-0.809793	2.148904
C	-2.140535	-0.625031	0.813544
H	-1.786140	-1.008044	-0.159374
H	-2.218742	0.469231	0.712765
H	-1.375208	-0.857026	1.569250
C	-4.555315	-0.931291	0.130117
H	-4.607686	0.147388	-0.082200
H	-4.329870	-1.436880	-0.825342
H	-5.561565	-1.245078	0.451835
C	-4.309185	-5.055836	1.673518
O	-4.198765	-5.570243	0.539233
N	-4.171503	-5.734345	2.813003
H	-4.361189	-5.251460	3.696478
C	-3.961529	-7.177174	2.887368
H	-3.449093	-7.503821	1.973139
C	-3.156559	-7.580764	4.131991
H	-3.143963	-8.682841	4.161016
H	-3.696284	-7.243818	5.030889
C	-1.737755	-7.051049	4.160728
C	-1.399934	-5.953797	4.966911
H	-2.178314	-5.494056	5.580561
C	-0.095534	-5.445334	4.990631
H	0.128999	-4.574233	5.612433
C	0.894431	-6.035659	4.196789
H	1.917747	-5.649895	4.215868
C	0.571315	-7.130137	3.385216
H	1.339991	-7.599468	2.765433
C	-0.732481	-7.631277	3.368246
H	-0.972395	-8.488119	2.731440
C	-7.070821	8.505519	-1.179145
H	-6.287439	8.455627	-0.409822
C	-8.393139	7.967415	-0.602868
H	-8.702277	8.600269	0.246146
H	-8.204050	6.954788	-0.191487
O	-9.439190	7.940865	-1.551376
H	-9.009189	7.908021	-2.434960
C	-6.692045	7.744362	-2.438716
O	-7.487439	7.695543	-3.379828
N	-5.487748	7.109237	-2.469273
C	-5.053571	6.346655	-3.639393

C	-3.917030	7.014150	-4.446152
C	-2.620549	7.095098	-3.704790
C	-1.546831	6.235997	-3.643331
N	-2.349382	8.056396	-2.741956
C	-1.173108	7.744002	-2.142740
N	-0.670295	6.639378	-2.659167
H	-4.918785	7.122573	-1.624423
H	-5.935979	6.242171	-4.284135
H	-4.245837	8.013025	-4.780316
H	-3.759431	6.413519	-5.354723
H	-1.364124	5.339002	-4.228372
H	-0.731304	8.307310	-1.325927
H	-2.921946	8.867792	-2.539920
Fe	0.537133	5.149987	-1.450238
O	1.356523	3.726644	-0.304895
O	2.366839	5.170721	-2.581970
C	1.893938	4.313890	-3.399719
O	0.689963	3.955598	-3.292251
C	2.802405	3.695036	-4.433819
H	3.451639	4.461468	-4.881586
H	3.449865	2.955573	-3.930920
C	-2.533311	-3.341359	-5.104480
C	-3.667287	-3.330249	-4.081674
C	-1.310080	-2.601601	-4.540326
O	-4.093452	-2.273122	-3.606723
C	-0.127912	-2.573845	-5.509840
S	1.171163	-1.371363	-5.021759
N	-4.118902	-4.539038	-3.703900
C	-5.126565	-4.748899	-2.687727
C	-5.515397	-6.231510	-2.708786
O	-5.418486	-6.881948	-3.737896
N	-5.985022	-6.710704	-1.524584
C	-6.284188	-8.121977	-1.345252
C	-7.607097	-8.435458	-0.639450
O	-7.805065	-9.550715	-0.187696
N	-8.513612	-7.419378	-0.599286
C	-9.844523	-7.567403	-0.035377
C	-10.103806	-6.749616	1.245970
C	-9.182125	-7.168991	2.396920
C	-10.036803	-5.237666	0.992122
C	1.964741	-2.244050	-3.697498
O	1.735914	-3.392645	-3.393391
C	2.972982	-1.396602	-2.900748
N	4.081488	-2.289262	-2.493868

C	2.320655	-0.767011	-1.647523
N	1.213610	0.093048	-2.003501
C	0.673228	1.140252	-1.290355
O	-0.485027	1.489046	-1.493013
C	1.510446	1.818361	-0.278220
C	0.887033	2.799668	0.621501
C	1.392083	3.009898	2.043866
O	1.546980	4.161178	2.474952
N	1.570321	1.909573	2.774605
H	-2.892524	-2.828932	-6.013757
H	-2.270526	-4.372484	-5.388827
H	-0.993797	-3.084844	-3.602331
H	-1.614077	-1.575036	-4.288507
H	0.353973	-3.557582	-5.608354
H	-0.429759	-2.234359	-6.512460
H	-3.854119	-5.375585	-4.219860
H	-4.755902	-4.456148	-1.692394
H	-5.698464	-6.193565	-0.691075
H	-6.312390	-8.586198	-2.342502
H	-8.241057	-6.536917	-1.015728
H	-9.967598	-8.640005	0.172451
H	-11.143727	-6.996399	1.532452
H	3.385963	-0.581000	-3.510282
H	2.009849	-1.580272	-0.962964
H	3.108737	-0.184339	-1.154548
H	0.514352	-0.302514	-2.628411
H	2.583955	1.649209	-0.203943
H	-0.212067	2.711832	0.641396
H	1.742685	2.016424	3.780663
H	1.419323	0.948082	2.440524
H	4.830477	-2.334389	-3.234459
H	4.555101	-1.990918	-1.594139
H	3.793310	-3.278817	-2.366422
H	-9.008250	-4.912302	0.767160
H	-10.696671	-4.937411	0.160050
H	-10.347855	-4.675081	1.886680
H	-8.135783	-6.901027	2.181818
H	-9.229003	-8.254841	2.575056
H	-9.460159	-6.652420	3.329451
H	10.428843	3.074294	-3.828631
H	9.576341	2.972573	5.921457
H	2.995586	-7.963192	-1.250824
H	7.203476	-4.079646	-6.954230
H	-4.940725	-7.688764	2.909799

H	-3.776987	-3.496486	7.868293
H	-6.026139	-4.142658	-2.896842
H	-10.592502	-7.288826	-0.800134
H	-5.481928	-8.624677	-0.783084
H	0.482941	7.602815	4.803131
H	2.221849	3.183889	-5.212172
H	-4.746024	5.334504	-3.334013
H	-7.210358	9.558777	-1.475832
H	-3.978261	8.172758	4.032125
H	-3.217821	-0.078194	-4.921012
H	-2.958326	0.758608	7.471753
O	-0.416910	-2.711668	3.255139
H	-0.152129	-3.643156	3.230946
H	0.332076	-2.202844	2.873120
O	1.582076	-0.924076	2.660389
H	1.710879	-0.796584	3.637037
H	2.499017	-0.988229	2.313751
O	4.269710	-0.658573	2.005191
H	4.634475	-0.939773	2.867595
H	4.746238	-1.136561	1.272782
O	5.437759	-1.667335	-0.169342
H	6.010033	-2.513461	-0.156313
H	6.088271	-1.008134	-0.510774
O	7.332538	-0.429168	-1.636944
H	8.218489	-0.034150	-1.496724
H	7.544676	-1.372766	-1.888595
O	5.014855	1.062317	-2.209316
H	5.097041	1.684159	-1.469888
H	5.852587	0.550947	-2.181900
O	4.997451	1.824297	2.833268
H	5.954420	1.943109	2.639996
H	4.707255	1.032095	2.316645
O	7.459698	2.651222	2.098486
H	7.761558	3.487057	2.481015
H	8.298426	2.135860	1.913959
O	2.056362	-0.467824	5.320392
H	3.034519	-0.398875	5.339878
H	1.715458	0.414738	5.587183
O	4.809264	-0.175492	4.716973
H	5.632278	-0.155514	5.240603
H	4.814673	0.692604	4.251753
O	-0.317975	-2.062949	6.005041
H	0.583390	-1.695711	5.983919
H	-0.495497	-2.237912	5.054582

$^{5}\mathbf{P}_{A}$

Element	X	Y	Z
C	9.517202	1.514847	5.844989
H	10.035658	0.918054	6.618827
C	8.004594	1.328327	5.966545
H	7.492129	1.849396	5.145129
H	7.649218	1.764512	6.916428
O	7.589409	-0.041102	5.867222
H	7.922249	-0.528393	6.635355
C	10.160033	1.180285	4.494389
O	11.146602	1.767894	4.085704
N	9.572886	0.163200	3.798846
H	8.780801	-0.311160	4.226268
C	10.146974	-0.365792	2.583159
H	11.205648	-0.624101	2.772635
C	9.397000	-1.688258	2.223572
H	9.465920	-2.302359	3.147060
C	7.927304	-1.486844	1.874128
H	7.830932	-0.871358	0.967849
H	7.367449	-1.005794	2.689861
H	7.474687	-2.464707	1.658029
O	9.987318	-2.364401	1.143071
H	10.957613	-2.397303	1.197695
C	10.156041	0.671805	1.442907
O	9.341312	1.597689	1.352876
N	11.109739	0.491164	0.517676
H	11.718833	-0.327651	0.567245
C	11.216552	1.291167	-0.689359
H	11.527122	2.314731	-0.413389
C	12.300769	0.695241	-1.622973
H	12.531287	1.444734	-2.395440
H	13.218071	0.565810	-1.027246
C	11.961356	-0.633074	-2.322567
H	12.839012	-0.930108	-2.922575
H	11.124508	-0.514141	-3.025619
C	11.681501	-1.780976	-1.357984
O	12.329590	-1.897553	-0.308851
N	10.726847	-2.657685	-1.714308
H	10.427599	-3.303844	-0.990011
H	10.010160	-2.428542	-2.396791
C	9.869187	1.368326	-1.431442
O	9.153546	0.373824	-1.515015
N	9.608506	2.541198	-2.049040
H	10.237771	3.317357	-1.873432

C	8.449513	2.789845	-2.901987
H	7.931041	1.829745	-3.029634
C	7.496855	3.850101	-2.338856
H	8.060316	4.788025	-2.175102
H	6.749254	4.078888	-3.117145
C	6.792496	3.430731	-1.046031
H	6.127101	2.575332	-1.254713
H	7.544564	3.086802	-0.314891
C	5.984047	4.572914	-0.427673
H	6.615210	5.480117	-0.348641
H	5.133206	4.833507	-1.075068
N	5.486182	4.191731	0.889801
H	6.099046	3.586489	1.442799
C	4.564740	4.884244	1.580873
N	3.945075	5.942930	1.064579
H	3.112081	6.245652	1.590076
H	3.860319	6.031733	0.046883
N	4.314295	4.521584	2.856055
H	3.511850	4.962109	3.295866
H	4.506572	3.543905	3.113510
C	7.026923	-4.579983	-5.926416
H	8.045929	-4.282735	-5.627539
C	6.100467	-3.347918	-5.862854
H	6.611970	-2.532739	-6.410150
C	4.741913	-3.585366	-6.506259
H	4.117135	-2.683320	-6.421566
H	4.224140	-4.433625	-6.034268
H	4.860657	-3.814662	-7.575903
O	5.880146	-2.915398	-4.515534
H	6.669711	-3.003775	-3.941224
C	6.550945	-5.747286	-5.064808
O	5.711656	-6.555894	-5.429419
N	7.142213	-5.799671	-3.835751
H	7.637696	-4.966531	-3.532403
C	6.689814	-6.715617	-2.812778
H	6.796450	-7.740286	-3.205423
C	7.548826	-6.581284	-1.512846
H	6.889593	-6.700302	-0.639987
H	8.264466	-7.417343	-1.479001
C	8.353843	-5.276929	-1.345688
H	9.143057	-5.214954	-2.110453
H	8.850688	-5.327612	-0.363290
C	7.512705	-3.988650	-1.352678
O	7.653093	-3.202242	-2.356040

O	6.758441	-3.811458	-0.377720
C	5.198668	-6.512846	-2.469646
O	4.715173	-5.388202	-2.355594
N	4.518270	-7.644115	-2.193752
H	4.948583	-8.528102	-2.440212
C	3.152819	-7.659298	-1.686924
H	3.021609	-6.758195	-1.070658
C	2.054616	-7.704045	-2.768135
H	2.154151	-6.815327	-3.410820
H	2.215063	-8.587332	-3.409049
C	0.682432	-7.755392	-2.133423
C	0.072558	-8.979798	-1.810722
H	0.561552	-9.916703	-2.096205
C	-1.145888	-9.034037	-1.137107
H	-1.615985	-9.989358	-0.894062
C	-1.796232	-7.849174	-0.752301
O	-2.963183	-7.951666	-0.083864
H	-3.323825	-7.069866	0.179436
C	-1.208345	-6.615112	-1.082062
H	-1.713226	-5.689366	-0.795178
C	0.011343	-6.579994	-1.762466
H	0.453548	-5.612250	-2.018942
C	-2.809104	1.037283	-5.066815
H	-2.163627	0.842900	-4.190694
C	-2.132166	2.038265	-6.018668
H	-2.908474	2.413032	-6.709691
C	-1.573320	3.243934	-5.250019
H	-2.370229	3.698164	-4.644614
H	-0.749411	2.942924	-4.579989
H	-1.186470	4.018227	-5.931225
C	-1.054676	1.326834	-6.860639
H	-1.522122	0.454129	-7.353219
H	-0.282856	0.915904	-6.183120
C	-0.386783	2.191917	-7.931476
H	0.192882	3.019789	-7.494002
H	-1.134333	2.633084	-8.612165
H	0.308771	1.596735	-8.544684
C	-4.181589	1.469135	-4.556191
O	-4.630000	2.605115	-4.692230
N	-4.897268	0.491756	-3.935393
H	-4.520276	-0.454875	-3.854960
C	-6.250584	0.750408	-3.498511
H	-6.838776	1.114630	-4.360572
C	-6.905110	-0.524973	-2.955249

H	-6.374049	-0.892004	-2.066389
H	-6.880615	-1.313747	-3.721484
H	-7.953418	-0.334068	-2.685046
C	-6.297046	1.886326	-2.458321
O	-5.358078	2.164701	-1.709305
N	-7.477012	2.535778	-2.391061
H	-8.220170	2.365192	-3.078431
C	-7.651782	3.766046	-1.642785
H	-7.137047	4.588838	-2.174663
C	-9.150230	4.109453	-1.533445
H	-9.641221	3.304941	-0.963084
H	-9.268022	5.042460	-0.965944
C	-9.836624	4.198060	-2.896240
O	-9.813234	3.242666	-3.669786
N	-10.469990	5.357567	-3.165757
H	-10.324494	6.200230	-2.605186
H	-10.907498	5.447141	-4.077382
C	-7.070630	3.673486	-0.220182
O	-7.475696	2.854176	0.595833
N	-6.170532	4.642158	0.086136
C	-5.525285	4.726054	1.374927
C	-4.995714	6.152742	1.567047
C	-4.384557	3.675425	1.562155
O	-4.805561	6.912998	0.621955
C	-3.195115	3.844944	0.672144
C	-2.011060	4.522106	0.873169
N	-3.090464	3.299955	-0.593021
C	-1.889241	3.651514	-1.099827
N	-1.210787	4.398416	-0.238086
H	-5.820120	5.253436	-0.644387
H	-6.286942	4.517557	2.144730
H	-4.041730	3.717998	2.607715
H	-4.837817	2.683617	1.416638
H	-1.700070	5.080501	1.751207
H	-1.530361	3.316348	-2.069444
H	-3.821985	2.754694	-1.076836
N	-4.735988	6.490837	2.856519
H	-4.806241	5.762890	3.559194
C	-3.975194	7.657103	3.227123
H	-3.798507	8.242693	2.312281
C	-2.629953	7.261726	3.861972
O	-2.322086	6.076638	3.973720
N	-1.857913	8.293259	4.260990
C	-0.493765	8.160752	4.772958

C	0.525753	8.984744	3.977056
C	0.516088	8.731519	2.469840
C	0.968372	7.336643	2.014491
O	1.741697	6.680184	2.745661
O	0.541975	6.984186	0.870274
H	-2.221394	9.233355	4.137446
H	-0.236923	7.095769	4.732949
H	0.355849	10.060355	4.163793
H	1.523243	8.748924	4.379178
H	1.204248	9.442791	1.979486
H	-0.475827	8.930081	2.034253
C	-2.626170	1.054504	5.876554
H	-3.477722	1.679961	5.555588
C	-1.354663	1.448836	5.113515
H	-1.567973	1.446232	4.032559
H	-0.591311	0.677365	5.284352
C	-0.836946	2.826666	5.556998
H	-0.482592	2.765509	6.600868
H	-1.650672	3.569593	5.536419
C	0.329698	3.323924	4.720158
O	1.398630	2.689741	4.643705
N	0.133158	4.473351	4.061306
H	-0.755103	4.973480	4.115454
H	0.859263	4.890420	3.473820
C	-3.018378	-0.399066	5.642057
O	-3.104655	-0.888764	4.523897
N	-3.306594	-1.109725	6.774830
H	-3.089727	-0.669444	7.659812
C	-3.339389	-2.558947	6.762938
H	-2.413390	-2.951513	6.313642
C	-4.503506	-3.212106	6.023395
O	-4.446983	-4.414968	5.761449
N	-5.548834	-2.430967	5.679196
H	-5.508936	-1.418531	5.814501
C	-6.645660	-2.954529	4.881483
H	-6.992012	-3.894695	5.339594
C	-7.827097	-1.965045	4.837313
H	-8.634580	-2.448788	4.271144
H	-8.188313	-1.812482	5.868230
C	-7.483319	-0.647310	4.209830
N	-6.566388	0.209224	4.784382
C	-6.475149	1.235195	3.961349
H	-5.833714	2.105010	4.100808
N	-7.285400	1.093575	2.879613

H	-7.380406	1.745146	2.092863
C	-7.935413	-0.108893	3.021286
H	-8.645365	-0.477284	2.285430
C	-6.240130	-3.375084	3.454108
O	-7.016824	-4.030152	2.772142
N	-4.997156	-3.027822	3.027720
H	-4.407444	-2.408436	3.585480
C	-4.492634	-3.534369	1.768178
H	-5.279829	-3.429530	1.006099
C	-3.228485	-2.781023	1.327012
H	-2.447432	-2.893596	2.096681
H	-2.852255	-3.281790	0.419124
C	-3.424138	-1.284877	1.022262
H	-3.777255	-0.791853	1.944059
C	-2.065477	-0.669321	0.672353
H	-1.647900	-1.152383	-0.227859
H	-2.155777	0.407206	0.456789
H	-1.352595	-0.814290	1.497645
C	-4.449303	-1.043561	-0.090423
H	-4.517727	0.023412	-0.353312
H	-4.173709	-1.586159	-1.011648
H	-5.459912	-1.368825	0.205155
C	-4.207917	-5.046002	1.792140
O	-4.109620	-5.637226	0.694748
N	-4.042751	-5.635489	2.976669
H	-4.205337	-5.081004	3.822524
C	-3.812090	-7.064405	3.165976
H	-3.340945	-7.467155	2.260450
C	-2.944818	-7.350175	4.401368
H	-2.909813	-8.445851	4.520597
H	-3.449943	-6.948615	5.293817
C	-1.536039	-6.798269	4.324061
C	-1.173537	-5.659388	5.058653
H	-1.926526	-5.180271	5.688914
C	0.122637	-5.134138	4.989434
H	0.367329	-4.232902	5.558517
C	1.078674	-5.748462	4.172414
H	2.096367	-5.350739	4.120632
C	0.729520	-6.883561	3.429919
H	1.471275	-7.371610	2.792092
C	-0.565281	-7.402024	3.506197
H	-0.824653	-8.290853	2.923242
C	-7.058201	8.488040	-1.369964
H	-6.373484	8.468802	-0.510145

C	-8.426834	7.919884	-0.954909
H	-8.856137	8.550589	-0.158103
H	-8.263423	6.915745	-0.512387
O	-9.344717	7.857706	-2.025656
H	-8.806617	7.821326	-2.848241
C	-6.503774	7.725367	-2.562775
O	-7.176783	7.638021	-3.592529
N	-5.283738	7.137633	-2.433658
C	-4.664546	6.393809	-3.529004
C	-3.437688	7.099398	-4.146730
C	-2.289934	7.244562	-3.198442
C	-1.208571	6.431963	-2.942838
N	-2.216510	8.234432	-2.229986
C	-1.137132	7.983882	-1.443267
N	-0.514937	6.894020	-1.845720
H	-4.828256	7.175866	-1.522345
H	-5.436896	6.265427	-4.298586
H	-3.745962	8.080495	-4.546728
H	-3.105505	6.497388	-5.005953
H	-0.895139	5.533717	-3.468741
H	-0.850100	8.587334	-0.585987
H	-2.847659	9.022032	-2.141678
Fe	0.654212	5.498383	-0.548697
O	2.459064	0.810498	-0.557995
O	2.808872	5.796318	-1.519456
C	2.416578	4.986401	-2.401885
O	1.251033	4.477633	-2.335467
C	3.325589	4.550325	-3.530126
H	4.179479	5.232551	-3.637532
H	3.694019	3.528694	-3.328531
C	-2.656496	-3.532725	-5.465900
C	-3.664784	-3.473104	-4.317892
C	-1.346992	-2.878255	-4.999378
O	-3.970572	-2.400830	-3.789430
C	-0.214676	-2.880014	-6.028655
S	1.178645	-1.820679	-5.460192
N	-4.122537	-4.661595	-3.883529
C	-5.002290	-4.829123	-2.748265
C	-5.410486	-6.306510	-2.688386
O	-5.389952	-6.990772	-3.700791
N	-5.807796	-6.740430	-1.462901
C	-6.138647	-8.135808	-1.220103
C	-7.475677	-8.395570	-0.517750
O	-7.704165	-9.493184	-0.038367

N	-8.356612	-7.356790	-0.510523
C	-9.691965	-7.454562	0.054159
C	-9.928080	-6.598223	1.314665
C	-9.019303	-7.016342	2.476139
C	-9.816344	-5.096038	1.022226
C	1.886162	-2.869459	-4.204958
O	1.695265	-4.060120	-4.114347
C	2.666516	-2.104987	-3.128740
N	3.829075	-2.909495	-2.700746
C	1.718562	-1.880823	-1.916961
N	0.607822	-1.017845	-2.250352
C	0.295197	0.208664	-1.749733
O	-0.596075	0.896855	-2.221330
C	1.037991	0.652995	-0.504901
C	1.636890	1.991736	-0.468066
C	1.725242	2.782263	0.814237
O	1.577406	4.017969	0.780728
N	1.971509	2.098581	1.925779
H	-3.073676	-2.975964	-6.322130
H	-2.487610	-4.570803	-5.792603
H	-0.999047	-3.403139	-4.094133
H	-1.580938	-1.843078	-4.707131
H	0.182232	-3.887681	-6.218856
H	-0.522484	-2.436340	-6.987737
H	-3.975634	-5.511401	-4.426195
H	-4.514004	-4.517902	-1.810313
H	-5.490898	-6.195272	-0.657470
H	-6.165358	-8.646963	-2.194013
H	-8.059722	-6.491865	-0.946203
H	-9.844709	-8.517944	0.287970
H	-10.974960	-6.806363	1.606443
H	3.039631	-1.127631	-3.474967
H	1.355758	-2.865523	-1.572350
H	2.304749	-1.440822	-1.101191
H	0.037823	-1.270516	-3.054201
H	0.677883	0.194164	0.426149
H	1.583323	2.606265	-1.371840
H	1.943070	2.555680	2.843971
H	1.986365	1.070028	1.943082
H	4.582435	-2.921971	-3.439990
H	4.253932	-2.528055	-1.811314
H	3.631566	-3.916084	-2.536538
H	-8.779752	-4.809188	0.781808
H	-10.472001	-4.796652	0.186525

H	-10.100664	-4.501125	1.904306
H	-7.964268	-6.795663	2.250204
H	-9.106627	-8.093768	2.687153
H	-9.272490	-6.459966	3.392384
H	8.804588	3.104674	-3.897812
H	9.776817	2.563027	6.057972
H	3.049393	-8.528566	-1.018060
H	7.077760	-4.929013	-6.968218
H	-4.783044	-7.578903	3.280710
H	-3.371973	-2.918677	7.802503
H	-5.912627	-4.212500	-2.858079
H	-10.430941	-7.174103	-0.718539
H	-5.353076	-8.622642	-0.622313
H	-0.469698	8.460873	5.834521
H	2.759519	4.504156	-4.471504
H	-4.375718	5.389989	-3.179609
H	-7.186734	9.534330	-1.695201
H	-4.540362	8.297546	3.927767
H	-2.941541	0.063226	-5.568099
H	-2.489783	1.240713	6.954931
O	-0.203101	-2.513429	3.084576
H	0.066258	-3.442666	3.135564
H	0.564388	-2.023048	2.718550
O	1.813759	-0.718183	2.459089
H	1.866170	-0.495454	3.425928
H	2.758751	-0.847132	2.208880
O	4.499054	-0.653089	1.975078
H	4.841907	-1.094918	2.772922
H	4.740089	-1.180447	1.165399
O	5.016805	-1.902796	-0.363553
H	5.661735	-2.684659	-0.292803
H	5.596293	-1.245887	-0.829419
O	6.707004	-0.654986	-2.004477
H	7.524883	-0.160344	-1.777191
H	7.044430	-1.570373	-2.200784
O	4.377665	0.766233	-2.806509
H	3.884613	0.755188	-1.969151
H	5.215475	0.295876	-2.619497
O	5.069171	1.754801	3.164838
H	6.014179	1.993071	3.026057
H	4.847666	1.080949	2.479820
O	7.483486	2.809209	2.668581
H	7.924436	3.545415	3.113369
H	8.214931	2.277062	2.233782

O	2.121565	-0.024091	5.076054
H	3.087020	-0.147949	5.208847
H	1.934805	0.940128	5.096784
O	4.889342	-0.403160	4.783628
H	5.692749	-0.452606	5.331953
H	4.968304	0.475182	4.339839
O	-0.157750	-1.677506	5.795955
H	0.696332	-1.210780	5.751599
H	-0.311737	-1.930366	4.859785

^{5}**Re**$_{B}$

Element	X	Y	Z
C	4.492747	3.124693	-3.650533
H	4.828978	4.161266	-3.817677
C	3.010384	3.126094	-3.259666
H	2.440230	3.625798	-4.068044
H	2.883773	3.734811	-2.342690
O	2.605508	1.796629	-3.071080
H	1.685050	1.760149	-2.740846
C	5.347901	2.524867	-2.550033
O	5.376196	3.044573	-1.417826
N	6.043350	1.416788	-2.852564
H	5.973018	1.041155	-3.793460
C	6.718520	0.562140	-1.889406
H	6.874416	1.115115	-0.958091
C	5.865894	-0.703052	-1.550024
H	6.401150	-1.225917	-0.737628
C	4.468691	-0.356236	-1.079625
H	3.887961	0.135494	-1.872911
H	4.529068	0.313494	-0.213260
H	3.947441	-1.267747	-0.756711
O	5.772581	-1.564429	-2.695872
H	6.540415	-1.397231	-3.286005
C	8.024719	0.033943	-2.475321
O	8.000665	-0.390248	-3.637870
N	9.071602	-0.114930	-1.642696
H	9.008990	0.254313	-0.687642
C	10.110054	-1.089154	-1.962657
H	10.041387	-1.242711	-3.043364
C	11.540807	-0.611918	-1.614777
H	11.479129	0.217940	-0.893403
H	12.092740	-1.423284	-1.115165
C	12.334392	-0.152151	-2.841613

H	13.281277	0.318304	-2.529161
H	11.772387	0.625983	-3.389390
C	12.631964	-1.287919	-3.826003
O	12.000948	-2.340699	-3.831006
N	13.623538	-1.045228	-4.713019
H	13.834615	-1.750512	-5.411165
H	14.161947	-0.188650	-4.709459
C	9.752665	-2.432672	-1.286382
O	9.278262	-2.482370	-0.146275
N	9.975387	-3.508389	-2.057982
H	10.497246	-3.336432	-2.919917
C	9.698411	-4.887453	-1.668232
H	9.856659	-4.983875	-0.582542
C	8.284551	-5.377967	-2.047225
H	8.002606	-4.926828	-3.014209
H	8.330345	-6.466744	-2.221852
C	7.210937	-5.101098	-0.983119
H	7.452318	-5.689277	-0.082345
H	7.249803	-4.046932	-0.659936
C	5.777997	-5.450071	-1.400011
H	5.761550	-6.409138	-1.947605
H	5.150003	-5.599425	-0.505992
N	5.210146	-4.401517	-2.243316
H	5.676924	-3.492780	-2.277203
C	3.994381	-4.425521	-2.820263
N	3.203757	-5.495858	-2.720837
H	2.275697	-5.479938	-3.149840
H	3.345203	-6.210707	-1.991434
N	3.616050	-3.357665	-3.539318
H	2.623755	-3.227954	-3.783080
H	4.213289	-2.533290	-3.493827
C	7.227627	-2.296431	2.831934
H	7.560572	-2.786316	1.904620
C	5.703617	-2.153988	2.817551
H	5.306606	-3.137425	2.485722
C	5.082147	-1.855760	4.184071
H	5.278020	-2.680466	4.888409
H	3.990730	-1.748135	4.083002
H	5.490605	-0.932730	4.615667
O	5.399933	-1.164326	1.853122
H	4.436835	-0.987801	1.876691
C	8.005896	-1.000142	2.999080
O	7.700707	-0.092588	3.779391
N	9.153788	-0.941316	2.277043

H	9.293398	-1.584003	1.490658
C	10.110691	0.105086	2.505534
H	10.293667	0.214031	3.586759
C	9.724452	1.468251	1.922690
O	9.079190	1.557925	0.865088
N	10.201052	2.524948	2.597466
H	10.666991	2.334454	3.479564
C	10.126875	3.925446	2.189028
H	9.581046	3.968815	1.237442
C	9.459284	4.816370	3.251405
H	8.400992	4.525097	3.347585
H	9.932660	4.609176	4.228259
C	9.581453	6.292159	2.934358
C	10.788406	6.975044	3.172396
H	11.634203	6.443301	3.620149
C	10.935477	8.324983	2.867618
H	11.870766	8.853463	3.062006
C	9.860783	9.037565	2.311455
O	10.051847	10.349379	2.037047
H	9.239443	10.736766	1.685147
C	8.650043	8.372842	2.072214
H	7.802933	8.922814	1.649702
C	8.516242	7.015685	2.380085
H	7.557780	6.522190	2.195713
C	-3.395887	0.624951	4.790789
H	-3.563482	1.328405	3.956222
C	-1.907559	0.273137	4.922767
H	-1.830250	-0.495352	5.712527
C	-1.356714	-0.332830	3.624727
H	-0.286087	-0.578166	3.705890
H	-1.884309	-1.265190	3.379090
H	-1.475080	0.366811	2.778346
C	-1.118226	1.512797	5.387882
H	-1.625577	1.937129	6.271769
H	-1.174452	2.296621	4.609425
C	0.346478	1.252054	5.747987
H	0.814897	2.161329	6.160493
H	0.941824	0.929962	4.877862
H	0.437462	0.465095	6.515128
C	-4.340110	-0.565341	4.638818
O	-4.011118	-1.716385	4.941688
N	-5.583232	-0.239308	4.220346
H	-5.788427	0.712003	3.899174
C	-6.671079	-1.190803	4.128716

H	-6.319991	-2.099701	4.648028
C	-7.931890	-0.659377	4.816791
H	-8.754566	-1.389914	4.794613
H	-8.271585	0.277469	4.349834
H	-7.707060	-0.446058	5.871417
C	-6.841277	-1.659755	2.671559
O	-5.853886	-1.926730	1.981549
N	-8.090588	-1.845342	2.198994
H	-8.914432	-1.686835	2.786771
C	-8.325887	-2.625568	0.993532
H	-7.831290	-3.602724	1.116002
C	-9.838282	-2.866577	0.803245
H	-10.318172	-1.918595	0.516263
H	-9.982531	-3.589626	-0.011473
C	-10.502791	-3.323347	2.104517
O	-10.649404	-2.518670	3.023897
N	-10.878457	-4.614214	2.175734
H	-10.546927	-5.312039	1.508626
H	-11.264444	-4.935925	3.058045
C	-7.729109	-1.949766	-0.262889
O	-7.906819	-0.763254	-0.516186
N	-7.040076	-2.789032	-1.072137
C	-6.333826	-2.358689	-2.262062
C	-5.999786	-3.606809	-3.100667
C	-5.051882	-1.546842	-1.921184
O	-6.105145	-4.740275	-2.624787
C	-4.003396	-2.321616	-1.191894
C	-3.018768	-3.159022	-1.664849
N	-3.860495	-2.327718	0.180798
C	-2.833262	-3.128553	0.511734
N	-2.307517	-3.651568	-0.593778
H	-6.910654	-3.763347	-0.814165
H	-6.986293	-1.697207	-2.853771
H	-4.635696	-1.160971	-2.860531
H	-5.369383	-0.677333	-1.326970
H	-2.796593	-3.434892	-2.690477
H	-2.544495	-3.326896	1.547157
H	-4.522360	-1.936776	0.869241
N	-5.582139	-3.354191	-4.359015
H	-5.430964	-2.378682	-4.661213
C	-5.196604	-4.408232	-5.260410
H	-5.601241	-5.357647	-4.876670
C	-3.674118	-4.555529	-5.410095
O	-2.890367	-3.888365	-4.737160

N	-3.255034	-5.469143	-6.312499
C	-1.840354	-5.724350	-6.584318
C	-1.300783	-7.029540	-5.982430
C	-1.478830	-7.170680	-4.470212
C	-0.629199	-6.242201	-3.595754
O	0.428708	-5.769622	-4.031988
O	-1.105823	-6.084347	-2.409857
H	-3.949996	-5.992571	-6.835246
H	-1.285442	-4.865206	-6.186399
H	-1.787888	-7.888638	-6.475304
H	-0.229273	-7.089804	-6.230395
H	-1.199447	-8.196843	-4.169184
H	-2.533099	-7.048024	-4.177299
C	-7.231060	3.117387	-4.072307
H	-8.024590	2.820250	-4.777474
C	-6.198232	1.990407	-3.924770
H	-6.710540	1.062916	-3.620342
H	-5.497062	2.253285	-3.116303
C	-5.415855	1.730852	-5.220057
H	-4.782233	2.598047	-5.464203
H	-6.120617	1.587740	-6.056060
C	-4.590815	0.451992	-5.135978
O	-5.174408	-0.643719	-5.079610
N	-3.262115	0.587192	-5.116815
H	-2.788206	1.486394	-4.996043
H	-2.655646	-0.234466	-5.025309
C	-7.809599	3.550177	-2.735063
O	-7.108517	4.043351	-1.856795
N	-9.145976	3.341414	-2.546768
H	-9.696372	3.012259	-3.331037
C	-9.848280	3.924008	-1.414767
H	-9.812473	5.024519	-1.437195
C	-9.310055	3.528409	-0.036940
O	-9.373499	4.321920	0.894657
N	-8.795289	2.281869	0.092962
H	-8.748567	1.651942	-0.701751
C	-8.274404	1.810764	1.356411
H	-8.997441	2.013352	2.163094
C	-6.963807	2.440208	1.835701
O	-6.518805	2.121294	2.939543
N	-6.379638	3.362038	1.043368
H	-6.769698	3.559640	0.120242
C	-5.299445	4.213564	1.518949
H	-4.626215	3.605429	2.140606

C	-4.527058	4.829496	0.339931
H	-5.255714	5.333414	-0.316204
H	-3.862736	5.609141	0.742479
C	-3.692199	3.835344	-0.488237
H	-4.329575	2.963903	-0.726476
C	-3.282470	4.479322	-1.818418
H	-2.654943	5.371212	-1.646788
H	-2.714327	3.772823	-2.443914
H	-4.170734	4.798354	-2.386420
C	-2.462881	3.336391	0.284298
H	-1.834160	4.186793	0.599273
H	-2.742758	2.779895	1.193596
H	-1.846429	2.669112	-0.339481
C	-5.779470	5.331189	2.471128
O	-4.949986	5.933189	3.155983
N	-7.096497	5.613154	2.488939
H	-7.743753	5.077607	1.909453
C	-7.656607	6.686566	3.293167
H	-6.920546	6.931175	4.071499
C	-8.008619	7.944261	2.471391
H	-8.499053	8.658796	3.154018
H	-8.757154	7.669844	1.710123
C	-6.817602	8.600903	1.807894
C	-5.924730	9.388776	2.553033
H	-6.111679	9.553719	3.618125
C	-4.804261	9.970234	1.954376
H	-4.130644	10.590773	2.551429
C	-4.552688	9.770539	0.591051
H	-3.684827	10.236361	0.116679
C	-5.434081	8.990487	-0.163666
H	-5.253280	8.837001	-1.230765
C	-6.554770	8.412755	0.441824
H	-7.241656	7.808549	-0.157629
C	-8.645463	-6.670373	-1.649504
H	-7.887573	-6.274578	-2.338231
C	-8.306610	-6.423165	-0.194011
O	-9.163762	-6.432577	0.683772
N	-6.980294	-6.218228	0.085669
C	-6.484496	-6.046271	1.445567
C	-5.445610	-7.103795	1.881567
C	-4.190852	-7.050821	1.068455
C	-3.098318	-6.218735	1.143241
N	-4.010305	-7.770587	-0.102450
C	-2.867449	-7.359401	-0.692687

N	-2.298675	-6.406944	0.029911
H	-6.352789	-6.063695	-0.701380
H	-7.363438	-6.099855	2.102388
H	-5.896673	-8.108978	1.838111
H	-5.194288	-6.911054	2.935794
H	-2.871457	-5.493954	1.923047
H	-2.490215	-7.735993	-1.637175
H	-4.623933	-8.498818	-0.450968
Fe	-0.821772	-5.063081	-0.736551
O	0.217883	-3.953236	-1.314046
C	0.544900	-5.715147	1.273239
O	-0.310621	-4.802175	1.379430
C	1.388561	-6.157548	2.438178
H	2.351096	-6.548191	2.080980
H	1.538194	-5.328058	3.141391
C	2.834627	3.771139	3.622652
C	2.133678	5.129899	3.874346
O	2.696546	5.961514	4.555340
S	0.522543	5.309642	3.195603
C	0.062557	6.942785	3.852119
C	2.610296	3.137898	2.242113
N	2.992798	1.733488	2.252348
C	2.411863	0.704330	1.579946
O	2.737148	-0.465100	1.806785
H	3.841498	1.470450	2.771924
C	1.444896	1.017681	0.483794
C	0.837682	0.010980	-0.159021
C	0.035260	0.210363	-1.401768
O	0.030334	1.302609	-2.004517
N	-0.630630	-0.871933	-1.814217
H	-1.103867	-0.914834	-2.730265
H	-0.539848	-1.752050	-1.310990
N	4.279587	3.996413	3.907039
H	4.750596	4.553362	3.107752
H	4.358476	4.573787	4.754260
H	2.441002	3.078953	4.386801
H	-0.030698	6.890271	4.944691
H	-0.905837	7.167139	3.373873
H	3.141892	3.721481	1.469813
H	1.538989	3.216160	2.015322
H	1.315169	2.041730	0.126044
H	0.982482	-1.009740	0.207926
H	4.840156	3.106548	3.974575
H	0.861250	-6.968227	2.967672

O	0.672593	-6.295377	0.140111
H	-6.034512	-5.045589	1.561338
H	-9.625604	-6.224513	-1.867546
H	-8.745237	-7.757879	-1.802666
H	-1.692561	-5.734852	-7.676008
H	-5.636684	-4.233570	-6.257136
H	-3.730020	1.162102	5.696759
H	10.451344	-5.514777	-2.167362
H	4.621888	2.571531	-4.592993
H	-8.567693	6.314507	3.788995
H	-10.906027	3.623816	-1.470281
H	-6.743499	4.012623	-4.493897
H	11.149501	4.290349	1.992382
H	11.065162	-0.185968	2.035733
H	7.531330	-2.956184	3.663737
H	0.830166	7.676267	3.574157
O	3.122153	-7.256063	-0.517263
H	3.125421	-8.222360	-0.545259
H	2.194289	-7.005695	-0.289632
O	5.573365	5.160560	1.900657
H	5.015203	5.362959	1.111291
H	6.150170	4.425083	1.588489
O	4.000016	5.124773	-0.313360
H	3.829552	5.868513	-0.906634
H	4.478777	4.447944	-0.849060
O	-2.843116	-3.764716	3.511758
H	-1.977636	-3.797930	3.940632
H	-3.344179	-3.099692	4.035554
O	0.800946	-3.106027	-3.922961
H	0.629780	-4.027575	-4.222098
H	0.594266	-3.202950	-2.967870
O	-1.452093	-1.568209	-4.449782
H	-2.035516	-2.345180	-4.565606
H	-0.553238	-1.958870	-4.508945
O	-1.330597	2.495031	-4.188387
H	-0.913797	2.037633	-3.427188
H	-1.227407	3.434854	-3.994466
O	5.683311	1.753022	3.350563
H	6.030781	1.991444	2.458400
H	6.294387	1.046797	3.664675
O	6.665376	2.815462	0.988641
H	6.240140	2.803704	0.099546
H	7.587062	2.489023	0.865148
O	-2.642875	7.077758	2.212612

H	-3.061399	7.882107	1.870649
H	-3.392573	6.623817	2.659420
H	-8.137642	0.724927	1.282044

^{5}**TS1**$_{B}$

Element	X	Y	Z
C	5.083678	3.162387	-3.710714
H	5.300313	4.242740	-3.750099
C	3.606420	2.957412	-3.354120
H	2.994923	3.474479	-4.118761
H	3.399516	3.443966	-2.381460
O	3.340795	1.577039	-3.316032
H	2.393349	1.427927	-3.118832
C	5.992196	2.537349	-2.668898
O	5.932725	2.901438	-1.478049
N	6.830398	1.575382	-3.086243
H	6.836540	1.318673	-4.069549
C	7.571528	0.685820	-2.210676
H	7.767553	1.180210	-1.255128
C	6.779003	-0.631106	-1.931112
H	7.382007	-1.210506	-1.209224
C	5.400015	-0.397957	-1.352311
H	4.764615	0.150417	-2.061979
H	5.481352	0.159757	-0.410871
H	4.935537	-1.364752	-1.123319
O	6.636453	-1.382531	-3.147510
H	7.373664	-1.176066	-3.756755
C	8.860757	0.223693	-2.883607
O	8.836885	-0.000446	-4.097589
N	9.878097	-0.121074	-2.065704
H	9.766399	0.055187	-1.062470
C	10.831965	-1.143055	-2.489858
H	10.709009	-1.219745	-3.574020
C	12.310271	-0.800013	-2.180669
H	12.344433	-0.006597	-1.417828
H	12.813179	-1.676653	-1.743403
C	13.089581	-0.343632	-3.417798
H	14.084027	0.031711	-3.125302
H	12.572426	0.504188	-3.902821
C	13.250668	-1.447385	-4.467713
O	12.529348	-2.440673	-4.498612
N	14.226404	-1.245409	-5.381454
H	14.347721	-1.927838	-6.122365

H	14.836467	-0.438583	-5.359337
C	10.400966	-2.491404	-1.871739
O	9.979394	-2.563097	-0.712371
N	10.493909	-3.537513	-2.707593
H	10.998870	-3.363165	-3.579481
C	10.127340	-4.910620	-2.368782
H	10.381007	-5.094467	-1.312477
C	8.644441	-5.254269	-2.626333
H	8.320261	-4.741241	-3.548250
H	8.571826	-6.334699	-2.839653
C	7.708016	-4.921857	-1.453963
H	7.978142	-5.564730	-0.599758
H	7.876917	-3.889566	-1.108098
C	6.213217	-5.112343	-1.725420
H	6.030744	-6.070608	-2.245541
H	5.666819	-5.162122	-0.769353
N	5.688459	-4.013888	-2.532986
H	6.287360	-3.207367	-2.722687
C	4.410142	-3.875084	-2.921243
N	3.493689	-4.785777	-2.581560
H	2.536976	-4.674702	-2.927604
H	3.598179	-5.324623	-1.709913
N	4.080951	-2.830735	-3.699315
H	3.101669	-2.501756	-3.721732
H	4.806642	-2.138027	-3.867148
C	7.805219	-3.080691	2.198764
H	8.205994	-3.416022	1.231032
C	6.275514	-3.124822	2.157711
H	6.008509	-4.120358	1.745340
C	5.603874	-3.012403	3.528447
H	5.901832	-3.851566	4.177724
H	4.509772	-3.052792	3.412681
H	5.873387	-2.076820	4.034606
O	5.862510	-2.110667	1.260319
H	4.886523	-2.094289	1.207460
C	8.440370	-1.741515	2.544999
O	8.000796	-0.952981	3.388882
N	9.622843	-1.508214	1.922005
H	9.858930	-2.026408	1.071624
C	10.469685	-0.418233	2.320415
H	10.545398	-0.389972	3.419233
C	10.030119	0.959837	1.815245
O	9.410996	1.089502	0.746673
N	10.419817	1.991602	2.579318

H	10.882336	1.769780	3.456043
C	10.266194	3.410125	2.262644
H	9.663945	3.485020	1.348047
C	9.619914	4.198597	3.414107
H	8.591628	3.832936	3.565285
H	10.173306	3.977270	4.344972
C	9.618565	5.693131	3.170628
C	10.794560	6.446767	3.339587
H	11.711682	5.954226	3.678363
C	10.822526	7.817905	3.102935
H	11.734647	8.400975	3.243938
C	9.655551	8.480404	2.689017
O	9.732793	9.815288	2.477457
H	8.865997	10.163138	2.229420
C	8.474406	7.744580	2.519955
H	7.556773	8.254581	2.209441
C	8.460684	6.366972	2.757690
H	7.524791	5.815243	2.630379
C	-4.805315	1.757570	5.141021
H	-5.035325	2.464339	4.324985
C	-3.301587	1.734460	5.447974
H	-3.151125	0.986680	6.247242
C	-2.488160	1.283754	4.227445
H	-1.408369	1.266520	4.443684
H	-2.776216	0.268870	3.917720
H	-2.641749	1.967603	3.375634
C	-2.856715	3.108734	5.987588
H	-3.546668	3.403205	6.797814
H	-2.981171	3.871284	5.197457
C	-1.425968	3.156598	6.528374
H	-1.201424	4.143445	6.963937
H	-0.678226	2.969024	5.740482
H	-1.269818	2.403908	7.319836
C	-5.434957	0.396926	4.867324
O	-4.916673	-0.659274	5.246759
N	-6.646139	0.437453	4.264884
H	-6.994978	1.299196	3.844187
C	-7.528515	-0.707804	4.180490
H	-7.060496	-1.485641	4.809543
C	-8.919001	-0.371327	4.732072
H	-9.568891	-1.258043	4.781816
H	-9.403996	0.399047	4.114630
H	-8.818148	0.017301	5.755551
C	-7.502792	-1.332920	2.775753

O	-6.451797	-1.413982	2.132166
N	-8.645237	-1.892508	2.331374
H	-9.505262	-1.850517	2.882922
C	-8.649607	-2.842507	1.233950
H	-7.940350	-3.651429	1.473464
C	-10.054137	-3.460555	1.069494
H	-10.737934	-2.693740	0.675195
H	-9.997297	-4.283423	0.343360
C	-10.626615	-3.916940	2.413757
O	-11.009120	-3.077387	3.226684
N	-10.656893	-5.244487	2.641891
H	-10.143016	-5.907607	2.060860
H	-10.977081	-5.549786	3.555592
C	-8.193857	-2.192376	-0.087565
O	-8.633478	-1.118680	-0.480650
N	-7.298975	-2.922999	-0.795328
C	-6.711214	-2.450316	-2.029561
C	-6.087146	-3.653446	-2.761343
C	-5.691762	-1.303383	-1.787362
O	-5.984426	-4.750330	-2.207381
C	-4.481034	-1.695772	-1.003458
C	-3.288373	-2.237700	-1.427735
N	-4.360661	-1.571283	0.366967
C	-3.143260	-2.010125	0.736563
N	-2.465544	-2.426242	-0.333580
H	-6.968186	-3.820164	-0.452972
H	-7.510797	-2.034695	-2.664954
H	-5.377809	-0.914802	-2.765588
H	-6.236261	-0.496047	-1.275800
H	-3.002531	-2.524802	-2.435930
H	-2.822840	-2.033154	1.780424
H	-5.113751	-1.318262	1.030436
N	-5.701229	-3.416793	-4.033177
H	-5.642254	-2.454151	-4.392426
C	-5.121067	-4.445946	-4.854447
H	-5.380755	-5.423128	-4.418662
C	-3.592051	-4.358183	-4.949348
O	-2.944041	-3.624813	-4.207481
N	-3.017451	-5.158660	-5.872740
C	-1.568686	-5.246710	-6.054239
C	-0.905059	-6.361399	-5.232580
C	-1.061315	-6.232903	-3.713553
C	-0.280856	-5.079302	-3.082738
O	0.757961	-4.686708	-3.636542

O	-0.749818	-4.636382	-1.973167
H	-3.612724	-5.749646	-6.443217
H	-1.144684	-4.264782	-5.801687
H	-1.314917	-7.335554	-5.550258
H	0.167681	-6.369488	-5.480485
H	-0.670479	-7.151943	-3.238275
H	-2.115228	-6.152743	-3.410744
C	-7.275549	3.017858	-4.662769
H	-8.010348	2.739518	-5.435989
C	-6.323152	1.851661	-4.367239
H	-6.907313	0.945451	-4.139545
H	-5.742037	2.096576	-3.464021
C	-5.364776	1.537261	-5.521841
H	-4.685428	2.385605	-5.703208
H	-5.938700	1.376675	-6.451125
C	-4.584577	0.246711	-5.276160
O	-5.204396	-0.809470	-5.082123
N	-3.249515	0.337207	-5.290171
H	-2.768616	1.233750	-5.319994
H	-2.631334	-0.476997	-5.155293
C	-7.959296	3.538065	-3.406802
O	-7.322649	3.824323	-2.397313
N	-9.317897	3.671459	-3.449024
H	-9.790799	3.508396	-4.330525
C	-10.034071	4.396775	-2.409518
H	-9.694959	5.442090	-2.338236
C	-9.891079	3.825108	-0.993814
O	-9.944209	4.569643	-0.023596
N	-9.719456	2.484503	-0.891299
H	-9.662374	1.931160	-1.738170
C	-9.545866	1.828539	0.389497
H	-10.389040	2.055272	1.060382
C	-8.289932	2.220138	1.168307
O	-8.188800	1.884538	2.347158
N	-7.345382	2.947126	0.529483
H	-7.468750	3.208574	-0.449366
C	-6.231530	3.526138	1.263007
H	-5.728626	2.731278	1.833084
C	-5.227086	4.198966	0.311365
H	-5.783133	4.890901	-0.342513
H	-4.544627	4.808141	0.924598
C	-4.386493	3.236628	-0.548363
H	-5.074269	2.546652	-1.069820
C	-3.630537	4.030207	-1.622454

H	-2.966303	4.780772	-1.160136
H	-3.007279	3.366957	-2.245086
H	-4.333179	4.561900	-2.283352
C	-3.420542	2.404920	0.304494
H	-2.744860	3.061334	0.878295
H	-3.947487	1.761092	1.025816
H	-2.801956	1.749915	-0.327847
C	-6.673276	4.534314	2.342605
O	-5.930527	4.739603	3.308327
N	-7.837914	5.179819	2.151468
H	-8.432500	4.924799	1.361148
C	-8.354776	6.166867	3.085952
H	-7.797551	6.049322	4.025757
C	-8.243723	7.617287	2.574201
H	-8.747433	8.265118	3.311614
H	-8.814005	7.708737	1.635292
C	-6.823401	8.094019	2.360468
C	-6.006992	8.428894	3.453690
H	-6.411873	8.367396	4.467939
C	-4.687506	8.848598	3.265950
H	-4.075878	9.118450	4.130959
C	-4.155143	8.936590	1.973231
H	-3.127729	9.278458	1.822765
C	-4.956528	8.607995	0.875696
H	-4.555362	8.684850	-0.138275
C	-6.277303	8.191328	1.071058
H	-6.899860	7.945407	0.206137
C	-7.961923	-7.124607	-0.965571
H	-7.337103	-6.620491	-1.714497
C	-7.704256	-6.632506	0.443726
O	-8.534170	-6.757624	1.338341
N	-6.475637	-6.065060	0.664353
C	-6.048158	-5.611075	1.982167
C	-4.783367	-6.316137	2.519067
C	-3.570648	-6.052757	1.684022
C	-2.681729	-5.001595	1.676758
N	-3.230092	-6.810114	0.575202
C	-2.193825	-6.207311	-0.053681
N	-1.846949	-5.101288	0.580072
H	-5.901467	-5.856571	-0.150455
H	-6.891576	-5.799781	2.660194
H	-4.970840	-7.399345	2.607112
H	-4.598771	-5.938790	3.536469
H	-2.631883	-4.181168	2.389803

H	-1.739784	-6.580129	-0.965266
H	-3.672543	-7.676840	0.290781
Fe	-0.611583	-3.465671	-0.396620
O	0.254926	-2.103333	-1.016891
C	0.836024	-3.796592	1.629888
O	-0.138357	-2.997431	1.661278
C	1.677657	-4.051668	2.849433
H	2.656704	-4.455749	2.563104
H	1.794290	-3.128533	3.431481
C	2.827244	2.392424	3.166155
C	2.016122	3.689333	3.408017
O	2.450625	4.526015	4.169127
S	0.446434	3.780196	2.613276
C	-0.172154	5.342244	3.314507
C	2.898784	1.901489	1.708787
N	3.286442	0.500962	1.638841
C	2.782536	-0.440200	0.799001
O	3.112461	-1.624793	0.873822
H	4.116667	0.213329	2.176987
C	1.936806	0.022661	-0.347516
C	0.594363	-0.136814	-0.540848
C	-0.006716	0.505540	-1.771179
O	0.662768	1.232979	-2.525160
N	-1.310423	0.281356	-1.967676
H	-1.744381	0.660631	-2.808663
H	-1.785519	-0.462025	-1.465619
N	4.182272	2.646644	3.729494
H	4.712463	3.386170	3.138863
H	4.094200	3.043720	4.671518
H	2.346248	1.604559	3.770303
H	0.000987	5.337889	4.397914
H	-1.252001	5.373791	3.097437
H	3.572343	2.562951	1.136817
H	1.903647	2.013484	1.256990
H	2.515299	0.470784	-1.164653
H	-0.069478	-0.375107	0.289967
H	4.807959	1.796975	3.712703
H	1.159460	-4.790175	3.483318
O	1.064519	-4.433263	0.548675
H	-5.861657	-4.523911	1.966928
H	-9.025507	-6.983930	-1.201890
H	-7.765181	-8.209253	-0.997017
H	-1.372718	-5.413870	-7.124360
H	-5.550729	-4.403166	-5.869533

H	-5.347523	2.156625	6.017979
H	10.767331	-5.565292	-2.977773
H	5.283451	2.741415	-4.707482
H	-9.413619	5.936521	3.286402
H	-11.103686	4.409979	-2.669288
H	-6.702163	3.872685	-5.064104
H	11.258188	3.835661	2.032597
H	11.482193	-0.600575	1.922732
H	8.171213	-3.798963	2.953725
H	0.355107	6.188088	2.854004
O	3.401191	-5.759542	0.083902
H	3.213163	-6.668483	0.355197
H	2.559977	-5.270246	0.261761
O	5.625170	4.276915	2.215418
H	5.135182	4.601823	1.421249
H	6.286442	3.655569	1.833249
O	4.288315	4.602013	-0.126532
H	4.095801	5.439080	-0.569645
H	4.870937	4.094592	-0.740425
O	-3.230331	-2.321359	3.818131
H	-2.472954	-2.200772	4.406407
H	-3.946680	-1.826745	4.277909
O	1.310501	-2.069664	-3.576168
H	1.080741	-3.013404	-3.743528
H	1.054400	-1.997818	-2.626956
O	-1.194324	-1.593839	-4.862389
H	-1.624770	-2.401456	-4.529383
H	-0.293552	-1.587556	-4.480372
O	-1.330652	2.330259	-4.220786
H	-0.486887	2.035548	-3.816039
H	-1.421965	3.252430	-3.945378
O	5.826395	0.656727	2.986106
H	6.235394	1.103017	2.207503
H	6.501708	-0.007361	3.263905
O	6.938793	2.216348	0.974393
H	6.610211	2.389116	0.060377
H	7.873008	1.918196	0.873334
O	-3.329326	5.564484	2.911857
H	-3.566309	6.502246	2.841156
H	-4.185605	5.150792	3.156728
H	-9.509497	0.740695	0.232560

^{5}**IM1**$_{B}$

Element	X	Y	Z
C	4.757175	3.563954	-3.407130
H	5.041235	4.629380	-3.412322
C	3.302551	3.430342	-2.943261
H	2.666753	4.018579	-3.634067
H	3.203424	3.882520	-1.937758
O	2.962938	2.067172	-2.942471
H	2.044967	1.946467	-2.631409
C	5.702351	2.830092	-2.474092
O	5.751520	3.123172	-1.264078
N	6.457248	1.858071	-3.011610
H	6.384530	1.671533	-4.007762
C	7.244883	0.900262	-2.256305
H	7.494130	1.318488	-1.277270
C	6.468875	-0.435293	-2.022330
H	7.094629	-1.035575	-1.337094
C	5.103022	-0.236096	-1.397645
H	4.443015	0.353963	-2.049712
H	5.207573	0.271610	-0.430269
H	4.642286	-1.213132	-1.204962
O	6.317261	-1.148332	-3.262031
H	6.986572	-0.841446	-3.907324
C	8.492930	0.511353	-3.045137
O	8.383298	0.373248	-4.268542
N	9.579538	0.148842	-2.334609
H	9.545920	0.241138	-1.313574
C	10.561471	-0.759884	-2.921694
H	10.335098	-0.776761	-3.991687
C	12.033047	-0.314126	-2.730609
H	12.079556	0.437028	-1.926929
H	12.641885	-1.168329	-2.395514
C	12.651525	0.271109	-4.003843
H	13.637765	0.710628	-3.781314
H	12.026299	1.098599	-4.385832
C	12.794212	-0.755708	-5.131302
O	12.146253	-1.798346	-5.162414
N	13.664861	-0.427629	-6.112391
H	13.766489	-1.055812	-6.902570
H	14.215882	0.420644	-6.091862
C	10.301690	-2.172795	-2.360056
O	10.012460	-2.343030	-1.172075
N	10.384629	-3.158406	-3.268420
H	10.787703	-2.895408	-4.170564

C	10.155919	-4.572797	-2.981678
H	10.554604	-4.801926	-1.980113
C	8.677870	-5.005813	-3.075259
H	8.216970	-4.495088	-3.938218
H	8.645838	-6.084384	-3.307406
C	7.870418	-4.751071	-1.792734
H	8.259845	-5.408130	-0.997407
H	8.045132	-3.724536	-1.434682
C	6.362206	-4.986457	-1.895383
H	6.146828	-5.948368	-2.394183
H	5.931595	-5.048225	-0.881740
N	5.717440	-3.908460	-2.639145
H	6.241430	-3.054639	-2.842111
C	4.417453	-3.869519	-2.973072
N	3.591469	-4.846285	-2.571953
H	2.616786	-4.817463	-2.878901
H	3.739315	-5.273039	-1.647108
N	3.979436	-2.873743	-3.754777
H	2.975584	-2.620594	-3.737448
H	4.641960	-2.125836	-3.950240
C	7.551980	-3.196674	1.906001
H	7.830278	-3.514480	0.890539
C	6.025101	-3.144197	2.014179
H	5.649137	-4.105780	1.610110
C	5.507726	-3.026799	3.451281
H	5.794301	-3.912815	4.041335
H	4.409471	-2.968542	3.447030
H	5.906585	-2.138616	3.956284
O	5.592096	-2.083316	1.183115
H	4.612843	-2.079725	1.131471
C	8.307776	-1.917281	2.239827
O	8.003115	-1.144698	3.155380
N	9.435631	-1.720023	1.516270
H	9.598979	-2.220622	0.641672
C	10.396067	-0.715687	1.887116
H	10.661141	-0.823034	2.951655
C	9.966277	0.728946	1.617230
O	9.311256	1.030508	0.605770
N	10.417515	1.625619	2.507194
H	10.890359	1.260620	3.328382
C	10.292055	3.078421	2.420211
H	9.706621	3.312327	1.521606
C	9.643275	3.687541	3.675305
H	8.600868	3.337684	3.744298

H	10.166839	3.293363	4.565382
C	9.698688	5.200906	3.686339
C	10.900470	5.869034	3.985023
H	11.794686	5.291054	4.239739
C	10.983257	7.258248	3.983067
H	11.915457	7.772896	4.223877
C	9.846854	8.026209	3.681642
O	9.976839	9.373779	3.697800
H	9.126910	9.791375	3.505161
C	8.640631	7.376107	3.386562
H	7.746243	7.966271	3.162145
C	8.571744	5.979687	3.388563
H	7.616032	5.496557	3.166310
C	-4.932879	1.922454	5.178721
H	-5.138239	2.643169	4.368524
C	-3.438018	1.882268	5.522315
H	-3.309521	1.096999	6.288726
C	-2.601181	1.489771	4.297338
H	-1.524640	1.466666	4.527813
H	-2.880569	0.489296	3.935957
H	-2.744362	2.213074	3.476645
C	-2.995297	3.225815	6.135233
H	-3.731705	3.521001	6.903176
H	-3.038648	4.011760	5.359528
C	-1.607379	3.206311	6.779496
H	-1.366436	4.181053	7.233230
H	-0.813255	2.977957	6.050236
H	-1.548429	2.447978	7.578494
C	-5.559951	0.568984	4.870446
O	-5.073299	-0.492786	5.276551
N	-6.739940	0.619830	4.209855
H	-7.051094	1.479721	3.758506
C	-7.639806	-0.509442	4.108119
H	-7.216945	-1.281363	4.775619
C	-9.046388	-0.132066	4.589239
H	-9.714763	-1.004844	4.639687
H	-9.490619	0.626627	3.928142
H	-8.981051	0.286786	5.603619
C	-7.566413	-1.172381	2.722651
O	-6.497347	-1.265928	2.111899
N	-8.693456	-1.756763	2.268592
H	-9.567376	-1.697747	2.795947
C	-8.663151	-2.758493	1.219738
H	-7.954005	-3.548867	1.515553

C	-10.058641	-3.397108	1.051993
H	-10.737666	-2.657978	0.600709
H	-9.976466	-4.256245	0.371706
C	-10.663235	-3.787309	2.403169
O	-11.075426	-2.909650	3.159297
N	-10.688181	-5.100958	2.701709
H	-10.152719	-5.789713	2.172428
H	-11.030907	-5.359625	3.621624
C	-8.180447	-2.172966	-0.121456
O	-8.604243	-1.116371	-0.573379
N	-7.279762	-2.944043	-0.777723
C	-6.678634	-2.543549	-2.030511
C	-6.053227	-3.788549	-2.685153
C	-5.659254	-1.387449	-1.845025
O	-5.965137	-4.854296	-2.072689
C	-4.473341	-1.723666	-0.999848
C	-3.269173	-2.294292	-1.342321
N	-4.389754	-1.480142	0.357868
C	-3.181198	-1.875204	0.794502
N	-2.472525	-2.377751	-0.217327
H	-6.961343	-3.826325	-0.387706
H	-7.471005	-2.164684	-2.697528
H	-5.315763	-1.067126	-2.838319
H	-6.212044	-0.543986	-1.405856
H	-2.969391	-2.685406	-2.312332
H	-2.890334	-1.817139	1.845744
H	-5.162915	-1.191431	0.981520
N	-5.651837	-3.628820	-3.965137
H	-5.565718	-2.690293	-4.374198
C	-5.093076	-4.717969	-4.720067
H	-5.453629	-5.664775	-4.289476
C	-3.558717	-4.740748	-4.724377
O	-2.899414	-3.936278	-4.069617
N	-2.995824	-5.704313	-5.484015
C	-1.549338	-5.883299	-5.607547
C	-0.950819	-6.866297	-4.591989
C	-1.092241	-6.452670	-3.123017
C	-0.267534	-5.231430	-2.722592
O	0.749810	-4.955168	-3.378655
O	-0.670314	-4.575578	-1.695094
H	-3.603587	-6.345277	-5.983076
H	-1.080863	-4.893774	-5.514133
H	-1.418288	-7.856361	-4.729421
H	0.118200	-6.981604	-4.828483

H	-0.732164	-7.283943	-2.488269
H	-2.139933	-6.276451	-2.839421
C	-7.018099	2.809266	-4.920486
H	-7.771461	2.511519	-5.668334
C	-6.086229	1.638287	-4.585693
H	-6.684236	0.761880	-4.288528
H	-5.472323	1.918134	-3.715030
C	-5.173843	1.234382	-5.749407
H	-4.489968	2.058661	-6.008999
H	-5.781861	1.026099	-6.647241
C	-4.401592	-0.049883	-5.451416
O	-5.017078	-1.077198	-5.138347
N	-3.065980	0.010956	-5.560046
H	-2.591549	0.896340	-5.705293
H	-2.458614	-0.806706	-5.387730
C	-7.673599	3.404690	-3.682147
O	-7.028247	3.676803	-2.674687
N	-9.019972	3.627620	-3.744100
H	-9.494122	3.469621	-4.625874
C	-9.695280	4.432829	-2.736894
H	-9.280018	5.451641	-2.687716
C	-9.618001	3.892271	-1.304348
O	-9.649329	4.665288	-0.355614
N	-9.525698	2.547679	-1.163261
H	-9.486886	1.969863	-1.994570
C	-9.422762	1.917710	0.138015
H	-10.278304	2.192489	0.774340
C	-8.178934	2.279390	0.950257
O	-8.144285	1.997619	2.146311
N	-7.167182	2.918063	0.318199
H	-7.241666	3.146198	-0.673603
C	-6.042882	3.452773	1.070878
H	-5.604885	2.646768	1.678663
C	-4.973552	4.043399	0.135559
H	-5.463983	4.786144	-0.515797
H	-4.251875	4.591241	0.762363
C	-4.209643	3.026488	-0.733482
H	-4.949355	2.384329	-1.243857
C	-3.423070	3.772244	-1.819103
H	-2.719824	4.492696	-1.366832
H	-2.834437	3.075787	-2.437343
H	-4.105182	4.331375	-2.479139
C	-3.284909	2.128401	0.097312
H	-2.500639	2.724781	0.593087

H	-3.829638	1.580102	0.881389
H	-2.793484	1.378610	-0.543732
C	-6.459641	4.519116	2.104559
O	-5.739324	4.713321	3.089589
N	-7.577017	5.224312	1.852675
H	-8.160517	4.976196	1.052007
C	-8.072056	6.263736	2.741130
H	-7.543258	6.153666	3.698076
C	-7.885847	7.689650	2.184766
H	-8.380100	8.382915	2.886385
H	-8.427652	7.772654	1.228376
C	-6.441487	8.098931	1.993926
C	-5.639116	8.433689	3.097602
H	-6.071611	8.421650	4.102202
C	-4.298422	8.790798	2.932062
H	-3.697311	9.060847	3.804272
C	-3.730653	8.815747	1.651739
H	-2.685887	9.108389	1.518183
C	-4.517535	8.486340	0.543953
H	-4.087976	8.513961	-0.460890
C	-5.859461	8.131948	0.717024
H	-6.470211	7.885097	-0.156034
C	-7.861937	-7.206926	-0.693248
H	-7.233380	-6.734902	-1.459730
C	-7.660023	-6.606983	0.682887
O	-8.519940	-6.674602	1.554928
N	-6.445229	-6.010474	0.904061
C	-6.065997	-5.462440	2.200358
C	-4.813671	-6.114659	2.825732
C	-3.571488	-5.884921	2.023838
C	-2.720944	-4.804235	1.960468
N	-3.142996	-6.731592	1.014622
C	-2.092540	-6.151074	0.387215
N	-1.821983	-4.975506	0.925262
H	-5.842208	-5.857915	0.098542
H	-6.929803	-5.614365	2.861717
H	-4.990295	-7.193060	2.973408
H	-4.673711	-5.673168	3.824395
H	-2.738734	-3.921701	2.597754
H	-1.570220	-6.599944	-0.450174
H	-3.535381	-7.640861	0.797125
Fe	-0.601477	-3.358860	-0.155179
O	0.308076	-1.840602	-0.936712
C	0.846333	-3.593159	1.853962

O	-0.127172	-2.784925	1.825662
C	1.641764	-3.802476	3.107697
H	2.610759	-4.260666	2.877424
H	1.780146	-2.850331	3.635714
C	2.886857	2.208607	3.422564
C	2.092692	3.489841	3.781398
O	2.529622	4.244235	4.622896
S	0.542227	3.691579	2.967347
C	-0.053084	5.210581	3.771697
C	2.908422	1.840295	1.926153
N	3.219515	0.443080	1.686791
C	2.530986	-0.439631	0.883253
O	2.839288	-1.642625	0.873771
H	4.098514	0.090476	2.083589
C	1.515451	0.132112	0.002261
C	0.293246	-0.488538	-0.605561
C	-0.143548	0.388712	-1.830238
O	0.308758	1.530637	-2.024898
N	-1.080219	-0.154068	-2.601626
H	-1.418344	0.357097	-3.416467
H	-1.336912	-1.125421	-2.489587
N	4.255704	2.410009	3.968315
H	4.761598	3.204926	3.437742
H	4.200834	2.704316	4.949182
H	2.413515	1.378285	3.973435
H	-0.002452	5.088224	4.860686
H	-1.097190	5.327078	3.438861
H	3.594547	2.526747	1.399731
H	1.907983	2.050698	1.531957
H	1.606818	1.190181	-0.245365
H	-0.522927	-0.294313	0.135286
H	4.884141	1.570156	3.829778
H	1.074518	-4.478231	3.769112
O	1.086016	-4.277593	0.806395
H	-5.889467	-4.376727	2.117283
H	-8.922448	-7.119967	-0.965750
H	-7.627760	-8.283429	-0.642248
H	-1.342388	-6.240243	-6.627885
H	-5.447446	-4.664505	-5.763161
H	-5.495662	2.304464	6.050629
H	10.752238	-5.141939	-3.709239
H	4.851766	3.184304	-4.435616
H	-9.144565	6.086194	2.922184
H	-10.756841	4.518899	-3.015028

H	-6.434585	3.631700	-5.372752
H	11.295815	3.513095	2.274524
H	11.309383	-0.890998	1.295808
H	7.940802	-3.969674	2.592421
H	0.566995	6.060984	3.459254
O	3.546529	-5.440480	0.234137
H	3.421219	-6.310769	0.636937
H	2.703085	-4.965793	0.425308
O	5.646647	4.179665	2.552113
H	5.134751	4.592345	1.814379
H	6.293304	3.604425	2.084097
O	4.254078	4.786607	0.303668
H	4.106277	5.671644	-0.055427
H	4.784880	4.303237	-0.372463
O	-3.305011	-2.091429	3.894338
H	-2.561529	-1.932518	4.491254
H	-4.046917	-1.617477	4.336623
O	1.226339	-2.332654	-3.488957
H	1.030410	-3.301113	-3.499760
H	1.024371	-2.097744	-2.550901
O	-1.152516	-2.020892	-4.991030
H	-1.615606	-2.791744	-4.613885
H	-0.268025	-1.999765	-4.569377
O	-1.196855	2.273464	-4.305965
H	-0.566871	2.245009	-3.554186
H	-1.633777	3.130773	-4.216675
O	5.871017	0.561056	2.953927
H	6.253527	1.072941	2.202956
H	6.540633	-0.136855	3.143080
O	6.926379	2.267188	1.051104
H	6.542926	2.511165	0.175782
H	7.843421	1.952615	0.873069
O	-3.094045	5.451325	2.771598
H	-3.302993	6.383069	2.601858
H	-3.971425	5.079864	3.006637
H	-9.424006	0.825378	0.008456

^{5}**TS2**$_{B}$

Element	X	Y	Z
C	4.858419	3.638950	-2.640195
H	5.179156	4.691142	-2.720678
C	3.511988	3.582633	-1.911220
H	2.752756	4.097967	-2.529228

H	3.599285	4.137645	-0.958132
O	3.170770	2.229607	-1.696427
H	2.268262	2.172048	-1.326744
C	5.920014	2.870505	-1.874778
O	6.112745	3.087203	-0.663603
N	6.614499	1.940697	-2.555764
H	6.416192	1.804774	-3.542314
C	7.462961	0.939030	-1.938287
H	7.750695	1.278370	-0.939461
C	6.720201	-0.428476	-1.812845
H	7.412303	-1.112423	-1.289388
C	5.430926	-0.348900	-1.029071
H	4.729248	0.370792	-1.475153
H	5.645585	-0.058389	0.005913
H	4.976528	-1.346302	-1.005461
O	6.422474	-0.951551	-3.118124
H	7.118940	-0.671802	-3.746196
C	8.689794	0.620031	-2.787616
O	8.566817	0.568995	-4.014038
N	9.777938	0.189323	-2.109242
H	9.730541	0.199309	-1.086159
C	10.700265	-0.756307	-2.732553
H	10.436426	-0.759262	-3.794165
C	12.196560	-0.377669	-2.598693
H	12.310088	0.359287	-1.788670
H	12.780791	-1.262354	-2.300781
C	12.783997	0.197117	-3.891281
H	13.795897	0.594192	-3.707703
H	12.176194	1.052648	-4.238072
C	12.835991	-0.821686	-5.033721
O	12.140866	-1.833876	-5.050290
N	13.682080	-0.521461	-6.044964
H	13.724037	-1.143947	-6.844974
H	14.270801	0.301276	-6.037779
C	10.398551	-2.159796	-2.162923
O	10.151767	-2.328489	-0.964629
N	10.396109	-3.134509	-3.088076
H	10.770384	-2.873598	-4.003110
C	10.101838	-4.540080	-2.826580
H	10.439714	-4.787297	-1.807812
C	8.614222	-4.910632	-2.999434
H	8.220267	-4.381478	-3.884524
H	8.550564	-5.986945	-3.235751
C	7.753041	-4.624987	-1.760513

H	8.093196	-5.282279	-0.943373
H	7.921034	-3.599330	-1.395398
C	6.247930	-4.841849	-1.928927
H	6.047837	-5.790997	-2.457749
H	5.770641	-4.922635	-0.939472
N	5.640369	-3.743087	-2.676360
H	6.178891	-2.888574	-2.828337
C	4.348412	-3.678963	-3.038960
N	3.507726	-4.671742	-2.744258
H	2.536897	-4.620925	-3.068254
H	3.662725	-5.257130	-1.906712
N	3.936212	-2.613161	-3.747649
H	2.929344	-2.401099	-3.823625
H	4.602646	-1.858006	-3.884680
C	8.300707	-3.461198	1.900077
H	8.740868	-3.609477	0.903549
C	6.797530	-3.742252	1.837344
H	6.693999	-4.722258	1.326345
C	6.122366	-3.885785	3.203071
H	6.554948	-4.733741	3.758083
H	5.048376	-4.089049	3.072960
H	6.240848	-2.984191	3.816760
O	6.222207	-2.726018	1.034106
H	5.251537	-2.827252	1.004464
C	8.732042	-2.093543	2.411040
O	8.156035	-1.458337	3.298500
N	9.906707	-1.657270	1.879604
H	10.196423	-2.024665	0.970822
C	10.573069	-0.493222	2.393722
H	10.516374	-0.495337	3.493732
C	10.006962	0.833619	1.876256
O	9.298869	0.874891	0.858042
N	10.338662	1.928491	2.578729
H	10.909395	1.806285	3.410369
C	9.935358	3.292035	2.233917
H	9.092677	3.217876	1.535478
C	9.530928	4.101537	3.474844
H	8.739196	3.554545	4.011010
H	10.394876	4.162974	4.162106
C	9.047523	5.496146	3.133282
C	9.936811	6.479762	2.662395
H	11.001067	6.246486	2.559160
C	9.502727	7.762008	2.338136
H	10.199021	8.524439	1.983641

C	8.148792	8.104371	2.488028
O	7.781833	9.367218	2.172155
H	6.848206	9.507456	2.379430
C	7.248041	7.137151	2.959543
H	6.196135	7.401989	3.112685
C	7.698631	5.849509	3.276006
H	6.981282	5.112991	3.647292
C	-4.341466	1.850935	4.403531
H	-3.914389	1.824542	3.383118
C	-3.201665	1.966795	5.437691
H	-3.575162	1.550893	6.390580
C	-1.985604	1.130176	5.013847
H	-1.227753	1.080160	5.811884
H	-2.287634	0.103617	4.767616
H	-1.507362	1.566906	4.118744
C	-2.855982	3.449833	5.678415
H	-3.776246	3.969013	5.997865
H	-2.576386	3.920664	4.718328
C	-1.762500	3.701564	6.718820
H	-1.640743	4.780122	6.907728
H	-0.780624	3.314021	6.401803
H	-2.008585	3.225431	7.683114
C	-5.211404	0.610091	4.564661
O	-4.790318	-0.438956	5.065482
N	-6.487626	0.746710	4.140495
H	-6.772669	1.574944	3.616396
C	-7.442425	-0.341868	4.161373
H	-7.019780	-1.092070	4.852308
C	-8.808913	0.119668	4.674665
H	-9.510197	-0.721842	4.782034
H	-9.244687	0.870117	3.999105
H	-8.688712	0.575301	5.667951
C	-7.443684	-1.065474	2.803957
O	-6.393331	-1.235689	2.177147
N	-8.605333	-1.592203	2.372555
H	-9.472887	-1.491155	2.904748
C	-8.625204	-2.579804	1.307585
H	-7.967960	-3.415500	1.598627
C	-10.053511	-3.128104	1.115844
H	-10.682825	-2.339685	0.676272
H	-10.018076	-3.975970	0.417673
C	-10.688864	-3.511538	2.454467
O	-11.052055	-2.628434	3.229412
N	-10.793806	-4.828338	2.720242

H	-10.288193	-5.530719	2.178840
H	-11.151108	-5.088357	3.634190
C	-8.092016	-1.994999	-0.017463
O	-8.517580	-0.948290	-0.492182
N	-7.153114	-2.754569	-0.629936
C	-6.506041	-2.363358	-1.863034
C	-5.916729	-3.626009	-2.516588
C	-5.448999	-1.240166	-1.658884
O	-5.831832	-4.689130	-1.897540
C	-4.258177	-1.612400	-0.835952
C	-3.035951	-2.125591	-1.209673
N	-4.190211	-1.480341	0.537043
C	-2.976627	-1.900101	0.948451
N	-2.248605	-2.303234	-0.090860
H	-6.833346	-3.619802	-0.206404
H	-7.271961	-1.950122	-2.539499
H	-5.109057	-0.914419	-2.651147
H	-5.980166	-0.389412	-1.205298
H	-2.698288	-2.391594	-2.206779
H	-2.691381	-1.924964	2.003168
H	-4.968428	-1.221105	1.163865
N	-5.541057	-3.482568	-3.805425
H	-5.503905	-2.548499	-4.229785
C	-5.025096	-4.583183	-4.572905
H	-5.289802	-5.517472	-4.055003
C	-3.502459	-4.544947	-4.767450
O	-2.808909	-3.650517	-4.293730
N	-2.999599	-5.567734	-5.495070
C	-1.574973	-5.744965	-5.764675
C	-0.891852	-6.769236	-4.848457
C	-0.980300	-6.440340	-3.356646
C	-0.235266	-5.176120	-2.922089
O	0.710649	-4.774937	-3.636831
O	-0.621629	-4.634056	-1.841030
H	-3.639707	-6.282823	-5.824841
H	-1.097829	-4.763236	-5.649700
H	-1.328270	-7.768200	-5.023385
H	0.166703	-6.826743	-5.145424
H	-0.541793	-7.272588	-2.776082
H	-2.026161	-6.353542	-3.024160
C	-7.645546	2.809216	-4.970018
H	-8.450852	2.358484	-5.573277
C	-6.560183	1.780315	-4.636452
H	-7.015176	0.914139	-4.129525

H	-5.863536	2.236657	-3.916618
C	-5.773139	1.271195	-5.853344
H	-5.253453	2.103677	-6.355456
H	-6.459361	0.818108	-6.588098
C	-4.798314	0.180548	-5.419037
O	-5.232201	-0.876512	-4.951843
N	-3.485074	0.452287	-5.526202
H	-3.173539	1.329129	-5.923561
H	-2.769365	-0.203422	-5.181134
C	-8.200905	3.465328	-3.711708
O	-7.470143	3.841994	-2.800847
N	-9.556125	3.612322	-3.636620
H	-10.116838	3.371567	-4.445964
C	-10.160987	4.424620	-2.590674
H	-9.792596	5.461793	-2.619987
C	-9.902466	3.938408	-1.158566
O	-9.865954	4.738729	-0.233754
N	-9.731756	2.603324	-0.993450
H	-9.753465	2.002293	-1.808773
C	-9.464541	2.014607	0.303283
H	-10.252337	2.284028	1.023969
C	-8.148921	2.429754	0.960735
O	-7.956134	2.149518	2.142355
N	-7.252022	3.112923	0.214978
H	-7.452942	3.326614	-0.761941
C	-6.068425	3.699939	0.822131
H	-5.524745	2.921518	1.377848
C	-5.152053	4.317774	-0.247082
H	-5.753873	5.020492	-0.846966
H	-4.383581	4.908398	0.275170
C	-4.453932	3.308615	-1.177677
H	-5.217130	2.610649	-1.565001
C	-3.864962	4.046841	-2.386239
H	-3.143984	4.819189	-2.060461
H	-3.347151	3.353683	-3.069446
H	-4.655768	4.558603	-2.955939
C	-3.386792	2.490119	-0.440668
H	-2.604965	3.145803	-0.021725
H	-3.814068	1.916387	0.396925
H	-2.906254	1.764286	-1.114257
C	-6.395679	4.760566	1.892517
O	-5.573438	4.985543	2.785940
N	-7.555592	5.431458	1.764404
H	-8.217885	5.158955	1.036720

C	-7.960116	6.482359	2.684923
H	-7.360418	6.364839	3.598312
C	-7.789743	7.904388	2.112649
H	-8.197855	8.608756	2.857580
H	-8.416341	8.004466	1.211301
C	-6.359614	8.277427	1.785018
C	-5.438059	8.557602	2.807674
H	-5.764589	8.527832	3.851085
C	-4.111228	8.880814	2.513239
H	-3.415880	9.109440	3.325324
C	-3.677095	8.926018	1.182115
H	-2.643238	9.193793	0.948229
C	-4.583707	8.651849	0.153782
H	-4.259672	8.697760	-0.889270
C	-5.911276	8.331186	0.456067
H	-6.615985	8.127867	-0.355314
C	-8.117724	-6.851181	-0.767763
H	-7.404537	-6.455278	-1.502175
C	-7.838369	-6.377330	0.644140
O	-8.686286	-6.445806	1.528974
N	-6.578129	-5.895369	0.875748
C	-6.135346	-5.465692	2.196346
C	-4.898662	-6.223784	2.723173
C	-3.675259	-6.004684	1.889970
C	-2.754496	-4.980611	1.878799
N	-3.338466	-6.793639	0.802751
C	-2.268525	-6.235171	0.185456
N	-1.899217	-5.129859	0.805010
H	-5.984171	-5.722030	0.065760
H	-6.985396	-5.625057	2.873649
H	-5.129417	-7.299274	2.802726
H	-4.698186	-5.863755	3.743990
H	-2.690682	-4.148657	2.578593
H	-1.801982	-6.644401	-0.704769
H	-3.803243	-7.650823	0.525947
Fe	-0.528891	-3.605922	-0.118581
O	0.489865	-1.927234	-0.917613
C	1.145262	-4.088975	1.709268
O	0.294167	-3.153214	1.801640
C	2.023597	-4.447285	2.879463
H	2.957527	-4.902180	2.525243
H	2.230667	-3.565242	3.499192
C	2.500387	1.291826	3.308822
C	1.748872	2.623512	3.579192

O	1.976336	3.204822	4.619579
S	0.589403	3.155579	2.369661
C	0.008125	4.693987	3.152098
C	2.798687	0.932325	1.844899
N	3.205814	-0.464271	1.714062
C	2.892756	-1.347035	0.729495
O	3.419096	-2.455199	0.650565
H	4.050877	-0.734164	2.242846
C	1.988236	-0.870960	-0.366352
C	0.545569	-0.660979	-0.339751
C	0.119446	0.573834	-1.131276
O	0.587247	1.657022	-0.716012
N	-0.666282	0.472906	-2.191075
H	-0.849641	1.354391	-2.689532
H	-1.021919	-0.397968	-2.600818
N	3.754448	1.400770	4.112495
H	4.337907	2.246083	3.758036
H	3.517927	1.603823	5.089726
H	1.881064	0.487767	3.741873
H	-0.104329	4.526405	4.230042
H	-0.965739	4.937563	2.697683
H	3.581400	1.606771	1.455119
H	1.906332	1.123651	1.232348
H	2.492526	-0.701002	-1.321223
H	0.085093	-0.622278	0.662152
H	4.402469	0.580815	4.022335
H	1.490642	-5.183681	3.503744
O	1.219602	-4.755218	0.632956
H	-5.906744	-4.386081	2.188772
H	-9.142096	-6.564014	-1.043633
H	-8.080887	-7.953025	-0.776811
H	-1.456748	-6.049059	-6.816785
H	-5.502326	-4.604214	-5.568230
H	-4.982879	2.745988	4.423877
H	10.712004	-5.129704	-3.526552
H	4.756192	3.240051	-3.660868
H	-9.017874	6.324979	2.949963
H	-11.248943	4.450984	-2.755419
H	-7.215674	3.626178	-5.577780
H	10.756886	3.799064	1.698331
H	11.639550	-0.538423	2.115326
H	8.779925	-4.201407	2.565238
H	0.742339	5.491098	2.972984
O	3.533876	-5.894427	-0.200189

H	3.402549	-6.838009	-0.035088
H	2.694525	-5.471193	0.118051
O	5.322017	3.293424	3.166672
H	5.106850	3.990076	2.490072
H	6.043035	2.784957	2.735095
O	5.120552	4.988931	1.094195
H	5.761024	5.708289	1.205848
H	5.489604	4.421062	0.381509
O	-3.046983	-2.302300	4.050937
H	-2.405013	-2.374613	4.769208
H	-3.738280	-1.704077	4.418034
O	1.100704	-2.164919	-3.687993
H	0.914841	-3.140687	-3.764963
H	0.913319	-2.019928	-2.736054
O	-1.456659	-1.272466	-4.293073
H	-1.913377	-2.138819	-4.323090
H	-0.501241	-1.497746	-4.356376
O	-0.495569	3.270690	-2.712524
H	-0.126222	3.038692	-1.836663
H	-1.201767	3.905682	-2.531615
O	5.662186	-0.282819	3.158749
H	6.041726	0.360535	2.514874
H	6.427210	-0.874844	3.346786
O	6.773096	1.660107	1.535635
H	6.491389	2.118216	0.707394
H	7.687820	1.358307	1.336355
O	-2.932761	5.490415	2.120656
H	-3.068670	6.446111	2.029006
H	-3.801410	5.190257	2.468723
H	-9.444010	0.919783	0.200174

$^{5}\mathbf{P}_{B}$

Element	X	Y	Z
C	4.900666	3.641548	-3.813173
H	5.305414	4.666623	-3.854168
C	3.409586	3.694187	-3.469710
H	2.891663	4.265400	-4.264547
H	3.273851	4.250751	-2.522802
O	2.939943	2.373571	-3.369628
H	1.978769	2.370774	-3.193219
C	5.684117	2.869733	-2.767002
O	5.659439	3.210764	-1.569280
N	6.390219	1.811940	-3.201219

H	6.383334	1.583632	-4.191398
C	7.101977	0.879583	-2.347864
H	7.324923	1.362119	-1.391084
C	6.277784	-0.412900	-2.046303
H	6.880780	-0.971877	-1.307069
C	4.906837	-0.115203	-1.472432
H	4.293774	0.456210	-2.185043
H	5.007777	0.459325	-0.540977
H	4.376388	-1.048316	-1.241243
O	6.124589	-1.222217	-3.220441
H	6.821466	-0.993118	-3.868780
C	8.370857	0.399390	-3.045692
O	8.305616	0.139048	-4.251392
N	9.429449	0.118107	-2.259573
H	9.328349	0.277284	-1.252000
C	10.420610	-0.856511	-2.703569
H	10.297071	-0.927974	-3.789031
C	11.882557	-0.451211	-2.388022
H	11.879132	0.325666	-1.607737
H	12.425877	-1.312575	-1.969647
C	12.642388	0.067284	-3.612060
H	13.614078	0.489352	-3.306594
H	12.085364	0.895227	-4.087006
C	12.871838	-1.010179	-4.675717
O	12.237452	-2.062017	-4.698813
N	13.805261	-0.720890	-5.608940
H	13.971545	-1.388605	-6.354565
H	14.346463	0.133890	-5.592631
C	10.046674	-2.229453	-2.103252
O	9.505351	-2.309939	-0.995187
N	10.329631	-3.277466	-2.889741
H	10.885031	-3.069506	-3.724925
C	10.043882	-4.671083	-2.554893
H	10.357260	-4.866165	-1.515628
C	8.567941	-5.089228	-2.746745
H	8.149671	-4.513772	-3.590534
H	8.540921	-6.146811	-3.060412
C	7.707172	-4.929564	-1.483207
H	8.072333	-5.638939	-0.721786
H	7.862275	-3.928430	-1.054671
C	6.209385	-5.175505	-1.655766
H	6.026830	-6.110536	-2.216226
H	5.747550	-5.304671	-0.663354
N	5.554904	-4.065326	-2.350056

H	6.096197	-3.250608	-2.639027
C	4.247199	-4.046416	-2.668858
N	3.434819	-5.003309	-2.216522
H	2.445228	-5.000434	-2.495073
H	3.620737	-5.444805	-1.302680
N	3.789836	-3.081605	-3.482867
H	2.782976	-2.862193	-3.474617
H	4.435473	-2.334043	-3.731427
C	6.132162	-2.384793	1.470889
H	5.623013	-1.785508	0.697846
C	5.050570	-2.916986	2.441688
H	4.514502	-3.706787	1.881685
C	5.636506	-3.541740	3.703030
H	6.365769	-4.328143	3.450515
H	4.838254	-3.995978	4.307270
H	6.137167	-2.779673	4.317590
O	4.140654	-1.901212	2.830550
H	3.586974	-1.644859	2.066658
C	7.217851	-1.513580	2.081873
O	7.017848	-0.706846	2.995003
N	8.458602	-1.658885	1.556402
H	8.620315	-2.187092	0.700115
C	9.555011	-0.854650	2.027952
H	9.707340	-1.007097	3.108727
C	9.389294	0.641425	1.739186
O	8.857861	1.038195	0.687338
N	9.912509	1.461639	2.660881
H	10.258142	1.036026	3.515654
C	9.963567	2.919195	2.584892
H	9.549099	3.214374	1.612020
C	9.201872	3.597719	3.737794
H	8.131704	3.349371	3.652519
H	9.549734	3.161319	4.691600
C	9.400870	5.098585	3.764343
C	10.603727	5.650691	4.242195
H	11.388910	4.991710	4.626560
C	10.821176	7.025148	4.256575
H	11.752584	7.449711	4.636131
C	9.823565	7.896266	3.789549
O	10.081194	9.224614	3.828519
H	9.315954	9.721165	3.509489
C	8.617330	7.362819	3.314989
H	7.828598	8.034055	2.960276
C	8.412987	5.979764	3.302951

H	7.457016	5.591590	2.940026
C	-3.656845	0.774014	4.840852
H	-3.792773	1.400040	3.941958
C	-2.183876	0.393387	5.041916
H	-2.146454	-0.293300	5.906177
C	-1.627694	-0.357679	3.824997
H	-0.573288	-0.644358	3.961840
H	-2.189258	-1.284928	3.650452
H	-1.693748	0.262433	2.913065
C	-1.356599	1.645548	5.392166
H	-1.878091	2.202318	6.189830
H	-1.339285	2.325703	4.519363
C	0.074041	1.361455	5.856901
H	0.596171	2.294883	6.125542
H	0.670050	0.846420	5.084492
H	0.083933	0.715245	6.749965
C	-4.625971	-0.405469	4.784745
O	-4.338486	-1.523594	5.211649
N	-5.852513	-0.090390	4.293215
H	-5.993208	0.824922	3.849715
C	-6.941651	-1.044579	4.219360
H	-6.627027	-1.901372	4.839820
C	-8.247873	-0.464263	4.765632
H	-9.063565	-1.203057	4.744110
H	-8.569710	0.422239	4.196740
H	-8.103052	-0.155702	5.810506
C	-7.007315	-1.637973	2.800614
O	-5.976282	-2.043157	2.259067
N	-8.198030	-1.746394	2.176620
H	-9.079051	-1.510286	2.644091
C	-8.322676	-2.569681	0.980242
H	-7.907256	-3.564469	1.206837
C	-9.808356	-2.734448	0.602984
H	-10.188680	-1.772879	0.226534
H	-9.890603	-3.481249	-0.198691
C	-10.655549	-3.103356	1.823036
O	-10.878660	-2.252477	2.684053
N	-11.094737	-4.373475	1.893641
H	-10.712059	-5.108882	1.297103
H	-11.601987	-4.643443	2.730701
C	-7.535214	-1.987612	-0.220044
O	-7.696031	-0.834746	-0.611968
N	-6.706034	-2.870549	-0.822380
C	-5.885127	-2.573431	-1.980207

C	-5.516542	-3.906760	-2.652110
C	-4.604085	-1.747350	-1.640806
O	-5.564832	-4.971534	-2.031486
C	-3.646241	-2.394854	-0.693847
C	-2.473710	-3.093314	-0.896245
N	-3.847759	-2.423958	0.671430
C	-2.847682	-3.112184	1.242986
N	-1.988355	-3.537228	0.317389
H	-6.592631	-3.803752	-0.436273
H	-6.468094	-1.964911	-2.687979
H	-4.090063	-1.536207	-2.586649
H	-4.953040	-0.784037	-1.235049
H	-1.968846	-3.299563	-1.832930
H	-2.818403	-3.324492	2.315083
H	-4.670289	-2.092293	1.197647
N	-5.133145	-3.801328	-3.941405
H	-5.034332	-2.860675	-4.345534
C	-4.552232	-4.907513	-4.655752
H	-4.876548	-5.844226	-4.177475
C	-3.015047	-4.847277	-4.644797
O	-2.425822	-3.967328	-4.024883
N	-2.369059	-5.808264	-5.340141
C	-0.910384	-5.866701	-5.436732
C	-0.274107	-7.046613	-4.690774
C	-0.609749	-7.141938	-3.202386
C	-0.089106	-6.028937	-2.285367
O	0.641679	-5.126714	-2.758420
O	-0.440043	-6.135703	-1.073306
H	-2.917627	-6.508553	-5.828959
H	-0.532297	-4.924438	-5.023180
H	-0.570005	-7.993062	-5.177268
H	0.817003	-6.961776	-4.820632
H	-0.220473	-8.089762	-2.792767
H	-1.702094	-7.191035	-3.051630
C	-7.407726	2.527611	-4.289953
H	-8.200329	2.024897	-4.868012
C	-6.224743	1.576469	-4.063743
H	-6.582051	0.672603	-3.543483
H	-5.503079	2.068196	-3.391380
C	-5.520400	1.163573	-5.366798
H	-5.029895	2.034216	-5.830838
H	-6.261379	0.774448	-6.083883
C	-4.529139	0.035441	-5.109570
O	-4.950990	-1.091792	-4.820752

N	-3.221840	0.337493	-5.169466
H	-2.887322	1.287670	-5.324010
H	-2.527053	-0.357791	-4.866884
C	-7.943278	3.083054	-2.978628
O	-7.240391	3.747923	-2.223884
N	-9.237580	2.781256	-2.669362
H	-9.799069	2.295646	-3.358571
C	-9.915750	3.416587	-1.551802
H	-9.973267	4.508813	-1.680256
C	-9.262164	3.202974	-0.183319
O	-9.358311	4.067630	0.680003
N	-8.604613	2.035402	0.013516
H	-8.552807	1.325330	-0.711849
C	-7.960606	1.738713	1.271469
H	-8.646615	1.957495	2.106616
C	-6.688872	2.524383	1.601674
O	-6.123607	2.325546	2.678345
N	-6.285278	3.460813	0.718381
H	-6.754369	3.538853	-0.184863
C	-5.319689	4.489086	1.075951
H	-4.473286	4.018582	1.596946
C	-4.832976	5.237743	-0.176990
H	-5.718910	5.526752	-0.766028
H	-4.345462	6.165803	0.155078
C	-3.844038	4.463934	-1.067867
H	-4.258828	3.456552	-1.258958
C	-3.707883	5.169202	-2.423043
H	-3.304786	6.188861	-2.300940
H	-3.027392	4.609282	-3.083671
H	-4.685448	5.246070	-2.924648
C	-2.476379	4.311101	-0.386448
H	-2.098342	5.294562	-0.062419
H	-2.530403	3.670706	0.508708
H	-1.735666	3.865543	-1.069706
C	-5.867992	5.506368	2.099593
O	-5.073061	6.176710	2.762056
N	-7.204761	5.643701	2.189583
H	-7.819353	5.055525	1.624857
C	-7.830316	6.622747	3.064486
H	-7.101683	6.876192	3.847183
C	-8.293837	7.899001	2.331611
H	-8.819855	8.528678	3.069530
H	-9.039152	7.619246	1.569157
C	-7.174709	8.689553	1.687573

C	-6.289132	9.449313	2.469836
H	-6.426744	9.490227	3.554146
C	-5.236824	10.157303	1.884702
H	-4.568911	10.753342	2.512459
C	-5.047036	10.116364	0.497476
H	-4.233913	10.682469	0.035123
C	-5.922998	9.367874	-0.294179
H	-5.792788	9.339786	-1.379207
C	-6.975928	8.662885	0.298513
H	-7.661665	8.087053	-0.329643
C	-8.457502	-6.582635	-1.587850
H	-7.520962	-6.424609	-2.137310
C	-8.323690	-6.349582	-0.096748
O	-9.308640	-6.282604	0.633915
N	-7.047736	-6.243445	0.383446
C	-6.755166	-6.105219	1.804731
C	-5.821288	-7.200923	2.361136
C	-4.442675	-7.150548	1.780524
C	-3.361018	-6.357186	2.088114
N	-4.060493	-7.853258	0.647514
C	-2.808708	-7.471113	0.305157
N	-2.362073	-6.559655	1.153903
H	-6.295368	-6.140159	-0.296345
H	-7.722701	-6.141936	2.323600
H	-6.276735	-8.192360	2.201283
H	-5.748872	-7.056513	3.450050
H	-3.262497	-5.642057	2.906565
H	-2.259799	-7.846575	-0.554557
H	-4.615210	-8.555842	0.171501
Fe	-0.777387	-5.199427	0.701517
O	1.583591	0.037547	-1.742977
C	0.738681	-4.626388	2.693181
O	-0.478849	-4.462651	2.904116
C	1.779208	-4.347950	3.745216
H	2.519924	-5.161076	3.766367
H	2.321681	-3.419798	3.498229
C	2.261057	3.150163	3.115849
C	1.607004	4.524903	3.406604
O	2.078649	5.215608	4.285814
S	0.169662	4.936648	2.484381
C	-0.264841	6.517969	3.277146
C	2.270034	2.678214	1.653188
N	2.587511	1.255114	1.582673
C	2.251936	0.375258	0.606953

O	2.516138	-0.822507	0.677478
H	3.327618	0.910968	2.214480
C	1.634832	0.944423	-0.659944
C	0.342245	0.482800	-1.220571
C	-0.420208	1.404723	-2.153551
O	0.141225	2.371819	-2.689915
N	-1.709255	1.090824	-2.330504
H	-2.211637	1.630409	-3.037097
H	-1.973636	0.106891	-2.253032
N	3.633891	3.236259	3.688429
H	4.274191	3.862178	3.082598
H	3.574579	3.685570	4.611027
H	1.694865	2.415201	3.712651
H	-0.505219	6.341930	4.333529
H	-1.142634	6.900366	2.729279
H	2.963764	3.306079	1.064769
H	1.263489	2.848408	1.245313
H	1.963575	1.948789	-0.949011
H	-0.237819	-0.276495	-0.680543
H	4.135397	2.311163	3.720446
H	1.311891	-4.240739	4.731948
O	1.125865	-5.033787	1.526652
H	-6.299985	-5.120326	2.007318
H	-9.239252	-5.919006	-1.984959
H	-8.809410	-7.615411	-1.742570
H	-0.629845	-5.909808	-6.502125
H	-4.909992	-4.919545	-5.699090
H	-3.993609	1.401475	5.685800
H	10.687277	-5.279768	-3.205294
H	5.033893	3.188033	-4.806857
H	-8.700082	6.150600	3.548660
H	-10.946266	3.032241	-1.505741
H	-7.079485	3.399010	-4.881859
H	11.019538	3.237555	2.599642
H	10.464181	-1.191807	1.506090
H	6.600556	-3.233833	0.948683
H	0.585718	7.206894	3.197126
O	3.557407	-5.718828	0.529885
H	3.696842	-6.587694	0.930977
H	2.687730	-5.417960	0.904503
O	5.343964	4.575948	2.155785
H	4.945232	4.965397	1.340292
H	5.936212	3.873054	1.804720
O	4.184850	5.089071	-0.237956

H	4.121414	5.952919	-0.667238
H	4.728945	4.523725	-0.836146
O	-3.062404	-3.892939	4.240525
H	-2.095047	-3.850877	4.299829
H	-3.421570	-3.103280	4.691318
O	1.022782	-2.493530	-2.922883
H	0.770531	-3.418778	-2.671635
H	1.276674	-2.020512	-2.112735
O	-1.483402	-1.444464	-3.679994
H	-1.918852	-2.304422	-3.850585
H	-0.542475	-1.702617	-3.563043
O	-1.812103	2.936141	-4.668868
H	-0.994523	2.957959	-4.127203
H	-2.014659	3.861075	-4.862945
O	5.018290	1.066120	2.981719
H	5.563233	1.442609	2.253528
H	5.480197	0.224612	3.211471
O	6.467252	2.362568	0.950661
H	6.195974	2.586708	0.031371
H	7.377682	1.992835	0.875278
O	-2.863411	7.364965	1.656989
H	-3.242513	8.234999	1.463975
H	-3.594014	6.911090	2.137468
H	-7.733505	0.667167	1.289046

$^1R_{(A)}$

Element	X	Y	Z
C	-2.424850	-3.334833	-5.271610
C	-3.510885	-3.270306	-4.199973
C	-1.134525	-2.697377	-4.732565
O	-3.856091	-2.195937	-3.699103
C	0.035016	-2.761887	-5.715726
S	1.435153	-1.686441	-5.207089
N	-4.005226	-4.457163	-3.804581
C	-4.977681	-4.625115	-2.747495
C	-5.462184	-6.079766	-2.779950
O	-5.412599	-6.723680	-3.816836
N	-5.958145	-6.540002	-1.599455
C	-6.357498	-7.928554	-1.436435
C	-7.733449	-8.160858	-0.803683
O	-8.035058	-9.271514	-0.400643
N	-8.563351	-7.081320	-0.769944
C	-9.929026	-7.148439	-0.278371

C	-10.189799	-6.384350	1.035514
C	-9.368923	-6.942845	2.203545
C	-9.985836	-4.871677	0.876375
C	2.075456	-2.605053	-3.824003
O	1.781250	-3.748249	-3.563202
C	3.004483	-1.790831	-2.911271
N	4.065034	-2.695562	-2.414412
C	2.204520	-1.199043	-1.723086
N	1.214413	-0.248913	-2.184990
C	0.712447	0.865806	-1.558953
O	-0.070394	1.603111	-2.142787
C	1.139117	1.110293	-0.145670
C	1.031382	2.334482	0.395147
C	1.335748	2.683783	1.816651
O	1.417135	3.882105	2.153874
N	1.508567	1.682338	2.685851
H	-2.780926	-2.774682	-6.153169
H	-2.246284	-4.374483	-5.588452
H	-0.843858	-3.206621	-3.800310
H	-1.349324	-1.650110	-4.473111
H	0.426737	-3.782917	-5.832935
H	-0.239945	-2.379976	-6.711031
H	-3.816254	-5.301808	-4.341002
H	-4.548970	-4.378945	-1.762481
H	-5.645394	-6.049251	-0.758928
H	-6.364046	-8.392874	-2.433880
H	-8.205974	-6.209047	-1.140841
H	-10.149041	-8.216695	-0.139437
H	-11.260552	-6.557964	1.254307
H	3.493004	-0.957747	-3.437327
H	1.753570	-2.033960	-1.152374
H	2.931331	-0.700957	-1.065910
H	0.830511	-0.378247	-3.118992
H	1.491366	0.258458	0.442687
H	0.711819	3.178124	-0.221564
H	1.685237	1.906383	3.669970
H	1.384531	0.689082	2.463320
H	4.846106	-2.789606	-3.116960
H	4.516040	-2.356938	-1.517346
H	3.751813	-3.670576	-2.252681
H	-8.922787	-4.624004	0.724604
H	-10.571008	-4.470560	0.030919
H	-10.301328	-4.335672	1.785855
H	-8.294984	-6.746642	2.060162

H	-9.508754	-8.029963	2.310310
H	-9.658812	-6.461184	3.151143
H	-5.844323	-3.958067	-2.902862
H	-10.610660	-6.766907	-1.060425
H	-5.618590	-8.479336	-0.834268

^{3}IM$_{(A)}$

Element	X	Y	Z
O	1.405161	4.068832	-0.465706
C	-2.437237	-3.174037	-5.094696
C	-3.577528	-3.198515	-4.079390
C	-1.210468	-2.476715	-4.484398
O	-4.023152	-2.156543	-3.589028
C	-0.012295	-2.415482	-5.433602
S	1.304537	-1.289098	-4.825336
N	-4.009372	-4.420589	-3.720128
C	-5.021971	-4.660845	-2.715759
C	-5.416780	-6.140569	-2.785323
O	-5.300822	-6.763236	-3.829694
N	-5.914566	-6.648555	-1.625204
C	-6.231578	-8.061229	-1.491631
C	-7.574237	-8.379588	-0.826418
O	-7.795744	-9.505289	-0.413413
N	-8.468448	-7.353166	-0.773765
C	-9.807681	-7.495405	-0.228530
C	-10.066325	-6.703544	1.069356
C	-9.164762	-7.167440	2.218995
C	-9.968326	-5.187851	0.851100
C	2.030587	-2.289051	-3.557234
O	1.777336	-3.459073	-3.370467
C	2.987802	-1.530581	-2.627696
N	4.129389	-2.421083	-2.321470
C	2.255621	-1.144769	-1.312114
N	1.034654	-0.419182	-1.549171
C	0.631282	0.827856	-1.125843
O	-0.531550	1.183587	-1.331786
C	1.577820	1.707948	-0.432929
C	1.050190	2.930353	0.253366
C	1.453842	3.076099	1.736248
O	1.579204	4.206231	2.234992
N	1.580593	1.954188	2.447531
H	-2.783925	-2.614396	-5.980329
H	-2.182623	-4.193063	-5.426286

H	-0.912549	-3.013436	-3.569261
H	-1.503053	-1.459564	-4.183911
H	0.443005	-3.403854	-5.592464
H	-0.288116	-1.997781	-6.413676
H	-3.734419	-5.243852	-4.251967
H	-4.657974	-4.401242	-1.708493
H	-5.648605	-6.155187	-0.770137
H	-6.238683	-8.498922	-2.501102
H	-8.176522	-6.462831	-1.158977
H	-9.948573	-8.570723	-0.047837
H	-11.113672	-6.936653	1.339819
H	3.379029	-0.613840	-3.089877
H	2.048865	-2.069963	-0.743759
H	2.945483	-0.541733	-0.708910
H	0.260822	-0.931195	-1.963433
H	2.657557	1.581429	-0.543190
H	-0.049562	2.815203	0.287219
H	1.706376	2.032079	3.462987
H	1.449535	1.003402	2.084639
H	4.849745	-2.422749	-3.089082
H	4.639212	-2.152380	-1.431622
H	3.867354	-3.421233	-2.223374
H	-8.932887	-4.878789	0.634244
H	-10.619813	-4.855103	0.024794
H	-10.265770	-4.639274	1.758403
H	-8.109652	-6.926873	2.014482
H	-9.243719	-8.254364	2.378424
H	-9.431699	-6.656809	3.157615
H	-5.917563	-4.043984	-2.909577
H	-10.543270	-7.187485	-0.994052
H	-5.448974	-8.585369	-0.921482

$^{1}P_{(A)}$

Element	X	Y	Z
O	2.459064	0.810498	-0.557995
C	-2.656496	-3.532725	-5.465900
C	-3.664784	-3.473104	-4.317892
C	-1.346992	-2.878255	-4.999378
O	-3.970572	-2.400830	-3.789430
C	-0.214676	-2.880014	-6.028655
S	1.178645	-1.820679	-5.460192
N	-4.122537	-4.661595	-3.883529
C	-5.002290	-4.829123	-2.748265

C	-5.410486	-6.306510	-2.688386
O	-5.389952	-6.990772	-3.700791
N	-5.807796	-6.740430	-1.462901
C	-6.138647	-8.135808	-1.220103
C	-7.475677	-8.395570	-0.517750
O	-7.704165	-9.493184	-0.038367
N	-8.356612	-7.356790	-0.510523
C	-9.691965	-7.454562	0.054159
C	-9.928080	-6.598223	1.314665
C	-9.019303	-7.016342	2.476139
C	-9.816344	-5.096038	1.022226
C	1.886162	-2.869459	-4.204958
O	1.695265	-4.060120	-4.114347
C	2.666516	-2.104987	-3.128740
N	3.829075	-2.909495	-2.700746
C	1.718562	-1.880823	-1.916961
N	0.607822	-1.017845	-2.250352
C	0.295197	0.208664	-1.749733
O	-0.596075	0.896855	-2.221330
C	1.037991	0.652995	-0.504901
C	1.636890	1.991736	-0.468066
C	1.725242	2.782263	0.814237
O	1.577406	4.017969	0.780728
N	1.971509	2.098581	1.925779
H	-3.073676	-2.975964	-6.322130
H	-2.487610	-4.570803	-5.792603
H	-0.999047	-3.403139	-4.094133
H	-1.580938	-1.843078	-4.707131
H	0.182232	-3.887681	-6.218856
H	-0.522484	-2.436340	-6.987737
H	-3.975634	-5.511401	-4.426195
H	-4.514004	-4.517902	-1.810313
H	-5.490898	-6.195272	-0.657470
H	-6.165358	-8.646963	-2.194013
H	-8.059722	-6.491865	-0.946203
H	-9.844709	-8.517944	0.287970
H	-10.974960	-6.806363	1.606443
H	3.039631	-1.127631	-3.474967
H	1.355758	-2.865523	-1.572350
H	2.304749	-1.440822	-1.101191
H	0.037823	-1.270516	-3.054201
H	0.677883	0.194164	0.426149
H	1.583323	2.606265	-1.371840
H	1.943070	2.555680	2.843971

H	1.986365	1.070028	1.943082
H	4.582435	-2.921971	-3.439990
H	4.253932	-2.528055	-1.811314
H	3.631566	-3.916084	-2.536538
H	-8.779752	-4.809188	0.781808
H	-10.472001	-4.796652	0.186525
H	-10.100664	-4.501125	1.904306
H	-7.964268	-6.795663	2.250204
H	-9.106627	-8.093768	2.687153
H	-9.272490	-6.459966	3.392384
H	-5.912627	-4.212500	-2.858079
H	-10.430941	-7.174103	-0.718539
H	-5.353076	-8.622642	-0.622313

$^{1}R_{(B)}$

Element	X	Y	Z
C	2.834627	3.771139	3.622652
C	2.133678	5.129899	3.874346
O	2.696546	5.961514	4.555340
S	0.522543	5.309642	3.195603
C	0.062557	6.942785	3.852119
C	2.610296	3.137898	2.242113
N	2.992798	1.733488	2.252348
C	2.411863	0.704330	1.579946
O	2.737148	-0.465100	1.806785
H	3.841498	1.470450	2.771924
C	1.444896	1.017681	0.483794
C	0.837682	0.010980	-0.159021
C	0.035260	0.210363	-1.401768
O	0.030334	1.302609	-2.004517
N	-0.630630	-0.871933	-1.814217
H	-1.103867	-0.914834	-2.730265
H	-0.539848	-1.752050	-1.310990
N	4.279587	3.996413	3.907039
H	4.750596	4.553362	3.107752
H	4.358476	4.573787	4.754260
H	2.441002	3.078953	4.386801
H	-0.030698	6.890271	4.944691
H	-0.905837	7.167139	3.373873
H	3.141892	3.721481	1.469813
H	1.538989	3.216160	2.015322
H	1.315169	2.041730	0.126044
H	0.982482	-1.009740	0.207926

H	4.840156	3.106548	3.974575
H	0.830166	7.676267	3.574157

$^{3}IM_{(B)}$

Element	X	Y	Z
O	0.308076	-1.840602	-0.936712
C	2.886857	2.208607	3.422564
C	2.092692	3.489841	3.781398
O	2.529622	4.244235	4.622896
S	0.542227	3.691579	2.967347
C	-0.053084	5.210581	3.771697
C	2.908422	1.840295	1.926153
N	3.219515	0.443080	1.686791
C	2.530986	-0.439631	0.883253
O	2.839288	-1.642625	0.873771
H	4.098514	0.090476	2.083589
C	1.515451	0.132112	0.002261
C	0.293246	-0.488538	-0.605561
C	-0.143548	0.388712	-1.830238
O	0.308758	1.530637	-2.024898
N	-1.080219	-0.154068	-2.601626
H	-1.418344	0.357097	-3.416467
H	-1.336912	-1.125421	-2.489587
N	4.255704	2.410009	3.968315
H	4.761598	3.204926	3.437742
H	4.200834	2.704316	4.949182
H	2.413515	1.378285	3.973435
H	-0.002452	5.088224	4.860686
H	-1.097190	5.327078	3.438861
H	3.594547	2.526747	1.399731
H	1.907983	2.050698	1.531957
H	1.606818	1.190181	-0.245365
H	-0.522927	-0.294313	0.135286
H	4.884141	1.570156	3.829778
H	0.566995	6.060984	3.459254

$^{1}P_{(B)}$

Element	X	Y	Z
O	1.583591	0.037547	-1.742977
C	2.261057	3.150163	3.115849
C	1.607004	4.524903	3.406604
O	2.078649	5.215608	4.285814

S	0.169662	4.936648	2.484381
C	-0.264841	6.517969	3.277146
C	2.270034	2.678214	1.653188
N	2.587511	1.255114	1.582673
C	2.251936	0.375258	0.606953
O	2.516138	-0.822507	0.677478
H	3.327618	0.910968	2.214480
C	1.634832	0.944423	-0.659944
C	0.342245	0.482800	-1.220571
C	-0.420208	1.404723	-2.153551
O	0.141225	2.371819	-2.689915
N	-1.709255	1.090824	-2.330504
H	-2.211637	1.630409	-3.037097
H	-1.973636	0.106891	-2.253032
N	3.633891	3.236259	3.688429
H	4.274191	3.862178	3.082598
H	3.574579	3.685570	4.611027
H	1.694865	2.415201	3.712651
H	-0.505219	6.341930	4.333529
H	-1.142634	6.900366	2.729279
H	2.963764	3.306079	1.064769
H	1.263489	2.848408	1.245313
H	1.963575	1.948789	-0.949011
H	-0.237819	-0.276495	-0.680543
H	4.135397	2.311163	3.720446
H	0.585718	7.206894	3.197126
